# Sovereign states in the Caribbean have lower social-ecological vulnerability to coral bleaching than overseas territories

**DOI:** 10.1098/rspb.2018.2365

**Published:** 2019-02-20

**Authors:** Katherine J. Siegel, Reniel B. Cabral, Jennifer McHenry, Elena Ojea, Brandon Owashi, Sarah E. Lester

**Affiliations:** 1 Department of Environmental Science, Policy, and Management, University of California, Berkeley, 326 Mulford Hall, Berkeley, CA 94720-3114, USA; 2 Sustainable Fisheries Group, Bren School of Environmental Science and Management and Marine Science Institute, University of California, Santa Barbara, CA 93106-5131, USA; 3 Department of Geography, Florida State University, 323 Bellamy Building, Tallahassee, FL 32306-2190, USA; 4 Future Oceans Lab, University of Vigo, Spain

**Keywords:** vulnerability, coral bleaching, Caribbean, social-ecological

## Abstract

Coral reef social-ecological systems worldwide face major impacts from climate change, and spatial variation in vulnerability is driven by differential exposure to climatic threats, ecological and socio-economic sensitivity to those threats, ecological recovery potential, and socio-economic adaptive capacity. We assess variation in social-ecological vulnerability to climate change-induced coral bleaching, specifically for reef-based fisheries and tourism, of islands throughout the insular Caribbean, thus providing the first region-wide quantitative analysis of island-scale social-ecological vulnerability to coral bleaching. We show that different components of vulnerability have distinct spatial patterns and that variability in overall vulnerability is driven more by socio-economic than ecological components. Importantly, we find that sovereign islands are less vulnerable on average than overseas territories and that the presence of fisheries management regulations is a significant predictor of adaptive capacity and socio-economic sensitivity, with important implications for island-level governance and policies to reduce climate vulnerability.

## 1. Introduction

Many marine ecosystems are experiencing the effects of climate change [1,2], and coral reef systems, including those in the Caribbean, may be particularly at risk of climate change impacts [3–6]. The predicted temperature increase in the Caribbean by the end of the century is approximately 2–3°C [7], with more frequent and widespread bleaching events expected [3,8–10]. Additionally, many Caribbean reefs are already degraded, potentially making them more vulnerable to future climate pressures [11,12]. Changes to the natural system will impact human communities, as people rely on healthy reefs for services including seafood, tourism, and coastal protection [13]. However, the effects of climate impacts on both the ecological and social components of reef systems are complex. For example, ecological vulnerability will impact socio-economic exposure to climate-induced threats; ecological systems can demonstrate both fragility and remarkable recovery potential; and social systems can vary considerably in their sensitivity and adaptive capacity [14]. As a result, social-ecological vulnerability will vary across space at a range of scales, although we do not have a good understanding of the spatial patterns and drivers of this variability for Caribbean reefs [15,16].

We define social-ecological vulnerability to climate change as the degree to which a system, including both natural and human components, is susceptible to and unable to cope with the adverse effects of climate change, including climate variability and extremes [17–19]. The literature on assessing social-ecological vulnerability includes new approaches to operationalize theory with quantitative assessments and proposes improvements to the widely adopted Intergovernmental Panel on Climate Change (IPCC) vulnerability framework [14,20–24]. However, existing assessments focus on small spatial scales and/or examine impacts on single economic sectors (e.g. fisheries), with an emphasis on ecological resilience [25,26] or the vulnerability of local-scale communities [21,27–30]. Few studies have assessed social-ecological vulnerability over scales spanning multiple national jurisdictions (but see noteworthy exceptions [31,32]). Regional-scale assessments are required to address open questions about how vulnerability varies across countries within a region [33,34], which factors drive social-ecological vulnerability at the country scale, and whether national-level policies focused on changes to natural resource management or governance can modify vulnerability [18]. These questions are of particular relevance in the Caribbean, given expected spatial variation in vulnerability stemming from differing reef conditions and exposure to climate threats, as well as ongoing regional initiatives to mitigate and adapt to the impacts of climate change, such as the Caribbean Biodiversity Fund (https://www.caribbeanbiodiversityfund.org/) and the Caribbean Community's Climate Change Centre (https://www.caribbeanclimate.bz/). The many jurisdictions in the Caribbean also vary in their socio-economic characteristics and dependence on reef resources and take distinct approaches to conservation, marine resource management, and ocean governance [16].

**Figure 1.**
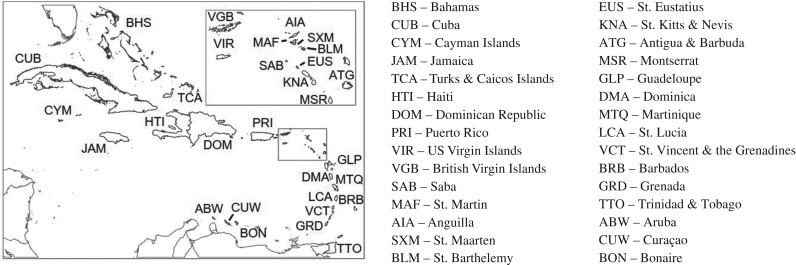
Study area, with island codes based on the ISO3 framework.

**Figure 2.**
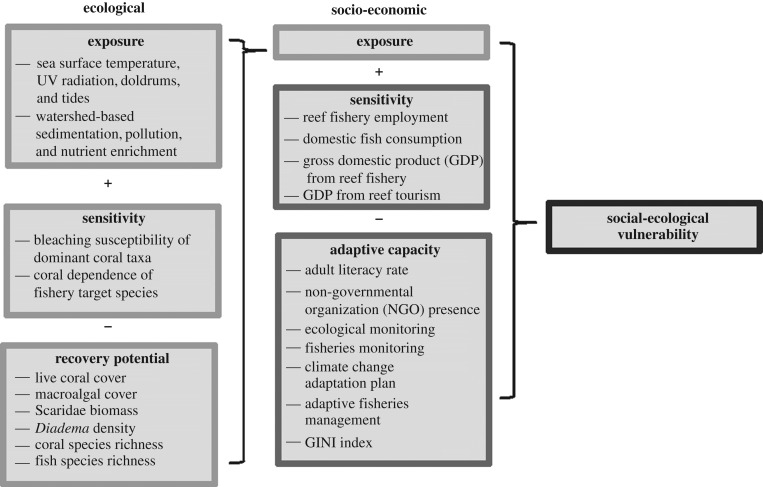
Components of social-ecological vulnerability, a function of the exposure that a system experiences, its sensitivity to exposure conditions and its ability to recover from or adapt to those conditions [17]. To operationalize social-ecological vulnerability to coral bleaching, we identified variables to estimate the main components of vulnerability (box headers) and combined them into a composite index. Adapted from Cinner *et al*. [28] and Marshall *et al*. [21]. The GINI index is a measure of income inequality.

We assess variability in the vulnerability of Caribbean coastal social-ecological systems to climate change-induced coral bleaching, a global phenomenon that disrupts the functioning of coral reef social-ecological systems. We focus on impacts to reef fisheries and marine tourism, comparing levels of ecological, socio-economic, and composite social-ecological vulnerability across the region at the island scale. Our assessment follows a modified IPCC framework, developing five quantitative indicators of vulnerability: (i) ecological exposure (to conditions that cause coral bleaching), (ii) ecological sensitivity (of the dominant coral taxa to bleaching conditions and of fished species to coral loss), (iii) ecological recovery potential of reef ecosystems, (iv) socio-economic sensitivity (based on economic dependence on reef fisheries and tourism), and (v) socio-economic adaptive capacity (defined as an island community's ability to adapt to environmental change or transform itself [18,35], assessed using proxies for livelihood diversification and learning, social organization, and the ability to detect and adapt to environmental change). We assess these indicators using existing data and scientific literature from 30 nations and territories (territories include overseas regions, departments, and collectivities associated with other sovereign nations) in the insular Caribbean (figure 1). We combine these five components into a composite index of vulnerability [21,28] (figure 2) and evaluate which components of the index most influence spatial patterns of vulnerability in the Caribbean. We then assess whether attributes commonly cited as potential mechanisms to mitigate climate vulnerability—specifically, governance characteristics, marine protected area (MPA) coverage, and fisheries management—explain variation in vulnerability to bleaching across the Caribbean. This research addresses important gaps in the literature including: assessing social-ecological vulnerability at larger spatial scales than most previous studies (e.g. the insular Caribbean); identifying policy-relevant governance and management factors that may influence island-scale vulnerability; and developing and compiling an unprecedented array of ecological and social indicator data for the Caribbean.

## 2. Results

### (a) Social-ecological vulnerability

**Figure 3.**
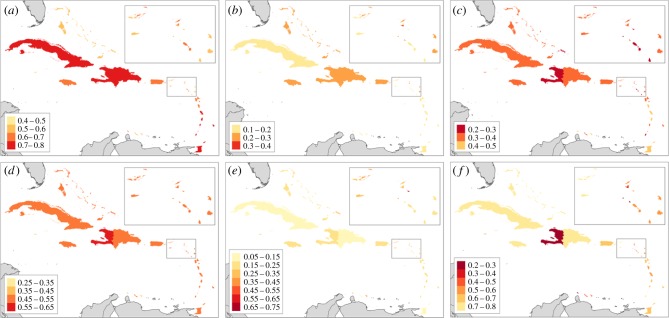
The range of values for each component of vulnerability: (*a*) ecological exposure, (*b*) ecological sensitivity, (*c*) ecological recovery potential, (*d*) socio-economic exposure, (*e*) socio-economic sensitivity, and (*f*) socio-economic adaptive capacity. Darker colours indicate increased vulnerability: for exposure and sensitivity (*a*,*b*,*d,* and *e*), darker colours indicate greater contributions to vulnerability, while darker colours indicate lower values of ecological recovery potential (*c*) and socio-economic adaptive capacity (*f*), meaning less mitigation of vulnerability. The bin sizes are the same across the six panels, but the ranges vary for the different components of vulnerability.

Here, we successfully apply a framework for assessing social-ecological vulnerability to a major climate change impact at the country or territory scale across many jurisdictions. Synthesizing island-level data on ecological and socio-economic indicators related to reef fisheries and marine tourism, we find considerable variability in the ecological and socio-economic components of vulnerability across the Caribbean (figure 3). With the exception of Haiti, the islands of the Greater Antilles and larger islands of the Lesser Antilles (e.g. Dominica, St. Lucia, and Grenada) have lower social-ecological vulnerability than other islands. Across the study area, there is low but significant spatial autocorrelation in ecological exposure and recovery potential, socio-economic adaptive capacity and composite social-ecological vulnerability (electronic supplementary material, table S20), with high and low values more spatially clustered than would be expected to occur randomly. Despite this geographical clustering, the components are measuring distinct aspects of vulnerability, as we found no significant correlations between the three ecological components or the three socio-economic components (electronic supplementary material, table S21). The spread in scores across islands is greatest for socio-economic sensitivity (coefficient of variation = 48), followed by ecological sensitivity (CV = 32), and socio-economic adaptive capacity (CV = 26; electronic supplementary material, table S22). Overall, there is greater spread across the islands in socio-economic than ecological variables (electronic supplementary material, table S23), thus driving more variation for the socio-economic compared to the ecological components of vulnerability.

**Figure 4.**
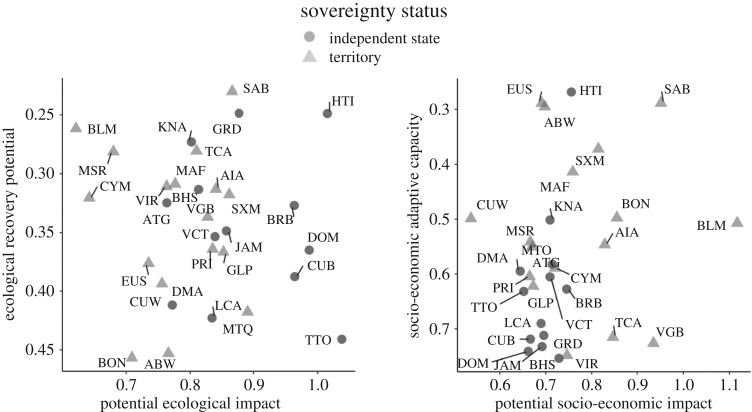
Potential ecological impacts (ecological exposure plus ecological sensitivity) versus recovery potential (*a*), and potential socio-economic impacts (i.e. socio-economic exposure and socio-economic sensitivity) versus adaptive capacity (*b*). High potential impacts indicate a system with high exposure and/or sensitivity to bleaching, while high recovery potential or adaptive capacity indicates that a system is able to respond to changes. Islands in the lower left quadrant have the lowest vulnerability (low potential impact and high recovery potential/adaptive capacity); islands in the upper right quadrant have the highest vulnerability. Sovereign islands are dark grey circles, while territories are light grey triangles.

Despite a common focus in the literature on exposure to bleaching conditions as the most important driver of vulnerability, islands' social-ecological vulnerability ranks are often inconsistent with their ecological exposure ranks (electronic supplementary material, table S24). Furthermore, some islands have fairly consistent rankings across the different components of vulnerability (e.g. St. Kitts and Nevis and Dominica), whereas others do not. For example, St. Barthelemy has low scores for ecological exposure and ecological sensitivity, but high socio-economic sensitivity and low ecological recovery potential and socio-economic adaptive capacity, leading to its ranking as among the most vulnerable islands overall (electronic supplementary material, table S24). Examining how potential ecological and socio-economic impact (exposure plus sensitivity) relate to ecological recovery potential and adaptive capacity, respectively (figure 4), we find that some islands occur in the same quadrant of both graphs (e.g. Haiti and Saba are highly vulnerable in ecological and socio-economic dimensions). Islands in the southern Caribbean (Aruba, Bonaire, Curaçao, and Trinidad and Tobago) have some of the highest scores for ecological recovery potential, while Haiti and the Dutch territories have among the lowest scores for adaptive capacity (electronic supplementary material, table S24). Aside from Haiti, the islands with the highest potential for socio-economic impact and the lowest adaptive capacity are all overseas territories (figure 4).

### (b) Covariates of vulnerability

Despite the many factors that could potentially influence social-ecological vulnerability at the island scale, we identify aspects of governance and resource management that are significant predictors of some components of vulnerability, explaining 47% of the variation in overall social-ecological vulnerability and 41% and 46% of the variation in socio-economic sensitivity and adaptive capacity, respectively (table 1). An island's fisheries regulation score (an index of the number of regulations in place that control fishing effort or reduce fishing pressure on key species or life-history stages) and its status as an independent state are negatively related to its social-ecological vulnerability, suggesting that fishing regulations and sovereignty reduce an island's overall vulnerability (*p* < 0.001). Socio-economic sensitivity is positively related to an island's mean Worldwide Governance Index (WGI) score, a measure of the quality of governance that includes levels of participation, stability, effectiveness, and corruption. Islands that have higher fisheries regulation scores have reduced socio-economic sensitivity and higher socio-economic adaptive capacity. Somewhat surprisingly, despite the frequent promotion of MPAs as a means of increasing resilience to climate impacts, we do not find the percentage of an island's coral reefs occurring within MPAs to be an important modifier of vulnerability, although it may have a small negative relationship with ecological sensitivity (*β*_C_ = −0.01, *p* = 0.1; table 1).

**Table 1.** Best-fit models relating the components of vulnerability to management and governance covariates. *β_C_* is the model coefficient for the percentage of coral reefs in MPAs, *β*_F_ is for the fisheries regulation score, *β*_W_ is for the Worldwide Governance Indicator score, and *β*_S_ is for island sovereignty; a positive coefficient indicates a positive relationship between independent sovereignty and the associated vulnerability component.

vulnerability component	model coefficient(s)	model fit
ecological exposure	*β*_W_ = −0.05, *β*_S_ = 0.08*	*R*^2^ = 0.37**
ecological sensitivity	*β*_C_ = −0.01	pseudo-*R*^2^ = 0.10
ecological recovery potential	*β*_F_ = −0.11	*R*^2^ = 0.01
socio-economic exposure	*β*_W_ = −0.13*, *β*_S_ = 0.09	pseudo-*R*^2^ = 0.38**
socio-economic sensitivity	*β*_F_ = −0.60; *β*_W_ = 0.30; *β*_S_ = −0.30	*R*^2^ = 0.41**
socio-economic adaptive capacity	*β*_F_ = 1.56**; *β*_S_ = 0.52*	*R*^2^ = 0.46**
social-ecological vulnerability	*β*_F_ = −0.49**; *β*_S_ = −0.17*	*R*^2^ = 0.47***

*p ≤ 0.05, **p ≤ 0.01, ***p ≤ 0.001.

WGI scores and sovereign status explain 37% and 38% of ecological exposure and socio-economic exposure, respectively: islands that are territories and those with higher WGI scores have lower exposure. We do not detect strong relationships between any of the covariates and ecological sensitivity or ecological recovery potential (*R*^2^ for best-fit models ≤ 0.1). We also ran the model for social-ecological vulnerability without the WGI as a covariate because the WGI is not available for all islands; that model produced similar results but explained less of the variation in social-ecological vulnerability (*R*^2^ = 0.30).

We find that islands that are independent nations are less vulnerable than overseas territories in several dimensions. Independent nations have significantly lower mean socio-economic sensitivity, higher socio-economic adaptive capacity, and lower overall social-ecological vulnerability to coral bleaching compared to territories (Kruskal–Wallis: mean_Independent_ = 0.07, mean_Territory_ = 0.26, *p* < 0.01), despite having greater ecological and socio-economic exposure (electronic supplementary material, table S26). Examining the underlying mechanism(s) that may be causing this result, independent islands rely on reef-based tourism for lower proportions of their economic productivity as measured by GDP (mean_Independent_ = 0.03, mean_Territory_ = 0.11, *p* < 0.01) and have less on-island consumption of domestically landed fish (mean_Independent_ = 0.88, mean_Territory_ = 0.93, *p* < 0.01; electronic supplementary material, table S26). Independent islands are also more likely to have fisheries monitoring programmes (*χ*^2^ = 6.8, *p* < 0.01) and climate change adaptation plans (*χ*^2^ = 11.9, *p* < 0.001). The difference in social-ecological vulnerability between independent islands and territories is highlighted by the finding that the 12 islands with the highest socio-economic sensitivity scores are all territories and eight out of the 10 lowest socio-economic adaptive capacity scores belong to territories (electronic supplementary material, table S24). Although territories tend to be smaller islands with lower human populations, these attributes do not explain the differences in social-ecological vulnerability. Island area and population size, when included as covariates, were not significant and did not improve the model (electronic supplementary material, table S27). However, island population explains 28% of the variation in watershed-based sedimentation, pollution, and nutrient enrichment, implying that the link between independent status and increased ecological exposure may relate to higher populations on independent islands (see the electronic supplementary material for details on these models).

## 3. Discussion

Our results provide the first regional quantitative analysis of social-ecological vulnerability to coral bleaching across many national jurisdictions, incorporating data from 30 island nations and territories. Previous studies of resilience and vulnerability to anthropogenic impacts have produced qualitative results [16,36] or have focused either on ecological aspects [12,25,26] or vulnerability and management of individual sectors [37–39]. We focused on the insular Caribbean, an area already struggling to cope with reef degradation but that has been poorly covered by recent global assessments due to data gaps for individual islands [31,40]. Crucially, we find that overseas territories have greater socio-economic sensitivity and lower adaptive capacity than independent islands, indicating that these territories, many of which have been excluded from previous assessments, may be at much greater risk to the socio-economic impacts of coral bleaching than previously appreciated. We found low correlation between the different components of vulnerability, consistent with an assessment focused on climate change impacts on fisheries [31] and indicating that high vulnerability can result from a range of factors. This low correlation has implications for management, as it indicates that the drivers of high vulnerability vary from island to island. The indicator-based framework we developed, as well as our findings relating sovereignty and environmental management to reduced vulnerability, are relevant to coral reef ecosystems around the world.

We found greater variation in socio-economic than ecological variables, implying that variation in overall social-ecological vulnerability across the Caribbean is in large part due to socio-economic differences. This is perhaps unsurprising, given the socio-economic diversity across Caribbean islands, including large differences in the economic importance of reef ecosystems and in governance characteristics. Of the socio-economic variables, the proportion of GDP from reef-based tourism had the greatest weighting in our equation (electronic supplementary material, table S25), but literacy rate, presence of ecological monitoring, and domestic consumption of local fish had greater average contributions to the islands' social-ecological vulnerability scores (electronic supplementary material, figure S1), due to the relatively high values of these variables.

While the ecological variables generally displayed less variation than the socio-economic variables, *Diadema antillarum* density showed a high level of variation, reflecting uneven recovery of urchin populations following a major disease epidemic and population crash in the early 1980s, with important implications for the suppression of algal overgrowth on reefs [41]. There was relatively low variation in the coral species richness and coral sensitivity score for each island, reflecting species homogeneity in the region [42] and low variation in taxonomic responses to bleaching [43]. Of the ecological variables, the environmental stress score had the largest contribution to the value of the overall social-ecological vulnerability (electronic supplementary material, table S25), but there was relatively little spread in the values of stress scores across the region (electronic supplementary material, table S23).

Our analysis of potential covariates of vulnerability indicates that independent islands (i.e. sovereign states) and islands with more fisheries regulations (e.g. catch limits, seasonal closures, species protections) have greater socio-economic adaptive capacity, due to a higher frequency of environmental monitoring and adaptive management. Fisheries regulations and MPAs have been promoted as management tools to enhance ecological resilience [44], but we did not find a strong relationship between the percentage of an island's reefs protected by MPAs or the number of fisheries regulations and either ecological sensitivity or ecological recovery potential. The MPA result may in part reflect the different levels of use restrictions and management effectiveness in MPAs: our dataset included all types of MPAs (including areas that allow some fishing activity), and enforcement and management effectiveness and associated ecological outcomes vary widely in the region [45]. MPAs may still play an important role in reef recovery, resilience, and conservation in locations where they have adequate management regulations and capacity. The lack of impact of fisheries regulations on ecological vulnerability may similarly reflect barriers to effective enforcement of restrictions in the region [46]. Our analysis of MPA impacts may also be complicated by biogeographic patterns, as well as historic and ongoing ecological degradation on Caribbean reefs; Swain *et al*. [43] found that the tropical Atlantic had relatively low taxonomic sensitivity to coral bleaching, and the dominance of coral taxa with low sensitivity may reflect die-offs of more sensitive species during previous bleaching events. Protected reefs may thus have higher abundances of more sensitive coral species because their protected status reduces localized stressors; the dominance of more sensitive species in protected areas may then lead to greater ecological sensitivity to coral bleaching [47].

Although territorial governments, and in some cases, the dependent territories themselves, may view overseas territories as benefitting in some ways from a colonial relationship, we found that islands that are overseas territories had greater socio-economic sensitivity to coral bleaching, resulting from higher economic dependence on reef-based tourism and a greater contribution of locally landed fish to domestic seafood consumption. The higher dependence on tourism may reflect development priorities of overseas governments and the propensity of tourists to visit their country's territories. Furthermore, at least some of the increase in the percentage of fishery landings that are consumed on-island may also be related to increased dependence on tourism, as tourists can drive demand for seafood [48]. The greater economic reliance on reef-based tourism in territories implies less diverse economies and greater economic losses if climate change-related coral bleaching, sea-level rise, and increased storm intensity reduce tourism [49]. Sample sizes are insufficient to determine whether the territories of different countries have significantly different patterns of vulnerability. However, a visual assessment of the results suggests that Dutch territories appear to have lower socio-economic adaptive capacity than the other territories (electronic supplementary material, figure S2).

We produced an unprecedented compilation of socio-economic and ecological data across 30 Caribbean islands. The analytical framework we have developed can be extended to other locations or updated easily if new data are collected in the region, and our results reveal data gaps that could be filled to improve regional vulnerability assessments. For example, we see opportunities to improve assessments of socio-economic sensitivity and adaptive capacity through the collection of standardized data; unemployment and poverty rates may be important indicators of socio-economic vulnerability, but there are no systematic surveys of these variables across the Caribbean region and island-level surveys use different thresholds and definitions. Furthermore, data on fishing activity may be unreliable, as substantial activity in the artisanal and subsistence sectors is unreported [50]. Another important limitation of our assessment of social-ecological vulnerability is the lack of data on larval connectivity, recruitment, and post-settlement survival in our index of ecological recovery potential. While the dispersal and recruitment of coral and reef fish larvae is a significant factor in the recovery of coral reef ecosystems [44], the stochasticity of larval connectivity [51] and the behavioural, ecological, and oceanographic data required for accurate models of dispersal and recruitment patterns limit our ability to incorporate these factors [52].

We combined diverse indicators to develop measures of ecological and socio-economic exposure, sensitivity, recovery potential, and adaptive capacity, and subsequently combined these components of vulnerability into an index of social-ecological vulnerability. Indicator selection and methods of aggregation can influence the final vulnerability scores, but we attempted to use indicators that could be assessed consistently across the study region and to ground our aggregation decisions in established vulnerability frameworks. Furthermore, in the absence of expert or stakeholder input on the relative importance of the different indicators or components in the specific context of Caribbean coral reef social-ecological systems, we chose to use equal weightings [53], but these weightings could be modified if more information were available. We also did not include all economic sectors or ecosystem services that could be impacted by coral bleaching or climate change, such as shoreline protection, in our analysis [6]. Future vulnerability assessments for the Caribbean that address these assumptions and limitations merit attention.

The relative values of each component of vulnerability (and their underlying variables) can guide managers and policymakers in their efforts to reduce vulnerability: although large-scale vulnerability assessments should be complemented with more localized assessments with stakeholder participation to better target local policy interventions [30,54], they still reveal important broad-scale patterns (e.g. figure 4). Islands with high potential for ecological impacts and low recovery potential, like Haiti and Saba, could use management measures to reduce their ecological exposure and sensitivity (e.g. by improving land use planning and agricultural practices to decrease erosion and nutrient pollution, using less sensitive coral taxa in reef restoration efforts and shifting fishing effort to less reef-dependent species) and/or to increase their ecological recovery potential (e.g. by reducing land-based pollution that promotes macroalgal growth; by protecting important components of the ecosystem currently subject to fishing through appropriate fisheries management; and by using reef restoration or artificial reefs with high architectural complexity to increase coral recruitment and densities of parrotfish and other herbivores [55]). Islands with low potential ecological impacts and low recovery potential, like Montserrat and St. Barthelemy, could focus their efforts on maintaining low exposure and sensitivity while enhancing ecological recovery potential. Islands with high potential socio-economic impacts and low adaptive capacity (and thus high overall social-ecological vulnerability), such as Saba, could use policy measures to increase adaptive capacity (e.g. by increasing literacy levels and decreasing income inequality, implementing adaptive environmental management, and establishing ecological and fisheries monitoring) and/or decrease socio-economic sensitivity through economic diversification. While some factors contributing to ecological exposure are impossible to manage at the island level (e.g. sea surface temperature and solar radiation), all islands could reduce their risk of experiencing bleaching conditions by managing local water quality and other land-based impacts to reefs [56].

A key advance of our approach is assessing vulnerability at the national scale across many jurisdictions. Although this scale may mask important local-scale and socially differentiated variation in vulnerability, the island-level estimates of vulnerability are policy-relevant and actionable. Our findings regarding the importance of socio-economic factors such as ecological monitoring and adaptive environmental management suggest responses at the island level. Furthermore, our research complements and contextualizes site-based research [57,58], which can identify more focused, local-scale interventions within an island-wide framework. Finally, this scale of analysis enabled important insights that could not have been identified from local-scale analyses. Specifically, we determined that different components of vulnerability are uncorrelated at a national scale across a diverse region like the Caribbean; that socio-economic factors can be more important than ecological factors, including exposure to climate threats, in driving variation in climate vulnerability; and that independent nations may be more robust to climate change than dependent territories. These insights contribute to our understanding of climate change vulnerability across multiple dimensions and can inform policy interventions to reduce vulnerability to coral bleaching.

## 4. Material and methods

### (a) Study area

We compiled ecological, socio-economic, and governance data from 30 island nations and territories (hereafter ‘islands’) in the Caribbean (figure 1). Islands formed our unit of study, rather than sites or communities, to reflect the resolution at which comparable socio-economic and governance data are typically available, as well as the scale at which many resource management decisions are made [31].

### (b) Vulnerability framework

The IPCC has historically defined vulnerability as a function of exposure, sensitivity, and adaptive capacity [17]. This definition was revised in 2012 to categorize exposure as a factor distinct from vulnerability that contributes to the risk of experiencing impacts from climate change [59]; here, we include exposure as a factor contributing to vulnerability to make our framework and results comparable with past assessments of other relevant aspects of climate vulnerability and to reflect the potential for significant differences in exposure to the biophysical conditions that can cause bleaching events throughout the Caribbean region. We operationalized this definition to estimate socio-economic and ecological vulnerability, drawing from existing frameworks [28,31,40] (figure 2). We selected indicators for the components of vulnerability based on scientific theory and evidence, bounded by the availability of recent, comparable data across all islands, as described in the electronic supplementary material. We also examined how different aspects of marine resource management might explain variation in social-ecological vulnerability. For additional information on indicators, data sources, assumptions, methods, and supporting references, refer to the electronic supplementary material.

### (c) Components of ecological vulnerability

#### (i) Ecological exposure

Variation in environmental factors drives spatial variation in exposure to bleaching conditions. We calculated the exposure of each island's coral reefs using a spatial model of environmental conditions that may trigger (e.g. sea surface temperature, solar radiation) and mitigate (e.g. tidal amplitude) bleaching [60]. To account for the role of sedimentation and land-based pollution and nutrient enrichment in increasing the incidence of bleaching, we calculated the average relative risk of water quality issues for the reefs of each island and used this value as a multiplier (ranging from 1 for islands with very low exposure to watershed-based threats to 1.4 for islands with high exposure to this threat) to modify the ecological exposure score [61] (electronic supplementary material, table S1).

#### (ii) Ecological sensitivity

We assessed two indicators: first, we estimated the sensitivity of the dominant hard coral species to bleaching to assess ecosystem sensitivity based on the key habitat-forming species. We identified the most abundant coral taxa in each island from reef surveys that occurred after the region-wide 2005 bleaching event [4] and used a taxon-specific bleaching response index (BRI) [43] to calculate the island-level coral sensitivity score as the average of the BRIs from the most abundant coral taxa, with BRI scores ranging from 0 to 1 (higher values represent higher bleaching impacts) (electronic supplementary material, table S2). Second, we estimated the sensitivity to bleaching impacts of the fish and invertebrate species targeted in each island's fisheries to assess the ecological components most directly valued by local communities. We identified the taxa that accounted for at least 10% of cumulative landings from 2005 to 2014, calculated a sensitivity index for each taxon based on habitat usage and adult home range size (electronic supplementary material, table S3), summed each target taxon sensitivity score after it was multiplied by its proportion of the catch (electronic supplementary material, table S4) and then inverted the scores so that higher values indicated greater sensitivity. We averaged island scores for coral and target species sensitivity into a composite index of ecological sensitivity.

#### (iii) Ecological recovery potential

We assumed that reefs that are less degraded will be more likely to recover following bleaching events than reefs that are more highly degraded [44]. We compiled data on six indicators of reef health: live coral cover, macroalgal cover, Scaridae (parrotfish) biomass, *Diadema antillarum* (sea urchin) density, and the species richness of reef fish and hard corals. The data on coral and macroalgal cover, Scaridae biomass, and *Diadema* density came from reef survey data in published papers, grey literature, and government- and volunteer-monitoring programmes, and we filled data gaps using ecoregional averages (electronic supplementary material, table S5). We scaled Scaridae biomass values by dividing by a representative unfished biomass value for the region of 47 g m^−2^ [39], and capped scaled values at 1.0. We scaled *Diadema* densities by dividing the values by a regional historical baseline density of 7.7 individuals m^−2^ [62].

We assembled data on coral species richness from recent surveys, using ecoregional averages to fill in gaps (electronic supplementary material, table S6). We used reef fish species richness data reported in a biogeographic analysis and used the derived linear relationship between island area and reef fish species richness to fill gaps (electronic supplementary material, equation (S4) and table S7). We rescaled the values of coral and reef fish species richness by dividing each island's value by the total number of species in each category found across the 30 islands. For the composite index of ecological recovery potential, we inverted algal cover values and then averaged the six variables for each island.

We combined our indices of ecological exposure (EE), sensitivity (ES), and recovery potential (ERP) into an index of ecological vulnerability (EV):

4.1 EV=EE+ES−ERP.  

### (d) Components of socio-economic vulnerability

#### (i) Socio-economic exposure

The composite index of ecological vulnerability, rescaled to bound the values from 0 to 1 (i.e. (*EV* + 1)/3), represents socio-economic exposure to coral bleaching, because ecological vulnerability describes the potential for bleaching conditions to cause ecological changes that can in turn affect human communities [28].

#### (ii) Socio-economic sensitivity

We estimated socio-economic sensitivity to coral bleaching as an island's dependence on reef fisheries and reef-based tourism. We considered the role of reef fisheries in local employment (i.e. Pop_SSF_ = proportion of each island's population engaged in small-scale fishing; electronic supplementary material, table S8), local diets (i.e. LF = percentage of each island's seafood landings consumed domestically based on production and export data; electronic supplementary material, table S9), and overall island-level economic productivity (i.e. GDP*_F_* = percentage of the island's gross domestic product (GDP) that comprised reef fishery value in 2010; electronic supplementary material, table S10). We scaled GDP*_F_* by the maximum contribution of reef fisheries to a national GDP in 2010 (13.5% [63]). We also estimated the proportion of the island's annual GDP that came from reef-based tourism (i.e. GDP_T_; electronic supplementary material, table S11, scaled by the maximum contribution of reef tourism to a nation's GDP in a recent global assessment (43.19% [64]).

We developed a composite index of socio-economic sensitivity (SS):

4.2 $$\mathrm{SS}=0.5\times \mathrm{GD}P_{T}+0.5\times \frac{\mathrm{Po}p_{\mathrm{SSF}}+\mathrm{LF}+\mathrm{GD}P_{F}}{3}.\;$$

#### (iii) Socio-economic adaptive capacity

We assessed adaptive capacity, i.e. society's ability to predict and respond to changes by minimizing negative consequences, and capitalizing on emerging opportunities [35], using four components. First, we used adult literacy rate (Lit) to indicate the capacity for learning on the individual level and the potential for livelihood diversification at the island level (electronic supplementary material, table S12). Second, we used the presence of international non-governmental organizations (NGO) that focus on marine conservation to indicate social capacity at the island level (electronic supplementary material, table S13). Third, we determined whether each island had ecological (*M*_E_) and fisheries (*M*_F_) monitoring programmes, run by government agencies, NGOs, or academic groups (electronic supplementary material, table S14), assuming that such programmes allow for earlier and more accurate detection of environmental change, facilitating adaptation. Lastly, we used two indicators of the ability to respond to environmental change: (i) evidence of past adaptive environmental management as indicated by climate change plans (Plan_CC_) and adaptive fisheries management plans (Plan_AF_; electronic supplementary material, table S15) and (ii) economic inequality measured using the inverted GINI index (GINI; electronic supplementary material, table S16), assuming islands with higher economic parity would be better able to withstand and adapt to environmental and economic shocks.

We combined the seven variables across four subcomponents into a composite index of socio-economic adaptive capacity:

4.3 $$\mathrm{SAC}=0.25\times \mathrm{Lit}+0.25\times \mathrm{NGO}+0.25\times \frac{M_{E}+M_{F}}{2}+0.25\times \frac{\mathrm{Pla}n_{\mathrm{CC}}+\;\mathrm{Pla}n_{\mathrm{AF}}+\mathrm{GINI}}{3}.$$

### (e) Social-ecological vulnerability

We calculated a composite social-ecological vulnerability score for each island as the sum of socio-economic exposure (equivalent to ecological vulnerability, rescaled to range from 0 to 1) and socio-economic sensitivity, minus socio-economic adaptive capacity. The sum of socio-economic exposure and socio-economic sensitivity represents the potential socio-economic impact. Scores for each component of ecological and socio-economic vulnerability could range from 0 to 1, and thus social-ecological vulnerability could range from −1 to 2. We ranked the islands by each component of social-ecological vulnerability. We also tested for spatial autocorrelation in each component of vulnerability using Moran's *I* test on centroids of each island.

### (f) Governance and management covariates

We examined four covariates describing marine resource management and governance that might explain variation in social-ecological vulnerability to coral bleaching. MPAs and fisheries regulations have been promoted as tools to increase ecological recovery potential and reduce exposure to local stressors [44,65], although the empirical evidence is not conclusive [66]. General governance characteristics of an island, such as political stability, may influence socio-economic adaptive capacity and socio-economic sensitivity [67]. We assessed MPA coverage (as a percentage of each island's coral reef area; electronic supplementary material, table S17); fisheries regulations that control fishing effort or reduce fishing pressure on key species and/or life-history stages (electronic supplementary material, table S18); the World Bank's Worldwide Governance Indicator (WGI) score that reflects six dimensions of governance: voice and accountability, political stability and the absence of violence, government effectiveness, regulatory quality, rule of law, and control of corruption [68] (electronic supplementary material, table S19); and the sovereign status of each island (independent nation versus overseas territory, electronic supplementary material, table S19). Some of these covariates (e.g. WGI scores) have been used as indicators of adaptive capacity [24], but we chose to use more specific indicators with clear hypothesized or empirical links to adaptive capacity (see electronic supplementary material for explanations of each indicator chosen); our covariates represent variables that have been suggested by previously published research to be potentially important factors in explaining vulnerability more generally.

We developed generalized linear models (GLMs) for all components of vulnerability, modelling each component as a function of the four covariates. Model selection was performed in R [69] in stepwise fashion. We used the Akaike information criterion, squared Pearson's correlation coefficient (*R*^2^), and unadjusted squared deviance (*D*^2^) to select the most parsimonious and best-fit model. We also used Kruskal–Wallis tests to assess differences in the components of vulnerability, their constituent variables, and the covariates based on sovereign status while accounting for non-normal data distributions.

## Supplementary Material

Supplementary Methods and Data

## Data accessibility

This article has no additional data.
