# Supplementary material for: Sovereign states in the Caribbean have lower social-ecological vulnerability to coral bleaching than overseas territories
Source: Proc Biol Sci. 2019 Feb 20;286(1897):20182365. doi: 10.1098/rspb.2018.2365 (PMC6408901; doi:10.1098/rspb.2018.2365)
Supplement: Supplementary Methods and Data [file rspb20182365supp1.docx]

Supplementary Information for *Sovereign states in the* *Caribbean have lower social-ecological vulnerability to coral bleaching than overseas territories*

**Table of Contents**

[I. Indices of Vulnerability 1](#_Toc534644610)

[A. Components of Ecological Vulnerability 2](#_Toc534644611)

[Ecoregional Gap Filling 2](#_Toc534644612)

[Ecological Exposure 2](#_Toc534644613)

[Ecological Sensitivity 5](#_Toc534644614)

[Ecological Recovery Potential 18](#_Toc534644615)

[B. Components of Socioeconomic Vulnerability 27](#_Toc534644616)

[Socioeconomic Exposure 27](#_Toc534644617)

[Socioeconomic Sensitivity 27](#_Toc534644618)

[Socioeconomic Adaptive Capacity 40](#_Toc534644619)

[C. Covariates 52](#_Toc534644620)

[Marine Protected Area (MPA) Coverage 52](#_Toc534644621)

[Fisheries Regulations 53](#_Toc534644622)

[Governance Characteristics 58](#_Toc534644623)

[II. Analyses 61](#_Toc534644624)

[A. Spatial Autocorrelation 61](#_Toc534644625)

[B. Correlations in Components of Vulnerability 62](#_Toc534644626)

[C. Coefficients of Variation 62](#_Toc534644627)

[D. Vulnerability Rankings 64](#_Toc534644628)

[E. Contribution of Variables to Vulnerability 65](#_Toc534644629)

[F. Differences between Territories of Different Countries 67](#_Toc534644630)

[G. Model Selection 68](#_Toc534644631)

[References 74](#_Toc534644632)

# I. Indices of Vulnerability

Our framework for calculating social-ecological vulnerability draws from the Intergovernmental Panel on Climate Change and other published vulnerability frameworks, defining social-ecological vulnerability (*SEV*) as a function of ecological exposure (*EE*), ecological sensitivity (*ES*), ecological recovery potential (*ERP*), socioeconomic sensitivity (*SS*), and socioeconomic adaptive capacity (*SAC*) (1–4):

$SEV=\frac{\left( EE+ES-ERP \right)+1}{3}+SE-SAC$ $(S1)$

## A. Components of Ecological Vulnerability

### Ecoregional Gap Filling

When we were unable to locate island-level data for ecological variables or estimate their values using empirical relationships with island size or other relevant variables, we filled the data gaps using ecoregional averages. We used Spalding *et al*.’s (2007) classification of the Caribbean into four ecoregions (the Bahamanian, Eastern Caribbean, Greater Antilles, and Southern Caribbean) to assign each island to an ecoregion (see Table S2 for a list of the islands in each ecoregion). We calculated the ecoregional average as the mean value for the variable in question for the islands within that ecoregion. Where islands did not have any data for the variable in question, we then assigned them the value of the corresponding ecoregional average.

### Ecological Exposure

We calculated the average exposure to environmental conditions that can cause bleaching for each island using a spatially-explicit environmental stress model developed by Maina *et al*. (5); the model integrates radiation stress factors (sea surface temperature and solar radiation), stress reinforcing factors (chlorophyll and suspended solids), and stress reducing variables (doldrums and tidal ranges) related to coral bleaching. We estimated the mean stress score for each island by overlaying a shapefile of stress scores from the Maina *et al*. (2011) model with a shapefile of Caribbean coral reefs (6). We assigned reef polygons to the Exclusive Economic Zones (EEZs) of the 30 island nations/territories using a shapefile of EEZs (7). Within the reefs assigned to each island’s EEZ, we calculated the mean score from the Maina *et al*. (2011) layer using ArcGIS (8) (Table S1). Maina *et al*. calculated stress scores for all oceans globally, but we did not rescale the mean stress scores so that the values for Caribbean islands would range from 0-1 because we wanted our framework to be applicable to additional islands and countries and we did not want our methods to imply that the most or least exposed island could not become any more or less exposed.

Sedimentation, pollution, and nutrient enrichment from land-based sources increase the prevalence of coral bleaching. Sedimentation can cause a reduction in the concentration of zooxanthellae and bleaching of colonies that are in prolonged contact with sediment (9), while nutrient pollution reduces some Caribbean coral species’ thermal tolerances, thus reducing their resistance to bleaching (10,11). To account for the role of sedimentation, pollution, and nutrient enrichment in causing bleaching and mediating the effects of other environmental stressors that can cause bleaching, we combined the scores from the Maina et al. (2011) model with a measure of the watershed-based risk of sedimentation, pollution, and nutrient enrichment, using a model from the World Resources Institute’s *Reefs at Risk Revisited* (12,13). The model estimates the threats to reefs from land-based pollutants discharged by watersheds into coastal waters using data on slope, land cover type, precipitation, and soil type to estimate the relative erosion rate for each watershed, then estimating the amount of sediment delivered at each river mouth based on watershed size and the presence of mangroves and dams. The model estimates each reef’s exposure to the ensuing sediment plume based on a linear decay rate from the mouth of the river. We used the GIS data provided along with the *Reefs at Risk Revisited* report (13) to calculate a watershed-threat multiplier for each island based on the average level of exposure of that island’s reefs. The data included all reef units coded as having “Low,” “Medium,” or “High” risk, which the report’s authors quantified as scores of 0, 1, and 2, respectively. To convert this into a multiplier for use with the Maina et al. (2011) stress scores, we coded low risk reefs as “1,” medium risk reefs as “1.2,” and high risk reefs as “1.4,” then calculated the mean risk level for the reefs of each island using an average weighted by the total area of reef at each level of risk. The ultimate value of each island’s ecological exposure is equal to the exposure score (from Maina et al.) multiplied by the watershed-based pollution multiplier.

**Table S1.** Ecological exposure values from Maina *et al*.’s (2011) model and watershed-based pollution multipliers from *Reefs at Risk Revisited*.

| Island | Exposure value | Watershed-based pollution multiplier |
| --- | --- | --- |
| Anguilla | 0.61 | 1.00 |
| Antigua & Barbuda | 0.53 | 1.02 |
| Aruba | 0.62 | 1.01 |
| Bahamas | 0.54 | 1.00 |
| Barbados | 0.66 | 1.18 |
| Bonaire | 0.57 | 1.00 |
| British Virgin Islands | 0.62 | 1.01 |
| Cayman Islands | 0.51 | 1.00 |
| Cuba | 0.73 | 1.06 |
| Curaçao | 0.56 | 1.04 |
| Dominica | 0.57 | 1.18 |
| Dominican Republic | 0.58 | 1.25 |
| Grenada | 0.72 | 1.05 |
| Guadeloupe | 0.60 | 1.09 |
| Haiti | 0.59 | 1.34 |
| Jamaica | 0.52 | 1.19 |
| Martinique | 0.64 | 1.19 |
| Montserrat | 0.48 | 1.15 |
| Puerto Rico | 0.55 | 1.14 |
| Saba | 0.60 | 1.00 |
| St. Barthelemy | 0.51 | 1.00 |
| St. Eustatius | 0.47 | 1.00 |
| St. Kitts & Nevis | 0.53 | 1.21 |
| St. Lucia | 0.61 | 1.18 |
| St. Maarten | 0.54 | 1.00 |
| St. Martin | 0.64 | 1.04 |
| St. Vincent & the Grenadines | 0.71 | 1.00 |
| Trinidad & Tobago | 0.80 | 1.10 |
| Turks & Caicos | 0.58 | 1.00 |
| US Virgin Islands | 0.57 | 1.03 |

### Ecological Sensitivity

We combined indicators of the sensitivity of coral species (*S_C_*) and of fishery target species (*S_TS_*) into a single index of ecological sensitivity using equation (S2):

$ES=\frac{S_{C}+ S_{TS}}{2}$ (S2)

Coral sensitivity: There is considerable taxonomic variation in the susceptibility of coral taxa to bleaching when exposed to the same environmental conditions (14–18). This variation arises from differences in life history traits (19), in the species of *Symbiodinium* (symbiotic dinoflagellates) present (20), and other physiological factors (18). We assumed that islands where the most abundant coral species tended to be more sensitive to bleaching would experience greater sensitivity to bleaching conditions. To estimate the bleaching sensitivity of the dominant coral taxa, we identified the most abundant coral taxa in each island (by number of colonies or percent cover) from reef surveys. We only considered studies and surveys reporting data collected since the summer of 2005, to capture changes in coral community composition following the Caribbean-wide 2005 bleaching event (21). We made an exception to this rule for Curaçao, which did not experience mortality from the 2005 bleaching event (22). We compiled the top three coral taxa from these surveys (for some islands, more than three top coral taxa were included; this occurred when multiple taxa were ranked as the third most abundant species). For most islands, we found reports of the most abundant species, but in some cases, the most abundant taxa were reported at the genus or complex level, as in the case of *Agaricia* spp. For Saba and the Dominican Republic, we only found information on the top two most abundant coral taxa (23,24); we used gap-filling methods to calculate the coral sensitivity for these islands (see paragraph on gap filling below).

To quantify the sensitivity of the most abundant coral taxa, we used the taxon-specific bleaching response index (BRI) developed by Swain *et al*. (2016) (18). BRI scores, which are based on over two decades of bleaching and mortality records around the world, represent the percentage of tissue area affected by bleaching across different sites and bleaching events. BRI scores range from 0-1 globally, with higher values representing higher levels of bleaching and related mortality. While the dataset underlying the BRI has limitations, including coral taxa with low sample sizes, it represents the most comprehensive and standardized method for comparing coral sensitivity to bleaching. The BRI scores capture the range of taxon sensitivity globally; to allow for future extensions of our framework beyond the thirty islands assessed in this study (or for taxonomic changes in coral composition in the Caribbean), we did not rescale the BRI scores to range from 0-1 in the Caribbean.

rescale the mean stress scores so that the values for Caribbean islands would range from 0-1 because we wanted our framework to be applicable to additional islands and countries and we did not want our methods to imply that the most or least exposed island could not become any more or less exposed.

We extracted the BRI score for each taxon included in our list of the most abundant taxa per island from Swain *et al*.’s Table S4 (18). For taxa reported at the genus or complex level, we took the average from the species belonging to that category: the BRI calculated for *Agaricia* spp. was the average of all Caribbean species of *Agaricia* included in the Swain *et al*. dataset (using geographic ranges reported by the IUCN Red List (25)), and the *Montastraea annularis* complex was the average of the BRIs for *Orbicella annularis*, *O. faveolata*, and *O. franksi* (26). We calculated each island’s sensitivity score as the average of the BRI scores for the top coral taxa on that island (Table S2).

We were unable to locate recent reports of the most abundant coral taxa for Guadeloupe, St. Martin, St. Barthelemy, Haiti, Aruba, St. Lucia, the British Virgin Islands, St. Maarten, and St. Vincent & the Grenadines; as previously reported, we also were only able to locate the top two most abundant taxa in Saba and the Dominican Republic. For these eleven islands, we used the average coral sensitivity score for islands in their ecoregions to estimate their coral sensitivity scores (27), as detailed in the “Ecoregional Gap Filling” section (Table S2).

**Table S2.** Coral sensitivity scores. The average score for each ecoregion is provided in parentheses under the name of the ecoregion. The information in brackets after each data source lists additional information on the location and size of the survey sample.

| Ecoregion *(Ecoregional score)* | Island | Coral taxon | Taxon BRI | Island Score | Source |
| --- | --- | --- | --- | --- | --- |
| Bahamanian *(0.2070)* | Bahamas | *Agaricia agaricites* | 0.3351 | 0. 2259 | Deleveaux et al. 2013  [23 reefs] |
|  |  | *Orbicella annularis* | 0.1889 |  |  |
|  |  | *Orbicella faveolata* | 0.2196 |  |  |
|  |  | *Porites astreoides* | 0.1601 |  |  |
|  | Turks & Caicos | *Agaricia* spp. | 0.1642 | 0.1880 | Dikou et al. 2009 [South Caicos] |
|  |  | *Orbicella annularis* | 0.1889 |  |  |
|  |  | *Siderastrea* spp. | 0.2109 |  |  |
| Eastern Caribbean *(0.1676)* | Anguilla | *Agaricia humilis* | 0.1100 | 0.1535 | K. Gumbs 2012 |
|  |  | *Porites astreoides* | 0.1601 |  |  |
|  |  | *Siderastrea siderea* | 0.1905 |  |  |
|  | Antigua & Barbuda | *Porites astreoides* | 0.1601 | 0.1829 | Ruttenberg et al. 2013 [Barbuda] |
|  |  | *Porites porites* | 0.1982 |  |  |
|  |  | *Siderastrea siderea* | 0.1905 |  |  |
|  | Barbados | *Agaricia* spp. | 0.1642 | 0.1686 | Oxenford et al. 2008 |
|  |  | *Montastraea* spp. | 0.1816 |  |  |
|  |  | *Porites astreoides* | 0.1601 |  |  |
|  | British Virgin Islands | - | - | 0.1676 | Ecoregional average |
|  | Dominica | *Porites astreoides* | 0.1601 | 0.1715 | Steiner & Kerr 2008 [Table 2] |
|  |  | *Meandrina meandrites* | 0.1639 |  |  |
|  |  | *Siderastrea siderea* | 0.1905 |  |  |
|  | Grenada | *Orbicella annularis* | 0.1889 | 0.1824 | Bouchon et al. 2008 |
|  |  | *Porites astreoides* | 0.1601 |  |  |
|  |  | *Porites porites* | 0.1982 |  |  |
|  | Guadeloupe | - | - | 0.1676 | Ecoregional average |
|  | Martinique | *Orbicella annularis* | 0.1889 | 0.1895 | Cowan 2006 [Table 3] |
|  |  | *Orbicella faveolata* | 0.2196 |  |  |
|  |  | *Porites astreoides* | 0.1601 |  |  |
|  | Montserrat | *Porites astreoides* | 0.1601 | 0.1172 | Wild et al. 2007 [Tables 3-5] |
|  |  | *Madracis formosa* | 0.0275 |  |  |
|  |  | *Meandrina meandrites* | 0.1639 |  |  |
|  | Saba | *-* | - | 0.1676 | Ecoregional average |
|  | Saint Barthelemy | - | - | 0.1676 | Ecoregional average |
|  | Saint Martin | - | - | 0.1676 | Ecoregional average |
|  | Sint Eustatius | *Meandrina meandrites* | 0.1639 | 0.1662 | Debrot et al. 2014  [Table 4] |
|  |  | *Montastraea cavernosa* | 0.1442 |  |  |
|  |  | *Siderastrea siderea* | 0.1905 |  |  |
|  | Sint Maarten | - | - | 0.1676 | Ecoregional average |
|  | St. Kitts & Nevis | *Orbicella faveolata* | 0.2196 | 0.1901 | Bruckner & Williams 2012 [Figure 11] |
|  |  | *Porites astreoides* | 0.1601 |  |  |
|  |  | *Siderastrea siderea* | 0.1905 |  |  |
|  | St. Lucia | - | - | 0.1676 | Ecoregional average |
|  | St. Vincent & the Grenadines | - | - | 0.1676 | Ecoregional average |
|  | US Virgin Islands | *Diploria* spp. | 0.1578 | 0.1544 | Rothenberger et al. 2008  [Figure 2.24] |
|  |  | *Montastraea* spp. | 0.1816 |  |  |
|  |  | *Porites* spp. | 0.1238 |  |  |
| Greater Antilles *(0.2004)* | Cayman Islands | *Agaricia* spp. | 0.1642 | 0.1679 | Jones et al. 2008 |
|  |  | *Diploria* spp. | 0.1578 |  |  |
|  |  | *Montastraea* spp. | 0.1816 |  |  |
|  | Cuba | *Acropora palmata* | 0.2582 | 0.2402 | Alcolado 2013  [6 sites];  Perera-Valderrama et al. 2016  [2 sites] |
|  |  | *Porites astreoides* | 0.1601 |  |  |
|  |  | *Porites furcata* | 0.3022 |  |  |
|  | Dominican Republic | - | - | 0. 2004 | Ecoregional average |
|  | Haiti | - | - | 0. 2004 | Ecoregional average |
|  | Jamaica | *Agaricia agaricites* | 0.3351 | 0.2286 | Bruckner et al. 2014  [Pedro Bank] |
|  |  | *Porites astreoides* | 0.1601 |  |  |
|  |  | *Siderastrea siderea* | 0.1905 |  |  |
|  | Puerto Rico | *Montastraea cavernosa* | 0.1442 | 0.1649 | NCCOS 2016 |
|  |  | *Porites astreoides* | 0.1601 |  |  |
|  |  | *Siderastrea siderea* | 0.1905 |  |  |
| Southern Caribbean *(0.2091)* | Aruba | - | - | 0.2091 | Ecoregional average |
|  | Bonaire | *Montastraea cavernosa* | 0.1442 | 0.1842 | Steneck et al. 2015 |
|  |  | *Orbicella annularis* | 0.1889 |  |  |
|  |  | *Orbicella faveolata* | 0.2196 |  |  |
|  | Curaçao | *Agaricia agaricites* | 0.3351 | 0.2479 | Bouchon et al. 2008 |
|  |  | *Orbicella annularis* | 0.1889 |  |  |
|  |  | *Orbicella faveolata* | 0.2196 |  |  |
|  | Trinidad & Tobago | *Diploria strigosa* | 0.1757 | 0.1953 | Mallela et al. 2010 [Tobago] |
|  |  | *Orbicella faveolata* | 0.2196 |  |  |
|  |  | *Siderastrea siderea* | 0.1905 |  |  |

Target species sensitivity: Coral bleaching has disparate impacts across fish and macroinvertebrate taxa; loss of live coral cover and subsequent decreases in topographic complexity lead to declines in the abundance of some taxa, while others are unaffected or even increase in abundance following bleaching events (46). Body size, mobility, and specialization of habitat, diet, and recruitment drive variation in bleaching sensitivity (46–48). To estimate the differing sensitivity to bleaching of fisheries target taxa on each island, we identified the taxa that accounted for at least 10% of cumulative landings from 2005-2014, using reconstructed landings data (49), and calculated an index of sensitivity for each taxon based on habitat usage (50) and adult home range size (51,52) (Table S3), assuming that taxa that use reef habitats and have smaller adult home ranges would be more sensitive to bleaching.

In determining the taxa that accounted for at least 10% of cumulative landings from 2005-2014, we only included catches from each island’s own EEZ. This method yielded a list of 1-4 top landed taxa for each island. We calculated an index of sensitivity for each fish and macroinvertebrate taxon based on habitat usage and adult home range size. Species that utilize coral reef habitats are more sensitive to coral loss than pelagic species (48). Using FAO taxonomic descriptions, we assigned habitat utilization scores to each taxon (50). The top landed taxa in the Caribbean include pelagic and coastal pelagic taxa, and taxa such as snappers (Lutjanidae), spiny lobsters (Palinuridae), and grunts (Haemulidae) that utilize both coral reef and hard bottom habitat. We assigned a value of “1” to taxa that do not utilize coral reefs (such as Scombrid fish, *Lobatus gigas* [queen conch], and *Coryphaena hippurus* [dolphinfish]), while taxa that make partial use of coral reefs received a value of 0.5. Partial reef users include taxa that are dependent on coral reefs for specific life history stages and those that use coral reefs as well as other habitats. None of the top landed taxa were entirely reef-based.

We used the area where an adult individual of a species spends most of its time, or the size of the adult home range (51), as a proxy for mobility and spatial flexibility. We assumed that a coral-dependent species with a small adult home range would be more severely impacted by a localized bleaching event than a species with a larger home range, whose territory may encompass multiple reef patches. For reef fish taxa, we used home range sizes reported in Green *et al*. (2015), when available. Otherwise, we estimated home ranges using an equation from Kramer and Chapman based on body size: *log(home range) = -3.75 + 2.35 x log(mean fork length* (52). We used body lengths as reported in FishBase, converting to fork length when necessary (53). When FishBase included multiple length-length conversion equations, we took the average home range value provided by the range of length conversions. Green *et al*. (2015) did not include home ranges for pelagic species, and since the Kramer and Chapman (1999) equation does not apply to non-reef species, we did not calculate home ranges for pelagic taxa^[[1]](#footnote-1)^. Where top landed taxa were reported at the genus, family, or order level, we used geographic ranges and human use patterns reported by FishBase, SeaLifeBase (54), and the IUCN Red List (25) to identify all species in the relevant taxonomic group that are found in the Caribbean and harvested in fisheries, then calculated the average home range based on all the relevant species. For macroinvertebrates, we used home ranges identified through literature searches (*e.g.*, from tagging studies), assuming a circular home range and estimating the diameter of the home range in kilometers, following Green *et al*.’s method (51).

To combine the information we compiled on habitat use and adult home range (Table S3) into a single sensitivity score for each taxa, we multiplied the habitat score by the size of the home range. For many islands, the Sea Around Us reported the largest portion of the catch as “Marine fishes not identified” or “Marine finfishes not identified.” For these categories, we calculated the average sensitivity across all fish species included in our database of home ranges and habitat types (taxon-level information for each of the most-landed taxa in the Caribbean, excluding the macroinvertebrates); “marine fishes” and “marine finfishes” have the same taxon sensitivity score (0.333).

**Table S3.** Usage of coral reef habitat and adult home ranges (in km) for the main target taxa in the Caribbean. Taxa that do not use coral reef habitats are coded as “Not reef”; taxa that use coral reef habitats are coded as “Part reef.” Adult home range data is only provided for taxa that use reefs. All habitat data is from Carpenter (2002) (50).

| Target taxon | Habitat | Home range (km) | Home range method | Taxa included in calculation | Body size sources | Body length conversion sources |
| --- | --- | --- | --- | --- | --- | --- |
| *Acanthocybium solandri* | Not reef | NA | NA | NA | NA | NA |
| Clupeidae | Not reef | NA | NA | NA | NA | NA |
| Clupeiformes | Not reef | NA | NA | NA | NA | NA |
| Coryphaena | Not reef | NA | NA | NA | NA | NA |
| *Coryphaena hippurus* | Not reef | NA | NA | NA | NA | NA |
| *Decapterus macarellus* | Not reef | NA | NA | NA | NA | NA |
| Haemulidae | Part reef | 0.192 | Kramer-Chapman | Caribbean Haemulidae with body length data in FishBase | Courtenay & Sahlman 1978; Cervigón et al. 1992 | Billings & Munro 1974; Manooch & Barans 1982; Froese & Pauly 2017 |
| *Hemiramphus brasiliensis* | Not reef | NA | NA | NA | NA | NA |
| Labridae | Part reef | 0.103 | Kramer-Chapman | Caribbean Labridae with body length data in FishBase | Gomon 1978; Cervigón et al. 1992; Schneider 1990 | Froese & Pauly 2017 |
| *Lobatus gigas* | Not reef | NA | NA | NA | NA | NA |
| Lutjanidae | Part reef | 0.396 | Kramer-Chapman | Caribbean Lutjanidae with body length data in FishBase | Allen 1985; Murray et al. 1992; Smith 1997; Cervigón 1993 | Murray & Moore 1992; Froese & Pauly 2017; Claro & García-Arteaga 1994; Burton 2002; Thompson & Munro 1983; Manooch, C.S. & Matheson 1983; Claro et al. n.d.; Claro 1981; Manooch, C.S. & Mason 1984; Johnson 1983; Grimes 1978 |
| Lutjanus | Part reef | 0.616 | Kramer-Chapman | Caribbean snappers of the *Lutjanus* genus with body length data in FishBase | Allen 1985; Smith 1997 | Froese & Pauly 2017; Claro & García-Arteaga 1994; Burton 2002; Thompson & Munro 1983; Manooch, C.S. & Matheson 1983; Claro 1981; Manooch, C.S. & Mason 1984; Claro et al. n.d. |
| *Lutjanus vivanus* | Part reef | 0.263 | Kramer-Chapman | *Lutjanus vivanus* | Allen 1985 | Froese & Pauly 2017; Manooch & Mason 1984 |
| *Makaira nigricans* | Not reef | NA | NA | NA | NA | NA |
| *Ocyurus chrysurus* | Part reef | 0.1 | Green et al. 2015 | *Ocyurus chrysurus* | Allen 1985 | Froese & Pauly 2017; Johnson 1983; Thompson & Munro 1983 |
| Panulirus | Part reef | 0.664 | Literature review | Caribbean spiny lobsters of the *Panulirus* genus with body length data in FishBase | Bertelsen & Hornbeck 2009; Lozano-Álvarez et al. 2002 | NA |
| *Panulirus argus* | Part reef | 1.128 | Literature review | *Panulirus argus* | Bertelsen & Hornbeck 2009 | NA |
| Scombridae | Not reef | NA | NA | NA | NA | NA |
| *Selar crumenophthalmus* | Not reef | NA | NA | NA | NA | NA |
| Serranidae | Part reef | 0.424 | Kramer-Chapman | Caribbean Serranidae with body length data in FishBase | Heemstra & Randall 1993; Smith 1978; Cervigón et al. 1992; Courtenay 1981 | Froese & Pauly 2017; Heemstra & Randall 1993; Johnson & Collins 1994 |
| Sphyraenidae | Part reef | 1.254 | Kramer-Chapman | Caribbean Sphyraenidae with body length data in FishBase | Vergara 1978; De Sylva 1981; Cervigon et al. 1992 | Froese & Pauly 2017 |
| *Thunnus albacares* | Not reef | NA | NA | NA | NA | NA |

We used the taxon-specific scores to calculate island-level sensitivity scores. We inverted the taxon sensitivity scores so that taxonomic sensitivity increased as the sensitivity score increased (inverted sensitivity score = 1 – sensitivity score). Then, for each taxon in each island, we calculated its score as the taxon-specific sensitivity score multiplied by the percentage of the catch comprised by that species. We summed the taxa within each island for an island-level score (Table S4). Higher scores indicate greater sensitivity to bleaching, reflecting more reef habitat dependence and smaller home ranges.

**Table S4.** Target taxa sensitivity to bleaching.

| Island | Target taxon | Portion of the catch | Taxon sensitivity | Island score |
| --- | --- | --- | --- | --- |
| Anguilla | Marine fishes | 0.212 | 0.333 | 0.304 |
|  | Panulirus | 0.130 | 0.668 |  |
|  | *Panulirus argus* | 0.130 | 0.436 |  |
|  | Serranidae | 0.115 | 0.788 |  |
| Antigua & Barbuda | Lutjanidae | 0.126 | 0.802 | 0.258 |
|  | Marine fishes | 0.471 | 0.333 |  |
| Aruba | Marine fishes | 0.206 | 0.333 | 0.069 |
| Bahamas | *Lobatus gigas* | 0.177 | 0.726 | 0.328 |
|  | *Panulirus argus* | 0.457 | 0.436 |  |
| Barbados | Coryphaena | 0.127 | 0.000 | 0.196 |
|  | Marine fishes | 0.588 | 0.333 |  |
| St. Barthelemy | Marine fishes | 0.170 | 0.333 | 0.057 |
| Bonaire | *Acanthocybium solandri* | 0.103 | 0.000 | 0.102 |
|  | Marine fishes | 0.308 | 0.333 |  |
| British Virgin Islands | *Ocyurus chrysurus* | 0.245 | 0.950 | 0.233 |
| Cayman Islands | Lutjanidae | 0.115 | 0.802 | 0.092 |
|  | *Makaira nigricans* | 0.819 | 0.000 |  |
| Cuba | Marine fishes | 0.115 | 0.333 | 0.140 |
|  | *Panulirus argus* | 0.233 | 0.436 |  |
| Curaçao | *Acanthocybium solandri* | 0.188 | 0.000 | 0.091 |
|  | Coryphaena | 0.122 | 0.000 |  |
|  | Marine fishes | 0.274 | 0.333 |  |
| Dominica | *Coryphaena hippurus* | 0.179 | 0.000 | 0.041 |
|  | *Hemiramphus brasiliensis* | 0.126 | 0.000 |  |
|  | *Makaira nigricans* | 0.125 | 0.000 |  |
|  | Marine fishes | 0.123 | 0.333 |  |
| Dominican Republic | Haemulidae | 0.167 | 0.904 | 0.334 |
|  | Lutjanidae | 0.163 | 0.802 |  |
|  | Marine fishes | 0.159 | 0.333 |  |
| Grenada | Marine fishes | 0.130 | 0.333 | 0.043 |
|  | *Thunnus albacares* | 0.188 | 0.000 |  |
| Guadeloupe | Marine fishes | 0.685 | 0.333 | 0.228 |
|  | Scombridae | 0.127 | 0.000 |  |
| Haiti | Labridae | 0.187 | 0.948 | 0.258 |
|  | Marine fishes | 0.240 | 0.333 |  |
| Jamaica | *Lobatus gigas* | 0.111 | 0.726 | 0.253 |
|  | Marine finfishes | 0.154 | 0.333 |  |
|  | Marine fishes | 0.208 | 0.333 |  |
|  | Sphyraenidae | 0.139 | 0.373 |  |
| Martinique | Clupeidae | 0.205 | 0.000 | 0.067 |
|  | Clupeiformes | 0.158 | 0.000 |  |
|  | Marine fishes | 0.201 | 0.333 |  |
| Montserrat | Marine fishes | 0.408 | 0.333 | 0.136 |
| Puerto Rico | Lutjanidae | 0.214 | 0.802 | 0.248 |
|  | Marine fishes | 0.229 | 0.333 |  |
| Saba | *Lutjanus vivanus* | 0.160 | 0.869 | 0.367 |
|  | *Panulirus argus* | 0.523 | 0.436 |  |
| St. Eustatius | *Lutjanus vivanus* | 0.160 | 0.869 | 0.367 |
|  | *Panulirus argus* | 0.523 | 0.436 |  |
| St. Kitts & Nevis | Marine fishes | 0.372 | 0.333 | 0.124 |
| St. Lucia | *Coryphaena hippurus* | 0.214 | 0.000 | 0.065 |
|  | Marine fishes | 0.194 | 0.333 |  |
|  | Scombridae | 0.269 | 0.000 |  |
| St. Maarten | Lutjanus | 0.167 | 0.692 | 0.467 |
|  | *Lutjanus vivanus* | 0.312 | 0.869 |  |
|  | Marine fishes | 0.241 | 0.333 |  |
| St. Martin | Marine fishes | 0.161 | 0.333 | 0.053 |
| St. Vincent & the Grenadines | *Decapterus macarellus* | 0.169 | 0.000 | 0.087 |
|  | Marine fishes | 0.131 | 0.333 |  |
|  | *Panulirus argus* | 0.100 | 0.436 |  |
|  | *Selar crumenophthalmus* | 0.132 | 0.000 |  |
| Trinidad & Tobago | Lutjanidae | 0.107 | 0.802 | 0.128 |
|  | Marine fishes | 0.125 | 0.333 |  |
|  | Scombridae | 0.254 | 0.000 |  |
| Turks & Caicos | Marine fishes | 0.818 | 0.333 | 0.272 |
| US Virgin Islands | Marine fishes | 0.564 | 0.333 | 0.188 |

### Ecological Recovery Potential

We calculated ecological recovery potential (ERP) as the average six indicators— coral cover (*Cov_C_*), inverted macroalgal cover (*Cov_MA_*), Scaridae biomass (*Scar)*, *Diadema antillarum* density (*Diad)*, and coral and reef fish species richness ((*SR_C_* and *SR_RF_*, respectively)— using equation (S3):

$ES=\frac{\left( {Cov}_{C}+ {Cov}_{MA}+ Scar+ Diad+ {SR}_{C}+ {SR}_{RF} \right)}{6}$ (S3)

We selected these indicators for ecological recovery potential based on their importance for reef recovery, their use in previously published analyses of vulnerability (e.g., 4,83), and the availability of consistent data across the region. While some studies have weighted the various indicators of ecological recovery potential using expert opinion (84), we opted to use equal weighting across the indicators because we did not have sufficient information to weight their importance in the specific context of Caribbean coral reefs.

Coral cover on reefs: We used the percentage of live coral cover on reefs as an indicator of recovery potential, although there is some controversy about this relationship (85,86). Given the absence of coordinated, simultaneous monitoring efforts across the region, we relied on surveys of coral cover from different sources, including region-wide data syntheses (22,83), primary literature, and raw survey data (43). We restricted our analysis to surveys that took place after the 2005 bleaching event (Table S5). Where reports included a range of coral cover values from different sites around an island, we used the average value. We used ecoregional averages to fill gaps in the data (see “Ecoregional Gap Filling”).

Using data from different survey sources (academic studies, government monitoring, and trained volunteers, as in the case of Reef Check) may introduce different forms of bias. However, volunteer programs can generate results aligning with those produced by professionals (87–89). Reef Check, AGRRA, and other monitoring programs have been used successfully in scientific analyses (90–92), and we determined that these data sources would provide more accurate results than relying on ecoregional averages to fill the gaps for each island that lacked recent data from a professional monitoring program. However, we preferentially used data from professional monitoring programs when those were available.

###

Macroalgal cover on reefs: We used the percentage of macroalgal cover on reefs as a negative indicator of recovery potential, as macroalgal growth reduces coral recruitment and growth, suppressing recovery after bleaching events (93–95). As with live coral cover, we compiled data from reef surveys conducted since 2005. We used data from the same survey year (and the same survey whenever possible) for the macroalgal and coral cover values. For studies that reported macroalgal cover values for different sites, we used the average. We used ecoregional averages to fill data gaps (Table S5).

We tested for a correlation between the proportion of coral cover and the proportion of macroalgal cover on reefs, and found that they were not highly correlated (Pearson’s correlation coefficient = -0.097, p = 0.6). This validated our assumption that these two variables capture different aspects of reef health, and we used both variables to construct the index of ecological recovery potential, inverting the algal cover values so that a higher value indicated greater recovery potential.

###

Scaridae biomass: Grazing by herbivores such as Scaridae (parrotfish) and *Diadema antillarum* (long-spined sea urchin) reduces macroalgal cover, providing substrates for coral recruitment and growth (95–98). For the past three decades, Scaridae have been the most important grazers on Caribbean reefs (93). We used the biomass of Scaridae (in grams per square meter) as an indicator of coral reef recovery potential to reflect their role in grazing macroalgae and thus facilitating coral recruitment and growth. We acquired data on Scaridae biomass from reports synthesizing regional data, the Reef Check database (99), and primary literature (Table S5). Where the primary literature included bar graphs depicting biomass without providing the actual biomass values (e.g., Roff et al. 2011 and Williams et al. 2017), we used the online application WebPlotDigitizer to extract the values (100).

We scaled the Scaridae biomass values by dividing the observed and estimated values by a baseline of 47 g/m^2^, a representative unfished biomass for Caribbean Scaridae (101). The Scaridae biomass values for each island thus represent the proportion of a regional, unfished baseline. We capped the scaled Scaridae biomass value at 1.0, assuming that past the threshold of the historical unfished baseline, additional Scaridae biomass would not further enhance recovery potential.

Diadema antillarum density: Prior to their mass mortality in 1983, *Diadema antillarum* were the most important herbivore on Caribbean reefs, due to their high grazing rates (93). We compiled data on *Diadema* density from regional data syntheses and primary literature (Table S5). We scaled the *Diadema* densities by dividing the observed densities by a baseline density of 7.7 individuals per square meter, the mean density from sites across the Caribbean from 1970-1983 (102). The *Diadema* values for each island thus represent the proportion of the historical baseline.

**Table S5.** Coral cover, macroalgal cover, Scaridae biomass, and *Diadema* density.

| Ecoregion | Island | Coral cover (proportion) | Source | Macroalgal cover (proportion) | Source | Scaridae biomass (g/m^2^) | Source | *Diadema* density (individuals/m^2^) | Source |
| --- | --- | --- | --- | --- | --- | --- | --- | --- | --- |
| Bahamanian | Bahamas | 0.093 | Jackson et al. 2014 | 0.569 | Jackson et al. 2014 | 16.142 | Jackson et al. 2014 | 0.157 | Jackson et al. 2014 |
|  | Turks & Caicos | 0.032 | Byce 2012 | 0.200 | Byce 2012 | 7.823 | Roff et al. 2011 | 0.137 | Reef Check |
| Eastern Caribbean | Anguilla | 0.041 | Wynne 2010 | 0.134 | Wynne 2010 | 14.662 | Ecoregional average | 0.065 | Wynne 2010 |
|  | Antigua & Barbuda | 0.038 | Jackson et al. 2014 | 0.138 | Jackson et al. 2014 | 15.981 | Jackson et al. 2014 | 0.219 | Jackson et al. 2014 |
|  | Barbados | 0.153 | Jackson et al. 2014 | 0.082 | Jackson et al. 2014 | 9.608 | Williams et al. 2017*^a^* | 0.004 | Jackson et al. 2014 |
|  | British Virgin Islands | 0.169 | Jackson et al. 2014 | 0.149 | Jackson et al. 2014 | 10.125 | Jackson et al. 2014 | 0.157 | Reef Check |
|  | Dominica | 0.086 | Jackson et al. 2014 | 0.001 | Jackson et al. 2014 | 14.662 | Ecoregional average | 2.754 | Reef Check |
|  | Grenada | 0.203 | Jackson et al. 2014 | 0.688 | Jackson et al. 2014 | 14.662 | Ecoregional average | 0.030 | Reef Check |
|  | Guadeloupe | 0.186 | Jackson et al. 2014 | 0.338 | Jackson et al. 2014 | 24.400 | Jackson et al. 2014 | 1.520 | Reef Check |
|  | Martinique | 0.200 | Rémi et al. 2012 | 0.140 | Rémi et al. 2012 | 20.400 | Jackson et al. 2014 | 2.870 | Rémi et al. 2012 |
|  | Montserrat | 0.122 | Ecoregional average | 0.226 | Ecoregional average | 4.773 | Waitt Institute 2016 | 0.758 | Reef Check |
|  | Saba | 0.048 | Toller et al. 2010 | 0.441 | Toller et al. 2010 | 8.000 | Noble et al. 2013 | 0.000 | Meesters 2010 |
|  | St. Barthelemy | 0.108 | Jackson et al. 2014 | 0.530 | Jackson et al. 2014 | 17.100 | Jackson et al. 2014 | 0.000 | PARETO 2012 |
|  | St. Eustatius | 0.049 | Debrot et al. 2014 | 0.127 | Debrot et al. 2014 | 34.000 | de Graaf et al. 2015*^b^* | 0.001 | Jackson et al. 2014 |
|  | St. Kitts & Nevis | 0.110 | Jackson et al. 2014 | 0.383 | Jackson et al. 2014 | 9.160 | Jackson et al. 2014 | 0.007 | Jackson et al. 2014 |
|  | St. Lucia | 0.096 | Jackson et al. 2014 | 0.081 | Jackson et al. 2014 | 26.700 | Williams et al. 2017*^a^* | 1.958 | Reef Check |
|  | St. Maarten | 0.186 | Perry et al. 2015 | 0.226 | Ecoregional average | 14.662 | Ecoregional average | 0.004 | Jackson et al. 2014 |
|  | St. Martin | 0.080 | Rémi et al. 2012 | 0.200 | Rémi et al. 2012 | 12.100 | Jackson et al. 2014 | 0.742 | Reef Check |
|  | St. Vincent & the Grenadines | 0.249 | Jackson et al. 2014 | 0.004 | Jackson et al. 2014 | 5.912 | Jackson et al. 2014 | 0.442 | Jackson et al. 2014 |
|  | US Virgin Islands | 0.069 | Jackson et al. 2014 | 0.179 | Jackson et al. 2014 | 7.013 | Jackson et al. 2014 | 0.074 | Jackson et al. 2014 |
| Greater Antilles | Cayman Islands | 0.246 | Jackson et al. 2014 | 0.314 | Jackson et al. 2014 | 9.638 | Jackson et al. 2014 | 0.014 | Jackson et al. 2014 |
|  | Cuba | 0.276 | Jackson et al. 2014 | 0.274 | Jackson et al. 2014 | 10.342 | Jackson et al. 2014 | 0.217 | Jackson et al. 2014 |
|  | Dominican Republic | 0.213 | Jackson et al. 2014 | 0.089 | Jackson et al. 2014 | 8.584 | Nugraha 2016 | 0.289 | Reef Check |
|  | Haiti | 0.107 | Jackson et al. 2014 | 0.657 | Jackson et al. 2014 | 8.614 | Ecoregional average | 0.104 | Reef Check |
|  | Jamaica | 0.175 | Chatenoux & Wolf 2013 | 0.347 | Chatenoux & Wolf 2013 | 7.568 | Nugraha 2016 | 1.482 | Reef Check |
|  | Puerto Rico | 0.109 | Jackson et al. 2014 | 0.104 | Jackson et al. 2014 | 6.935 | Jackson et al. 2014 | 0.981 | Reef Check |
| Southern Caribbean | Aruba | 0.252 | Ecoregional average | 0.198 | Ecoregional average | 39.154 | Ecoregional average | 0.036 | Reef Check |
|  | Bonaire | 0.384 | Steneck 2015 | 0.109 | Steneck 2015 | 30.000 | Arnold 2015 | 0.300 | Boyle 2015 |
|  | Curaçao | 0.224 | Jackson et al. 2014 | 0.081 | Jackson et al. 2014 | 17.501 | Nugraha 2016 | 0.007 | Reef Check |
|  | Trinidad & Tobago | 0.149 | Mallela et al. 2010 | 0.405 | Mallela et al. 2010 | 69.962 | Alemu 2014 | 0.126 | Reef Check |

*^a^* value extracted from a bar graph using WebPlotDigitizer(100); *^b^* value derived from 2015 GCRMN herbivore data, subtracting the mass attributed to Acanthuridae (surgeonfish).

*Coral species richness:* We included the richness of Scleractinian coral species on the reefs as an indicator of recovery potential, given evidence that coral richness promotes recovery by increasing the likelihood that resistant and quickly growing species will be present in the assemblage (119), although there is debate as to the nature of this relationship (85,86).

Where possible, we used data on coral species richness from a recent survey of the Caribbean region (120). For gaps in this data set, we used individual studies and surveys (22,32,121,122). In addition, where there were targeted surveys published more recently than Miloslavich et al. (2010), we used those surveys instead (123). We were unable to locate data on coral species richness for seven islands. For these islands, we used the average coral richness for their ecoregions as estimates of their species richness. We scaled the coral species richness value by dividing each island’s value by the total number of Scleractinian coral species present in the Caribbean (n = 287 (120)).

**Table S6.** Observed and estimated coral species richness.

| Ecoregion | Island | Coral species richness | Source |
| --- | --- | --- | --- |
| Bahamanian  (average: 59) | Bahamas | 59 | Ecoregional average |
|  | Turks & Caicos | 59 | Churchyard et al. 2016 |
| Eastern Caribbean (average: 38) | Anguilla | 38 | Ecoregional average |
|  | Antigua & Barbuda | 32 | Bouchon et al. 2008 |
|  | Barbados | 29 | Oxenford et al. 2008 |
|  | British Virgin Islands | 50 | Churchyard et al. 2016 |
|  | Dominica | 46 | Steiner 2015 |
|  | Grenada | 33 | Bouchon et al. 2008 |
|  | Guadeloupe | 38 | Ecoregional average |
|  | Martinique | 30 | Marechal & Peres 2006 (across all sites) |
|  | Montserrat | 30 | Churchyard et al. 2016 |
|  | Saba | 35 | Bouchon et al. 2008 |
|  | St. Barthelemy | 38 | Ecoregional average |
|  | St. Eustatius | 35 | Bouchon et al. 2008 |
|  | St. Kitts & Nevis | 53 | Rusk 2014 |
|  | St. Lucia | 38 | Ecoregional average |
|  | St. Maarten | 38 | Ecoregional average |
|  | St. Martin | 30 | Diaz & Cuzange 2009 (2008 data) |
|  | St. Vincent & the Grenadines | 38 | Ecoregional average |
|  | US Virgin Islands | 48 | Rogers et al. 2008 |
| Greater Antilles (average: 71) | Cayman Islands | 65 | Churchyard et al. 2016 |
|  | Cuba | 72 | Miloslavich et al. 2010 |
|  | Dominican Republic | 72 | Miloslavich et al. 2010 |
|  | Haiti | 72 | Miloslavich et al. 2010 |
|  | Jamaica | 72 | Miloslavich et al. 2010 |
|  | Puerto Rico | 72 | Miloslavich et al. 2010 |
| Southern Caribbean (average: 58) | Aruba | 68 | Miloslavich et al. 2010 |
|  | Bonaire | 68 | Miloslavich et al. 2010 |
|  | Curaçao | 68 | Miloslavich et al. 2010 |
|  | Trinidad & Tobago | 30 | Mouillot et al. 2014; Nystrom et al. 2008 |

*Fish species richness*: Reef fish species richness, as a proxy for functional diversity and redundancy, is another indicator of reef recovery potential (128,129). More diverse reef fish assemblages exhibit less biomass fluctuation with rising and more variable water temperatures, implying increased resilience to climate change impacts (130). In addition, diverse herbivore assemblages are more effective at suppressing macroalgal growth (131).

A recent biogeographical analysis found that island area was the most important factor in determining reef fish richness in the Caribbean (132). We used the reef fish species richness values reported in Sandin *et al*.’s (2008) Table S2 for the 24 islands included in that study. Sandin *et al*. (2008) reported a single value for the island of Hispaniola (350); we assigned this value to both the Dominican Republic and Haiti, assuming that the two portions of the island have the same reef fish assemblage. For the five islands missing from Sandin *et al*.’s analysis, we fit a linear regression between island area and reef fish species richness, using data on island area that we compiled (133–135). We did not use the island areas reported by Sandin *et al*. because we could not access their original data set and they did not report areas for the islands for which we needed to estimate species richness. We used our data on island areas to estimate reef fish species richness for the missing five islands using equation (S4) (p-value = 0.001):

$\ln\left( SR \right)=5.5279+0.0454*\ln\left( IA \right),$ (S4)

where *SR* is the reef fish species richness and *IA* is the island area (in km^2^). We scaled the reef fish species richness value by dividing each island’s value by the total number of reef-associated fish species occurring across the 30 Caribbean islands (n = 588), based on records in FishBase (53). We acknowledge that this method of estimating reef fish species richness does not account for other factors that may influence reef fish diversity, such as fishing pressure and the diversity and quality of habitats.

**Table S7.** Reported and estimated reef fish species richness (SR).

| Island | Island area (km^2^) | Reef fish SR |
| --- | --- | --- |
| Anguilla | 91*^a^* | 305*^d^* |
| Antigua & Barbuda | 442.6*^a^* | 334*^d^* |
| Aruba | 180*^a^* | 346*^d^* |
| Bahamas | 10,010*^a^* | 462*^d^* |
| Barbados | 430*^a^* | 344*^d^* |
| St. Barthelemy | 25*^a^* | 291*^e^* |
| Bonaire | 294*^b^* | 326*^e^* |
| British Virgin Islands | 151*^a^* | 347*^d^* |
| Cayman Islands | 264*^a^* | 328*^d^* |
| Cuba | 109,820*^a^* | 485*^d^* |
| Curaçao | 444*^a^* | 358*^d^* |
| Dominica | 751*^a^* | 327*^d^* |
| Dominican Republic | 48,320*^a^* | 350*^d^* |
| Grenada | 344*^a^* | 321*^d^* |
| Guadeloupe | 1,628*^c^* | 295*^d^* |
| Haiti | 27,560*^a^* | 350*^d^* |
| Jamaica | 10,831*^a^* | 387*^d^* |
| Martinique | 1,128*^c^* | 314*^d^* |
| Montserrat | 102*^a^* | 286*^d^* |
| Puerto Rico | 8,959*^a^* | 384*^d^* |
| Saba | 13*^b^* | 283*^e^* |
| St. Eustatius | 21*^b^* | 289*^e^* |
| St. Kitts & Nevis | 261*^a^* | 312*^d^* |
| St. Lucia | 606*^a^* | 334*^d^* |
| St. Maarten | 34*^a^* | 295*^e^* |
| St. Martin | 54.4*^a^* | 302*^e^* |
| St. Vincent & the Grenadines | 389*^a^* | 329*^d^* |
| Trinidad & Tobago | 5,128*^a^* | 459*^d^* |
| Turks & Caicos | 948*^a^* | 271*^d^* |
| US Virgin Islands | 346*^a^* | 381*^d^* |

*^a^* CIA 2017; *^b^* de Bettencourt & Imminga-Berends 2015; *^c^* PAHO 2013; *^d^* Sandin et al. 2008; *^e^* value estimated from regression.

## B. Components of Socioeconomic Vulnerability

### Socioeconomic Exposure

We calculated ecological vulnerability as composite index of ecological exposure, sensitivity, and recovery potential (*EV* = *EE* + *ES* – *ERP*). We then rescaled these values to be bounded between 0 and 1 to serve as the socioeconomic exposure (*SE*) of each island using equation (S5):

$SS=\frac{\left( EE + ES - ERP \right)+1}{3}$ $(S5)$

### Socioeconomic Sensitivity

We calculated socioeconomic sensitivity (*SS*) as a function of the contribution to an island’s GDP from reef-based tourism (*GDP_T_*) and reef fisheries (*GDP_F_*), the proportion of the population engaged in small-scale fishing activity (Pop*_SSF_*), and the proportion of locally-landed seafood that is consumed on-island (*LF*), using equation (S6):

$SS=0.5*{GDP}_{T}+0.5*\frac{{Pop}_{SSF}+LF+{GDP}_{F}}{3}$ $(S6)$

####

Percent of population engaged in small-scale fishing*:* We calculated the percentage of each island’s population that engages in small-scale fishing as an indicator of socioeconomic dependence on coral reef fisheries for local employment and food security (Table S8). We did not include large-scale industrial fishers in this analysis, since most reef fishers in the Caribbean are small-scale. We did not attempt to distinguish between small-scale fishers who target reef species and those who target pelagic or other non-reef species due to the mixed-catch nature of artisanal Caribbean fisheries (136,137).

We acknowledge the potential for inaccurate estimates of the numbers of small-scale fishers in the data we compiled, due to the lack of accurate censuses on many islands, differences in the reporting of registered and unregistered fishers (138), and the length of time between the different reef fisher surveys and estimates we compiled (e.g., 13 years). We likely underestimate the proportion of the population engaged in small-scale fishing, leading to conservative estimates of sensitivity. Due to inadequate data, we also did not account for other forms of employment in the small-scale fishing sector, such as fish cleaners and mongers, but including employment in these sectors would likely increase our estimates of sensitivity (2).

For Aruba, we used an estimate of 2800 small-scale fishers, based on the numbers of participants in each of the top three small-scale fisheries (1000 targeting *Acanthocybium solandri* [wahoo], 900 targeting Serranidae [grouper], and 900 Lutjanidae [snapper]) (139). However, we acknowledge that this may over-estimate the number of small-scale fishers, as individual fishers may be active in multiple fisheries.

**Table S8.** The proportion of each island’s population that works as small-scale fishers.

| Island | Number of small-scale fishers | Source | Total population | Source | Proportion small-scale fishers |
| --- | --- | --- | --- | --- | --- |
| Anguilla | 500 | Gumbs & Rawlins 2007 | 13,000 | UNDESA 2015 (2007 data) | 0.0385 |
| Antigua & Barbuda | 1,521 | Horsford 2015 (2010 data) | 87,233 | World Bank 2017 (2010 data) | 0.0174 |
| Aruba | 2,800 | Boekhoudt 2015 | 104,000 | World Bank 2017 (2015 data) | 0.0269 |
| Bahamas | 9,300 | CRFM 2013 (2005 data)*^a^* | 329,243 | World Bank 2017 (2005 data) | 0.0282 |
| Barbados | 2,200 | McConney 2011 (2004 data) | 273,091 | World Bank 2017 (2004 data) | 0.0081 |
| Bonaire | 80 | Johnson & Jackson 2015 (2010 data) | 13,400 | PAHO 2012 (2010 data) | 0.0060 |
| British Virgin Islands | 410 | Ramdeen et al. 2014a (2010 data) | 28,037 | CARICOM 2015 (2010 data) | 0.0146 |
| Cayman Islands | 5,658 | Meier et al. 2011 | 56,580 | World Bank 2017 (2011 data) | 0.1000 |
| Cuba | 3,900 | FAO 2015 (2010 data)*^a^* | 11,275,199 | World Bank 2017 (2006 data) | 0.0003 |
| Curaçao | 200 | Johnson 2011 | 148,703 | World Bank 2017 (2010 data) | 0.0013 |
| Dominica | 1,340 | FAO 2016a (2013 data) | 72,005 | FAOSTAT 2017 (2013 data) | 0.0186 |
| Dominican Republic | 11,600 | Mateo 2015 | 10,528,000 | World Bank 2017 (2015 data) | 0.0011 |
| Grenada | 1,931 | Baldeo 2011 (2004 data) | 102,657 | World Bank 2017 (2004 data) | 0.0188 |
| Guadeloupe | 1,200 | Bouchon et al 2008 | 401,784 | Cotis 2011 (2008 data) | 0.0030 |
| Haiti | 52,000 | Ramdeen et al. 2012a; MARNDR 2010 | 9,999,617 | World Bank 2017 (2010 data) | 0.0052 |
| Jamaica | 31,500 | Carr & Heyman 2009; Murray 2007*^b^* | 2,662,481 | World Bank 2017 (2007 data) | 0.0118 |
| Martinique | 2,500 | Bouchon et al. 2008 | 396,000 | UNDESA 2015 (2008 data) | 0.0063 |
| Montserrat | 101 | Ponteen 2014 | 5,000 | UNDESA 2015 (2014 data) | 0.0202 |
| Puerto Rico | 1,163 | Valle-Esquivel et al. 2011 (2002 data) | 3,823,701 | World Bank 2017 (2002 data) | 0.0003 |
| Saba | 50 | Bouchon et al. 2008 | 1,537 | Central Bureau of Statistics 2009 | 0.0325 |
| St. Barthelemy | 43 | Jadot 2016 | 9,427 | INSEE 2017 (2014 data) | 0.0046 |
| St. Martin | 11 | Diaz & Cuzange 2009 (2008 data) | 29,376 | World Bank 2017 (2008 data) | 0.0004 |
| St. Eustatius | 24 | Dilrosun 2004a (2004 data) | 2,525 | Central Bureau of Statistics 2009 | 0.0095 |
| St. Maarten | 50 | Dilrosun 2004b (2004 data) | 35,316 | World Bank 2017 (2004 data) | 0.0014 |
| St. Kitts & Nevis | 1,086 | Masters 2010 (2010 data) | 52,352 | World Bank 2017 (2010 data) | 0.0207 |
| St. Lucia | 2,556 | George et al 2015 | 180,890 | World Bank 2017 (2012 data) | 0.0141 |
| St. Vincent & the Grenadines | 980 | Masters 2010 (2010 data) | 109,316 | World Bank 2017 (2010 data) | 0.0090 |
| Trinidad & Tobago | 2,880 | Shing & Maharaj 2015 | 1,360,000 | World Bank 2017 (2015 data) | 0.0021 |
| Turks & Caicos | 500 | de Bettencourt & Imminga-Berends 2015 (2010 data) | 30,993 | World Bank 2017 (2010 data) | 0.0161 |
| US Virgin Islands | 383 | Kojis & Quinn 2006 (2003 data) | 108,085 | World Bank 2017 (2003 data) | 0.0035 |

*^a^* Industrial fishers not separated out; *^b^* Middle of range given

Percent of local fish that is consumed domestically: We compiled data on the percentage of each island’s fisheries landings that is consumed domestically as an indicator of the role that fisheries play in food security, using data on fisheries production and exports from the Food & Agriculture Organization (Table S9) (172,173). For each island, we calculated the mean annual proportion of fishery production that was not exported from 2011-2013 across all taxa and product groups, excluding landings of whales because they were reported in different units than the rest of the taxa (number of individuals, rather than tons) and because no whale exports were reported. The time span of 2011-2013 was chosen to utilize recent data in the FAO database while avoiding issues with the landings and exports for Saba, St. Eustatius, Bonaire, and Curaçao being reported together prior to 2011 (when they collectively formed the Netherlands Antilles). We also excluded production by inland fisheries. This calculation includes fish caught outside each island’s EEZ; the FAO data does not list the EEZ in which landings were originally caught.

**Table S9.** The percentage of locally landed seafood that is consumed on-island, based on fisheries production and export data from the FAO.

| Island | Proportion of seafood consumed domestically |
| --- | --- |
| Anguilla | 1.00 |
| Antigua & Barbuda | 0.98 |
| Aruba | 0.95 |
| Bahamas | 0.80 |
| Barbados | 0.93 |
| Bonaire | 1.00 |
| British Virgin Islands | 1.00 |
| Cayman Islands | 0.76 |
| Cuba | 0.81 |
| Curaçao | 0.24 |
| Dominica | 1.00 |
| Dominican Republic | 0.66 |
| Grenada | 0.69 |
| Guadeloupe | 1.00 |
| Haiti | 0.96 |
| Jamaica | 0.94 |
| Martinique | 1.00 |
| Montserrat | 1.00 |
| Puerto Rico | 1.00 |
| Saba | 1.00 |
| St. Barthelemy | 1.00 |
| St. Eustatius | 1.00 |
| St. Kitts & Nevis | 0.99 |
| St. Lucia | 1.00 |
| St. Maarten | 1.00 |
| St. Martin | 1.00 |
| St. Vincent & the Grenadines | 1.00 |
| Trinidad & Tobago | 0.70 |
| Turks & Caicos | 0.95 |
| US Virgin Islands | 1.00 |

Percent contribution to GDP from small-scale reef fisheries: We calculated the percentage of each island’s GDP derived from small-scale fisheries as an indicator of the overall sensitivity of the island’s economy to changes in fishery production. We used data on the landed value of reef fisheries (reef-associated fish, cephalopods, and invertebrates) in 2010 from the Sea Around Us (49) and data on the 2010 GDPs of the islands from the World Bank, as well as GDP and CPI data from government and agencies and national banks for the ten islands without GDP data in the World Bank database. We restricted the data on landed value to catches made by each island in its own EEZ. The Sea Around Us database reported the landings value for Saba and St. Eustatius together. We assumed that each island contributed to this value in proportion to the number of small-scale fishers active on the island. Saba has 50 small-scale fishers, while St. Eustatius has 24 (Table S8), so we allocated 67.57% of the reef fishery value to Saba and 32.43% to St. Eustatius (Table S10).

To scale the proportion of GDP comprised of reef fisheries value by the global maximum, we used the 2010 Sea Around Us reconstructed reef fishery landings values to calculate the 2010 reef fisheries value of each entity in their database. We divided these values by the 2010 World Bank GDP values for all entities that appeared in both databases (using constant 2010 USD) to calculate the proportion of each entity’s 2010 GDP that came from reef fishery landings. The global maximum contribution of reef fisheries to GDP was in Micronesia, where reef fisheries accounted for 13.5% of the GDP. To calculate scaled values of the proportion of GDP from reef fisheries in the Caribbean, we divided the proportion of GDP from reef fisheries in each island by 0.135.

**Table S10.** Contribution of reef fisheries value to island-level GDP (without scaling values by the global maximum contribution in 2010).

| Island | Value of landed reef fisheries (in 2010 US$) | GDP (in 2010 US$) | Proportion of GDP from reef fisheries (raw) |
| --- | --- | --- | --- |
| Anguilla | 6,918,931 | 123,477,405 | 0.056 |
| Antigua & Barbuda | 7,244,312 | 1,124,629,041 | 0.006 |
| Aruba | 730,569 | 2,346,321,247 | 3.11 x 10^-4^ |
| Bahamas | 131,223,985 | 7,838,938,167 | 0.017 |
| Barbados | 1,017,767 | 4,010,887,286 | 2.54 x 10^-4^ |
| Bonaire | 317,140 | 4,106,939,022 | 7.72 x 10^-5^ |
| British Virgin Islands | 13,439,722 | 1,109,759,712 | 0.012 |
| Cayman Islands | 26,463 | 1,414,445,771 | 1.87 x 10^-5^ |
| Cuba | 50,273,898 | 74,291,451,362 | 0.001 |
| Curaçao | 716,274 | 5,285,361,016 | 1.36 x 10^-4^ |
| Dominica | 1,154,293 | 482,719,495 | 0.002 |
| Dominican Republic | 73,903,893 | 59,076,716,719 | 0.001 |
| Grenada | 1,593,519 | 839,853,537 | 0.002 |
| Guadeloupe | 1,199,228 | 8,185,162,983 | 1.47 x 10^-4^ |
| Haiti | 25,818,972 | 8,025,496,719 | 0.003 |
| Jamaica | 33,025,577 | 12,795,195,103 | 0.003 |
| Martinique | 11,440,971 | 7,829,266,720 | 0.001 |
| Montserrat | 54,981 | 56,476,324 | 0.001 |
| Puerto Rico | 3,321,817 | 96,597,497,025 | 3.44 x 10^-5^ |
| Saba | 1,867,109 | 42,147,639 | 0.044 |
| St. Barthelemy | 759,491 | 245,095,312 | 0.003 |
| St. Eustatius | 896,212 | 97,407,876 | 0.009 |
| St. Kitts & Nevis | 4,546,999 | 784,955,911 | 0.006 |
| St. Lucia | 1,111,419 | 1,293,607,232 | 0.001 |
| St. Maarten | 133,055 | 280,000,000,000 | 4.75 x 10^-7^ |
| St. Martin | 3,037,966 | 414,664,424 | 0.007 |
| St. Vincent & the Grenadines | 4,445,650 | 671,759,728 | 0.007 |
| Trinidad & Tobago | 13,457,111 | 26,603,538,141 | 0.001 |
| Turks & Caicos | 13,656,672 | 661,249,621 | 0.021 |
| US Virgin Islands | 3,134,001 | 3,380,401,043 | 0.001 |

###

Percent of GDP from reef-based tourism*:* We estimated the contribution of reef-based tourism to an island’s annual GDP using a recent assessment of the value of reef tourism worldwide (174). In Spalding *et al*.’s (2017) assessment, total reef-based tourism expenditures represent the sum of on-reef and reef-adjacent tourism value. On-reef tourism expenditures represent direct spending on diving, snorkeling, and other activities on the reef, while reef-adjacent value represents indirect services provided by coral reefs, such as food and sheltered waters. Spalding *et al*. (2017) provide estimates of the total reef-associated tourist expenditures for 24 of the 30 islands in their supplementary materials. We compiled data on the 2013 GDP of each island using the World Bank’s dataset of GDP in constant 2010 US$, and converting to 2013 dollar equivalents using the Consumer Price Index (175,176). As in the case of the GDP data used to calculate the percentage of GDP from reef fisheries, we used the World Bank as a source for 20 islands and government and national bank sources to get data on the GDPs and CPIs of the remaining ten islands. Finally, we divided Spalding *et al*.’s estimated reef-based tourist expenditures by the island’s GDP to estimate the percentage of the GDP derived from reef-based tourism.

For the six islands that were not included in the Spalding *et al*. (2017) data table, we used data on the number of per capita foreign tourist arrivals to fill the gaps. We compiled data on the number of tourist arrivals (excluding cruise passengers) and the local populations for each island to calculate the per capita number of tourists (177–181)^[[2]](#footnote-2),^^[[3]](#footnote-3)^. For five islands, we were unable to find data on foreign tourist arrivals and local populations for the same year. In these cases, we used population estimates from within four years of the year for which we had tourism data, then estimated the population for the relevant year using recent population growth rates (182–184). We estimated a linear model to describe the relationship between the per capita number of tourists and the proportion of an island’s GDP that comes from reef-associated tourism (equation (S7)). We used this linear model to the fill the gaps in the Spalding *et al*. (2017) dataset (p << 0.001, adjusted R^2^ = 0.81):

$GDP_{t}=0.01397*T,$ (S7)

where *GDP_t_* is the proportion of the annual GDP from reef-associated tourism and *T* is the number of foreign tourists per capita.

As in the case of the contribution of reef fishery value to GDP, we scaled the contribution of reef tourism to each island’s GDP by dividing the raw proportion (“Tourism as proportion of GDP (raw)” in Table S11) by the global maximum contribution to GDP from reef tourism: 43.19% in Palau (174).

**Table S11.** The percentage of each island’s GDP from reef-based tourism, using data from Spalding *et al*. (2017) and per capita foreign tourist arrivals.

| Island | Population | Source | Foreign tourists | Source | GDP (millions of 2013 US$) | Source | Tourism as proportion of GDP (raw) | Source |
| --- | --- | --- | --- | --- | --- | --- | --- | --- |
| Anguilla | 16,352 | CIA 2017a*^a^* | 70,927 | CTO 2015 | 295.7 | Undata 2017a; AGSD 2014 | 0.067 | Spalding et al. 2017 |
| Antigua & Barbuda | 90,900 | World Bank 2017a | 249,316 | CTO 2015 | 1248.5 | World Bank 2017b | 0.053 | Spalding et al. 2017 |
| Aruba | 103,441 | World Bank 2017a | 1,072,082 | CTO 2015 | 2590.2 | Undata 2017d; OECD 2018 | 0.084 | Spalding et al. 2017 |
| Bahamas | 383,054 | World Bank 2017a | 1,421,860 | CTO 2015 | 8765.3 | World Bank 2017b | 0.060 | Spalding et al. 2017 |
| Barbados | 283,380 | World Bank 2017a | 519,598 | CTO 2015 | 4797.4 | World Bank 2017b | 0.038 | Spalding et al. 2017 |
| Bonaire | 18,413 | CBS 2017 | 133,000 | CBS 2015 | 387.0 | CBS 2018 | 0.233 | Spalding et al. 2017 |
| British Virgin Islands | 32,876 | CIA 2017b *^b^* | 386,049 | CTO 2015 | 890.2 | Undata 2017b; OECD 2018 | 0.220 | Spalding et al. 2017 |
| Cayman Islands | 59,172 | World Bank 2017a | 382,816 | CTO 2015 | 2485.0 | ESO 2014 | 0.118 | Spalding et al. 2017 |
| Cuba | 11,379,111 | World Bank 2017a | 3,001,958 | CTO 2015 | 74779.8 | World Bank 2017b | 0.004 | Spalding et al. 2017 |
| Curaçao | 155,872 | World Bank 2017a | 450,953 | CTO 2015 | 3183.2 | CBCSM 2018;  CBS 2018 | 0.036 | Spalding et al. 2017 |
| Dominica | 72,341 | World Bank 2017a | 81,472 | CTO 2015 |  |  | 0.016 | Linear model |
| Dominican Republic | 10,405,943 | World Bank 2017a | 5,141,377 | CTO 2015 | 63987.2 | World Bank 2017b | 0.008 | Spalding et al. 2017 |
| Grenada | 106,349 | World Bank 2017a | 133,521 | CTO 2015 | 839.7 | World Bank 2017b | 0.028 | Spalding et al. 2017 |
| Guadeloupe | 398,186 | Couillaud 2017a *^c^* | 487,000 | IEDOM 2015 | 10247.5 | IEDOM 2014a; INSEE 2017a; IRS 2017 | 0.009 | Spalding et al. 2017 |
| Haiti | 10,572,029 | World Bank 2017a | 465,174 | CTO 2015 | 8006.6 | World Bank 2017b | 0.002 | Spalding et al. 2017 |
| Jamaica | 2,721,252 | World Bank 2017a | 2,080,181 | CTO 2015 | 14320.1 | World Bank 2017b | 0.023 | Spalding et al. 2017 |
| Martinique | 383,910 | Couillaud 2017b | 489,561 | CTO 2015 | 10642.1 | IEDOM 2014b; INSEE 2017b; IRS 2017 | 0.008 | Spalding et al. 2017 |
| Montserrat | 5,000 | World Bank 2017a | 8,804 | CTO 2015 |  |  | 0.025 | Linear model |
| Puerto Rico | 3,548,397 | World Bank 2017a | 1,688,472 | CTO 2015 | 99679.9 | World Bank 2017b | 0.007 | Spalding et al. 2017 |
| Saba | 1,846 | CBS 2017 | 20,500 | CTO 2015 |  |  | 0.155 | Linear model |
| St. Barthelemy | 9,240 | Couillaud 2017a *^d^* | 281,272 | IEDOM 2011 |  |  | 0.425 | Linear model |
| St. Eustatius | 4,020 | CBS 2017 | 14,600 | CBS 2015 |  |  | 0.051 | Linear model |
| St. Kitts & Nevis | 54,944 | World Bank 2017a | 104,730 | CTO 2015 | 814.3 | World Bank 2017b | 0.020 | Spalding et al. 2017 |
| St. Lucia | 183,645 | World Bank 2017a | 338,158 | CTO 2015 | 1311.5 | World Bank 2017b | 0.043 | Spalding et al. 2017 |
| St. Maarten | 37,664 | World Bank 2017a | 499,920 | CTO 2015 | 1016.9 | CBCSM 2018 | 0.115 | Spalding et al. 2017 |
| St. Martin | 34,757 | Couillaud 2017a *^e^* | 201,219 | IEDOM 2013 |  |  | 0.081 | Linear model |
| St. Vincent & the Grenadines | 109,360 | World Bank 2017a | 70,713 | CTO 2015 | 748.2 | World Bank 2017b | 0.033 | Spalding et al. 2017 |
| Trinidad & Tobago | 1,354,483 | World Bank 2017a | 412,537 | CTO 2015 | 24542.5 | World Bank 2017b | 0.002 | Spalding et al. 2017 |
| Turks & Caicos | 33,740 | World Bank 2017a | 368,164 | CTO 2015 | 749.7 | UNdata 2017e; OECD 2018 | 0.130 | Spalding et al. 2017 |
| US Virgin Islands | 104,170 | World Bank 2017a | 730,367 | CTO 2015 | 3408.1 | World Bank 2017b | 0.081 | Spalding et al. 2017 |

*^a^* Population growth data from UNdata 2017a; *^b^* population data from UNdata 2017b; *^c^* population growth data from UNdata

2017c; *^d^* population growth data from UNdata 2017d; *^e^* population growth data from UNdata 2017e

##

### Socioeconomic Adaptive Capacity

Indicators of adaptive capacity include variables related to governance structures and social institutions, learning, economic assets, infrastructure, and social capacity (202,203). We calculated socioeconomic adaptive capacity as a function of adult literacy rate (*Lit*), NGO presence (*NGO*), ecological (*M_E_*) and fisheries (*M_F_*) monitoring programs, climate change plans (*Plan_CC_*), adaptive fisheries management plans (*Plan_AF_*), and the GINI index of economic inequality (*GINI*), using equation (S8):

$$SAC=0.25*Lit+0.25*NGO+0.25*\frac{M_{E}+M_{F}}{2}+0.25*\frac{{Plan}_{CC}+ {Plan}_{AF}+GINI}{3} (S8)$$

Individual learning and the potential for livelihood diversification*:* Learning at the individual level allows people to access and process information about environmental changes and opportunities for adaptation (203), while switching between livelihood strategies allows individuals to buffer their incomes against varying environmental and economic conditions (204). We used adult literacy rates as an indicator of both individual learning and the potential for livelihood diversification at the island level, assuming that literate individuals are better able to access new information and to transition between livelihood strategies when given the opportunity (205–207). We differentiate between individual learning, or learning as a process undertaken by individual people, and institutional learning at broader social scales, which we measure through the presence of ecological and fisheries monitoring programs (see section on *Ability to detect environmental changes*).

There is no comprehensive database of adult literacy across the Caribbean. We compiled data from several sources (Table S12), prioritizing recent data from global datasets that defined adult literacy rates as the proportion of people over the age of 15 who can read and write. When possible, we used data from the World Bank from the most recent year within the last decade (208). For islands that did not provide this data to the World Bank, we used 2015 data from the UN Economic Commission for Latin America and the Caribbean’s statistical database (209), the most recent available data from 2005-2010 from a region-wide report from the Pan American Health Organization (PAHO) (210), or 2001 data from a UN report on Millennium Development Goals (211). For the remaining five islands, we used data from undetermined years from regional reports (134,212). Some of the datasets did not include definitions of adult literacy rates or used lower bounds other than the age of 15 to define adults.

**Table S12.** Literacy rates across the Caribbean.

| Island | Literacy Rate | Data Year | Demographic measured | Source |
| --- | --- | --- | --- | --- |
| Anguilla | 0.98 | 2010 | Unknown | PAHO 2012a |
| Antigua & Barbuda | 0.99 | 2014 | Adult (> 15) | World Bank 2017d |
| Aruba | 0.98 | 2015 | Adult (> 15) | World Bank 2017d |
| Bahamas | 0.96 | 2005 | Adult (lower bound unknown) | PAHO 2012a |
| Barbados | 1.00 | 2010 | Adult (> 15) | PAHO 2012a*^a^* |
| Bonaire*^b^* | 0.96 | 2009 | Adult (lower bound unknown) | PAHO 2012a |
| British Virgin Islands | 0.98 | 2010 | Adult (> 15) | PAHO 2012a |
| Cayman Islands | 0.99 | 2007 | Adult (> 15) | World Bank 2017d |
| Cuba | 1.00 | 2015 | Adult (> 15) | World Bank 2017d |
| Curaçao *^b^* | 0.96 | 2009 | Adult (lower bound unknown) | PAHO 2012a |
| Dominica | 0.86 | 2008 | Unknown | PAHO 2012a |
| Dominican Republic | 0.92 | 2015 | Adult (> 15) | World Bank 2017d |
| Grenada | 0.97 | 2005 | Adult (lower bound unknown) | PAHO 2012a |
| Guadeloupe | 0.96 | 2015 | Adult (> 15) | CEPALSTAT 2016 |
| Haiti | 0.61 | 2015 | Adult (> 15) | World Bank 2017d |
| Jamaica | 0.88 | 2015 | Adult (> 15) | World Bank 2017d |
| Martinique | 0.97 | 2015 | Adult (> 15) | CEPALSTAT 2016 |
| Montserrat | 0.96 | Unknown | Adult (> 10) | de Bettencourt & Imminga-Berends 2015 |
| Puerto Rico | 0.93 | 2015 | Adult (> 15) | World Bank 2017d |
| Saba*^b^* | 0.96 | 2009 | Adult (lower bound unknown) | PAHO 2012a |
| St. Barthelemy | 1.00 | Unknown | Unknown | de Bettencourt & Imminga-Berends 2015 |
| St. Eustatius*^b^* | 0.96 | 2009 | Adult (lower bound unknown) | PAHO 2012a |
| St. Kitts & Nevis | 0.97 | 2009 | Adult (> 15) | PAHO 2012a |
| St. Lucia | 0.90 | 2001 | Adult (> 15) | UNDP 2003 |
| St. Maarten*^b^* | 0.96 | 2009 | Adult (lower bound unknown) | PAHO 2012a |
| St. Martin | 0.96 | Unknown | Unknown | de Bettencourt & Imminga-Berends 2015 |
| St. Vincent & the Grenadines | 0.89 | 2001 | Adult (> 15) | UNDP 2003 |
| Trinidad & Tobago | 0.99 | 2015 | Adult (> 15) | World Bank 2017d |
| Turks & Caicos | 0.98 | Unknown | Unknown | De Bettencourt & Imminga-Berends 2015 |
| US Virgin Islands*^c^* | 0.93 | Unknown | Adult (> 15) | Gaible 2009(212) |

*^a^* Data from PAHO 2012, with literacy definition from SALISES 2012(213). *^b^* Literacy rate averaged across the former Netherlands Antilles (Bonaire, Curaçao, Saba, St. Eustatius, and St. Maarten). *^c^* We report the median of the range reported (90-95%).

Social capacity: In the absence of a standardized source of information on local, national, regional, and international NGOs present on each island, we used Forbes’ list of the conservation NGOs with the largest budgets to identify the five largest international conservation NGOs: Conservation International, Environmental Defense Fund, The Nature Conservancy, Wildlife Conservation Society, and World Wildlife Fund (214). For each of the NGOs, we searched their websites for a physical presence on each island and a marine focus, as evidenced by offices or dedicated staff members. We did not count NGOs that were involved in projects without establishing a physical presence. We then divided the number of NGOs present on each island by the maximum number of NGOs that could be present on any island (n = 5; Table S13).

We expect that other factors, such as the presence of local or regional NGOs, fisheries cooperatives, and other civil society organizations and forms of self-governance, as well as community characteristics such as levels of trust, are likely to drive important local-level variation in social capacity. Nevertheless, in the absence of comprehensive or comparable island-level data on these indicators across the region, we used the presence of large, international conservation NGOs as a proxy for island-level social capacity.

**Table S13.** Presence of the five largest international environmental NGOs.

| Island | Organizations present |
| --- | --- |
| Anguilla | None |
| Antigua & Barbuda | None |
| Aruba | None |
| Bahamas | The Nature Conservancy |
| Barbados | None |
| Bonaire | None |
| British Virgin Islands | None |
| Cayman Islands | None |
| Cuba | None |
| Curaçao | None |
| Dominica | None |
| Dominican Republic | The Nature Conservancy |
| Grenada | None |
| Guadeloupe | None |
| Haiti | None |
| Jamaica | The Nature Conservancy |
| Martinique | None |
| Montserrat | None |
| Puerto Rico | None |
| Saba | None |
| St. Barthelemy | None |
| St. Eustatius | None |
| St. Kitts & Nevis | None |
| St. Lucia | None |
| St. Maarten | None |
| St. Martin | None |
| St. Vincent & the Grenadines | None |
| Trinidad & Tobago | None |
| Turks & Caicos | None |
| US Virgin Islands | The Nature Conservancy |

#### Ability to detect environmental changes

We assumed that islands with active environmental monitoring programs would be better able to detect ecological changes related to coral bleaching; earlier and more accurate detection of change facilitates adaptation (203). We measured this indicator through the presence of active ecological monitoring programs or research stations and fisheries monitoring programs.

Ecological monitoring and research stations: We coded the presence of ecological monitoring programs as binary, presence/absence data. We restricted ecological monitoring programs to those that had collected at least three years of standardized data since 2000. To find evidence of ecological monitoring, we first reviewed two reports that synthesized monitoring data region-wide: Jackson *et al*. (2014) (83) and Wilkinson & Souter (2008) (215). Where Wilkinson & Souter (2008) referenced government monitoring, we verified that this monitoring conformed to our guidelines by checking government reports (*e.g.*, J. C. Gumbs 2012 (216)). Nine islands had no evidence of sustained, recent ecological monitoring in these reports. To verify that these islands indeed had no such ecological monitoring efforts, we also looked at the data records in Reef Check, identifying islands with at least three years of surveys at the same reef sites (Table S14) (217).

Fisheries monitoring programs: We defined the presence of a fisheries monitoring program as the collection of fishery catch or landings data by a government agency or another organization at a frequency and intensity sufficient to make regularly-updated assessments of fisheries performance for at least one fishery (*e.g.*, the lobster fishery). Islands that have had irregular, inconsistent, or one-off landings surveys, such as Curaçao, were coded as not having a fishery monitoring program. We coded the presence of fisheries monitoring programs as binary, presence/absence data. We searched for evidence of fisheries monitoring programs through reports by national agencies (38), the FAO (218,219) and other intergovernmental entities (134,220), as well as in catch reconstruction reports by the Sea Around Us (49) (Table S14).

**Table S14.** Presence of ecological and fishery monitoring programs.

| Island | Presence of ecological monitoring | Source | Presence of fisheries monitoring | Source |
| --- | --- | --- | --- | --- |
| Anguilla | 1 | Wilkinson 2008; J. Gumbs 2012 | 1 | J. Gumbs 2012; Gumbs et al. 2015 |
| Antigua & Barbuda | 0 | Reef Check 2017 | 1 | FAO, 2007 |
| Aruba | 0 | Reef Check 2017 | 0 | Boekhoudt 2015; Pauly et al. 2015 |
| Bahamas | 1 | Jackson et al. 2014 | 1 | Gittens, 2007 |
| Barbados | 1 | Jackson et al. 2014 | 1 | McConney 2011; Mohammed et al. 2003 |
| Bonaire | 1 | Jackson et al. 2014 | 0 | de Bettencourt & Imminga-Berends 2015; Schep et al. 2012 |
| British Virgin Islands | 1 | Jackson et al. 2014 | 1 | Ramdeen et al. 2014a |
| Cayman Islands | 1 | Jackson et al. 2014 | 0 | de Bettencourt & Imminga-Berends 2015 |
| Cuba | 1 | Jackson et al. 2014 | 1 | Valle et al. 2011 |
| Curaçao | 1 | Jackson et al. 2014 | 0 | Lindop et al. 2015 |
| Dominica | 1 | Jackson et al. 2014 | 1 | Ramdeen et al. 2014b |
| Dominican Republic | 1 | Jackson et al. 2014 | 1 | van der Meer et al. 2014 |
| Grenada | 1 | Jackson et al. 2014 | 1 | Baldeo 2011 |
| Guadeloupe | 1 | Jackson et al. 2014 | 1 | Leblond et al. 2008 |
| Haiti | 0 | Reef Check 2017 | 0 | Ramdeen et al. 2012a |
| Jamaica | 1 | Jackson et al. 2014 | 1 | Murray 2007 |
| Martinique | 1 | Jackson et al. 2014 | 1 | Leblond et al. 2008 |
| Montserrat | 1 | Reef Check 2017 | 1 | Ramdeen et al. 2012b |
| Puerto Rico | 1 | Jackson et al. 2014 | 1 | Valle-Esquivel et al. 2011 |
| Saba | 0 | Reef Check 2017 | 0 | Lindop et al. 2015 |
| St. Barthelemy | 1 | Jackson et al. 2014 | 0 | Bultel et al. 2015 |
| St. Eustatius | 0 | Reef Check 2017 | 0 | Lindop et al. 2015 |
| St. Kitts & Nevis | 0 | Reef Check 2017 | 1 | Ramdeen et al. 2013 |
| St. Lucia | 1 | Jackson et al. 2014 | 1 | George et al. 2015 |
| St. Maarten | 0 | Reef Check 2017 | 0 | Lindop et al. 2015 |
| St. Martin | 1 | Reef Check 2017 | 0 | Bultel et al. 2015 |
| St. Vincent & the Grenadines | 1 | Jackson et al. 2014 | 1 | Straker 2007 |
| Trinidad & Tobago | 1 | Jackson et al. 2014 | 1 | Mohammed et al. 2011 |
| Turks & Caicos | 1 | Jackson et al. 2014 | 1 | Lockhart 2007 |
| US Virgin Islands | 1 | Jackson et al. 2014 | 1 | Rothenberger et al. 2008 |

###

#### Ability to respond to environmental changes

We assumed that islands with experience implementing adaptive environmental management would be better positioned to adapt to increased coral bleaching (241). To determine whether an island had experience with adaptive environmental management, we looked for the presence of climate change and adaptive fisheries management plans.

####

*Climate change plan*: We identified climate change policies through the FAO’s database of natural resource management legislation (FAOLEX (242)) and the Climate Change Laws of the World (CCLW) database (243). We used FAOLEX’s advanced search feature to search for the key words “climate change” in each island; we searched the CCLW database by island (Table S15). For Dutch islands, we also reviewed the island-level policies and regulations compiled by the Dutch Caribbean Nature Alliance (244). We restricted our search to plans that included adaptation, not merely plans for reducing greenhouse gas emissions. Similarly, we excluded plans that included non-specific indications of future intentions to integrate adaptation into policies (*e.g.*, Phillips & Emanuel 2005 (245)), as well as guidance documents that made suggestions for climate change adaptation but did not represent official policies (*e.g.*, Schill *et al*. 2014 (246); PRCCC 2015 (247)). For overseas territories, we looked for territory-level adaptation plans, rather than inclusion in plans and policies adopted by their associated mainland countries. Finally, we excluded adaptation plans that were identified as unimplemented drafts (*e.g.*, Hodge *et al*. 2011 (248)).

*Adaptive fisheries management*: We defined adaptive fisheries management as the presence of a fisheries management plan or regulation passed within the last 10 years (2007-2017). We recognize that adaptive environmental management is a more complicated concept than merely maintaining an updated plan (249,250) but used the 10-year cut-off as a simple proxy, rather than making potentially subjective assessments based on the terminology included in different plans. We used the FAOLEX database to identify recent fisheries-related regulations and plans (242), restricting our search to rules in the “Fisheries” category and excluding rules for freshwater fisheries, as in the case of Dominica. We did not include reprints of previously-established regulations. For islands with no recent fisheries management regulations in FAOLEX, we also searched island-level reports and fisheries agencies, excluding draft regulations and fisheries development plans that do not include actual regulations or rules (Table S15).

#### **Table S15.** Presence of climate change adaptation plans and adaptive fisheries management.

| Island | Climate change adaptation plan | Source | Adaptive fisheries management | Source |
| --- | --- | --- | --- | --- |
| Anguilla | 0 | Hodge et al. 2011 | 0 | FAO 2017a; Gumbs et al. 2015 |
| Antigua & Barbuda | 1 | Government of Antigua & Barbuda 2015 | 1 | FAO 2017a; Government of Antigua & Barbuda 2014 |
| Aruba | 0 | DCNA 2014 | 0 | FAO 2017a |
| Bahamas | 1 | NCCC & BESTC 2005 | 1 | FAO 2017a; Government of the Bahamas 2012 |
| Barbados | 1 | Rawlins-Bentham 2012 | 0 | FAO 2017a; Government of Barbados 2012 |
| Bonaire | 0 | DCNA 2014 | 1 | Arnold 2015; FAO 2017a |
| British Virgin Islands | 1 | Penn 2011 | 1 | FAO 2017a; Government of the British Virgin Islands 2014 |
| Cayman Islands | 1 | NCCC 2011 | 1 | FAO 2017a; Government of the Cayman Islands 2011 |
| Cuba | 1 | Martinez-Hernandez & Meneses 2017 | 1 | FAO 2017a; Government of Cuba 2016 |
| Curaçao | 0 | HEN 2014 | 1 | FAO 2017a; Vermeij & Chamberland 2009 |
| Dominica | 1 | Homer 2009 | 0 | FAO 2017a; SOFRECO 2012 |
| Dominican Republic | 1 | CNCCMDL 2012 | 1 | FAO 2017a; Government of the Dominican Republic 2015 |
| Grenada | 1 | Belfon 2011 | 1 | FAO 2017a; Government of Grenada 2014 |
| Guadeloupe | 1 | ADEME n.d. | 0 | FAO 2017a |
| Haiti | 1 | Government of Haiti 2006 | 0 | FAO 2017a |
| Jamaica | 1 | MWLECC 2013 | 1 | FAO 2017a; Government of Jamaica 2015 |
| Martinique | 0 | CLIMPACT 2012 | 0 | FAO 2017a |
| Montserrat | 0 | Phillips & Emanuel 2005 | 0 | FAO 2017a |
| Puerto Rico | 0 | PRCCC 2015 | 1 | FAO 2017a |
| Saba | 0 | DCNA 2014 | 0 | FAO 2017a |
| St. Barthelemy | 0 | CLIMPACT 2012 | 1 | FAO 2017a; Government of St. Barthelemy 2016 |
| St. Eustatius | 0 | DCNA 2014 | 0 | FAO 2017a; STENAPA 2008 |
| St. Kitts & Nevis | 0 | Droiterre Inc. & Associates 2015; Island Planning Services n.d. | 1 | FAO 2017a; Government of St. Kitts & Nevis n.d.; MAMRC 2015 |
| St. Lucia | 1 | PDEH 2003 | 1 | FAO 2017a; Government of St. Lucia 2011 |
| St. Maarten | 0 | DCNA 2014 | 1 | FAO 2017a; Government of Sint Maarten 2011 |
| St. Martin | 0 | CLIMPACT 2012 | 0 | FAO 2017a |
| St. Vincent & the Grenadines | 1 | Government of St. Vincent & the Grenadines n.d. | 0 | FAO 2017a; Government of St. Vincent & the Grenadines 2017 |
| Trinidad & Tobago | 1 | Government of Trinidad & Tobago 2011 | 0 | FAO 2017a; Mohammed 2017 |
| Turks & Caicos | 1 | Gordon 2013 | 1 | FAO 2017a; Government of Turks & Caicos Islands 2009 |
| US Virgin Island | 1 | Government of the United States Virgin Islands 2015 | 1 | FAO 2017a |

*GINI index*: We used the GINI index as an indicator of the equity of distribution of economic resources on an island, an important factor for determining adaptive capacity (3,205,292). The GINI index ranges from 0 to 1, with 0 indicating complete income equality and higher values indicating more inequality. There is no single database with GINI index values for each island in the Caribbean; we compiled data on GINI indices from the World Bank and island-level assessments. Three islands (the Dominican Republic, Haiti, and Jamaica) had data points since 1995 in the World Bank’s dataset: for these three islands, we used the most recent World Bank GINI calculation (293). Next, we drew data from island-level poverty assessments commissioned in the past 10 years by the Caribbean Development Bank (294–299), followed by island-level poverty assessments commissioned by island governments, including older estimates when necessary (213,300–305). For the remaining islands, we used GINI index values that were either calculated by the Pan American Health Organization (210) or compiled in a book on Caribbean economic history (306). We were unable to find GINI index values for Bonaire, Saba, St. Barthelemy, St. Martin, St. Maarten, St. Eustatius, and the US Virgin Islands. We calculated the average GINI index for the Caribbean (0.415) and used this value as the estimated GINI index for the seven islands with missing data (Table S16). Because a higher GINI index indicates higher income inequality and thus less adaptive capacity, we inverted the GINI index before combining it with other indicators of adaptive capacity so that higher values would indicate higher adaptive capacity.

**Table S16.** GINI indices (prior to inversion).

| Island | GINI index | Year | Source |
| --- | --- | --- | --- |
| Anguilla | 0.39 | 2008-2009 | Kairi Consultants Ltd. 2009a |
| Antigua & Barbuda | 0.48 | 2005-2006 | Kairi Consultants Ltd. 2007 |
| Aruba | 0.40 | 2000 | Bulmer-Thomas 2012 |
| Bahamas | 0.44 | 2009 | PAHO 2012a |
| Barbados | 0.47 | 2010 | SALISES, 2012 |
| Bonaire | 0.42 |  | Caribbean average |
| British Virgin Islands | 0.23 | 2002 | Halcrow Group Ltd. 2003 |
| Cayman Islands | 0.40 | 2006-2007 | Kairi Consultants Ltd. 2008c |
| Cuba | 0.38 | 2004 | Bulmer-Thomas 2012 |
| Curaçao | 0.40 | 2011 | La Chispa 2014 |
| Dominica | 0.44 | 2008-2009 | Kairi Consultants Ltd. 2010 |
| Dominican Republic | 0.47 | 2013 | World Bank 2017e |
| Grenada | 0.37 | 2008 | Kairi Consultants Ltd. 2008a |
| Guadeloupe | 0.41 | 2009-2010 | Benhaddouche 2014 |
| Haiti | 0.61 | 2012 | World Bank 2017e |
| Jamaica | 0.45 | 2004 | World Bank 2017e |
| Martinique | 0.31 | 2006 | Bulmer-Thomas 2012 |
| Montserrat | 0.39 | 2008-2009 | Halcrow Group Ltd. 2012 |
| Puerto Rico | 0.53 | 2009 | Noss 2010 |
| Saba | 0.42 |  | Caribbean average |
| St. Barthelemy | 0.42 |  | Caribbean average |
| St. Eustatius | 0.42 |  | Caribbean average |
| St. Kitts & Nevis | 0.40 | 2007-2008 | Kairi Consultants Ltd. 2009b |
| St. Lucia | 0.42 | 2006 | PAHO 2012d |
| St. Maarten | 0.42 |  | Caribbean average |
| St. Martin | 0.42 |  | Caribbean average |
| St. Vincent & the Grenadines | 0.40 | 2007-2008 | Kairi Consultants Ltd. 2008b |
| Trinidad & Tobago | 0.39 | 2005 | Bulmer-Thomas 2012 |
| Turks & Caicos | 0.36 | 2012 | Halcrow Group Ltd. 2014 |
| US Virgin Islands | 0.42 |  | Caribbean average |

## C. Covariates

We compiled data on four covariates related to marine resource management and island-level governance: marine protected area coverage, fisheries regulations, Worldwide Governance Indicator score, and sovereignty.

Marine Protected Area (MPA) Coverage*:* We compiled a database of MPAs by identifying non-terrestrial, established protected areas in the World Database on Protected Areas (311), supplemented by additional MPAs in the Caribbean Marine Protected Area Management database (312), MPAtlas (313), Reefs at Risk Revisited (314), and the US MPA Inventory (315). For all the databases, we restricted our analyses to MPAs in the EEZs of the thirty islands in our analysis. We compiled a separate list of MPAs that were coded in the WDPA and MPAtlas as “Proposed,” but not yet “Designated” or “Inscribed.” We conducted a literature review to see whether the status of any of these MPAs had changed since the data were entered in the databases, and included all MPAs that had been officially designated as of August 2016.

Using ArcGIS, we calculated the percentage of each island’s coral reefs that are covered by MPAs by overlaying the MPA shapefiles for each island’s EEZ onto a GIS layer of the island’s coral reefs, based on the cropping used for calculating mean ecological exposure (6,316) (Table S17).

**Table S17.** The percentage of each island’s coral reefs that are covered by marine protected areas (MPAs).

| Island | MPA area (km^2^) | Coral area (km^2^) | Percent of coral reefs in MPAs |
| --- | --- | --- | --- |
| Anguilla | 0 | 24.3 | 0.0 |
| Antigua & Barbuda | 335 | 58.1 | 55.9 |
| Aruba | 0 | 65.0 | 0.0 |
| Bahamas | 27763 | 2195.3 | 9.0 |
| Barbados | 14 | 35.2 | 0.1 |
| Bonaire | 8 | 18.5 | 1.2 |
| British Virgin Islands | 3 | 139.9 | 0.3 |
| Cayman Islands | 93 | 130.1 | 35.1 |
| Cuba | 14445 | 2609.1 | 31.9 |
| Curaçao | 11 | 34.3 | 4.4 |
| Dominica | 4 | 15.5 | 0.2 |
| Dominican Republic | 2530 | 352.9 | 22.4 |
| Grenada | 230 | 47.1 | 36.7 |
| Guadeloupe | 1478 | 118.2 | 28.9 |
| Haiti | 3532 | 338.7 | 16.9 |
| Jamaica | 1856 | 402.1 | 16.0 |
| Martinique | 9 | 77.9 | 0.3 |
| Montserrat | 0 | 2.3 | 0.0 |
| Puerto Rico | 3228 | 165.0 | 17.6 |
| Saba | 10 | 178.9 | 0.0 |
| St. Barthelemy | 12 | 10.0 | 22.1 |
| St. Eustatius | 0 | 12.5 | 0.0 |
| St. Kitts & Nevis | 0 | 42.4 | 0.0 |
| St. Lucia | 423 | 29.5 | 49.5 |
| St. Maarten | 34 | 1.6 | 0.0 |
| St. Martin | 33 | 6.4 | 78.0 |
| St. Vincent & the Grenadines | 92 | 41.1 | 43.4 |
| Trinidad & Tobago | 11 | 31.1 | 11.8 |
| Turks & Caicos | 152 | 192.0 | 7.2 |
| US Virgin Islands | 22 | 35.4 | 4.4 |

Fisheries Regulations*:* We identified seven components of fisheries management that may influence social-ecological vulnerability by controlling fishing effort and reducing pressure on key species and life history stages: requirements for fishing permits or licenses, fishing gear restrictions (*e.g.*, minimum mesh sizes, bans on certain gears in certain locations, etc.), size restrictions, quotas or catch limits, the use of temporary or seasonal closures (including closed seasons for targeted species), complete fishing bans for specific species (excluding those for sea turtles, marine mammals, and sea birds), and protections for herbivorous target species. We compiled data on the presence or absence of each of these seven different fisheries regulation types using FAOLEX, government agency websites, island- and regional-level reports, and published literature (Table S18). We used pairwise chi-square tests to assess for independence between the different fisheries regulations and found that all were independent. We then calculated a fisheries regulation score for each island as the number of fisheries regulations present on the island, divided by the total number of regulations considered (n = 7).

**Table S18.** Fisheries regulations present in each island.

| Island | License/ permit required | Gear restrictions | Size restrictions | Quota or total allowable catch | Temporary/ seasonal closure | Species bans | Herbivore protection | Sources |
| --- | --- | --- | --- | --- | --- | --- | --- | --- |
| Anguilla | 1 | 1 | 1 | 0 | 1 | 0 | 0 | Government of Anguilla 2000; Hoggarth 2001; J. Gumbs 2012 |
| Antigua & Barbuda | 1 | 1 | 1 | 1 | 1 | 1 | 1 | Government of Antigua & Barbuda 2013; Government of Antigua & Barbuda 2014; Horsford 2015 |
| Aruba | 1 | 1 | 0 | 0 | 0 | 1 | 1 | Government of Aruba 1992; Government of Aruba 2001; Theile 2001; Boekhoudt 2015 |
| Bahamas | 1 | 1 | 1 | 1 | 1 | 0 | 0 | Gittens 2007; Government of the Bahamas 2010; Waugh et al. 2010; Government of the Bahamas 2012; Government of the Bahamas 2015 |
| Barbados | 1 | 1 | 1 | 0 | 1 | 0 | 1 | Government of Barbados 1995; Barbados Fisheries Division 2003; Mcmanus & Lacambra 2004; McConney 2011 |
| Bonaire | 0 | 1 | 1 | 0 | 0 | 0 | 0 | Government of Bonaire 1991; Meyer & MacRae 2006; Government of Bonaire 2010 |
| British Virgin Islands | 1 | 1 | 1 | 0 | 1 | 0 | 0 | Government of the British Virgin Islands 2003; Ramdeen et al. 2014a; Conservation & Fisheries Department 2015 |
| Cayman Islands | 1 | 1 | 1 | 1 | 1 | 1 | 1 | Cayman Islands Department of the Environment 2008; Turner et al. 2013; Government of the Cayman Islands 2014 |
| Cuba | 1 | 1 | 1 | 1 | 1 | 0 | 1 | Government of Grenada 1987; Baisre-Hernandez 2006; Valle et al. 2011 |
| Curaçao | 1 | 1 | 1 | 0 | 0 | 0 | 0 | Vermeij & Chamberland 2009 |
| Dominica | 1 | 1 | 1 | 0 | 1 | 0 | 0 | Government of Dominica 1987; Mcmanus & Lacambra 2004; SOFRECO 2012 |
| Dominican Republic | 1 | 1 | 1 | 0 | 1 | 0 | 1 | Mcmanus & Lacambra 2004; Herrera et al. 2011; Mateo 2015 |
| Grenada | 1 | 1 | 1 | 0 | 1 | 0 | 1 | Government of Grenada 1987; Mcmanus & Lacambra 2004; Baldeo 2011 |
| Guadeloupe | 1 | 1 | 1 | 0 | 1 | 0 | 1 | Chakalall 1995; Bouchon et al. 2008 |
| Haiti | 1 | 1 | 1 | 0 | 1 | 0 | 0 | Government of Haiti 1978; Creary et al. 2008; UNEP-WCMC 2015 |
| Jamaica | 1 | 1 | 1 | 1 | 1 | 0 | 0 | Government of Jamaica 1976; Mcmanus & Lacambra 2004; Aiken et al. 2006; Murray 2007 |
| Martinique | 1 | 1 | 1 | 1 | 1 | 1 | 1 | Iborra Martin 2007 |
| Montserrat | 1 | 0 | 1 | 0 | 0 | 0 | 0 | Government of Montserrat 2002 |
| Puerto Rico | 1 | 1 | 1 | 1 | 1 | 1 | 0 | Mcmanus & Lacambra 2004; Government of Puerto Rico 2004; Valle-Esquivel et al. 2011 |
| Saba | 1 | 1 | 1 | 0 | 0 | 0 | 0 | Dilrosun 2000; Lundvall 2008; Toller & Lundvall 2008 |
| St. Barthelemy | 1 | 1 | 1 | 0 | 1 | 1 | 1 | Diaz & Cuzange 2009; Government of St. Barthelemy 2016 |
| St. Martin | 1 | 1 | 1 | 0 | 1 | 1 | 1 | Diaz & Cuzange 2009 |
| St. Eustatius | 1 | 1 | 1 | 1 | 0 | 0 | 0 | DCNA 2007; STENAPA 2008 |
| St. Maarten | 0 | 1 | 1 | 0 | 0 | 1 | 0 | DCNA 2006 |
| St. Kitts & Nevis | 1 | 1 | 1 | 0 | 1 | 0 | 0 | Government of St. Kitts & Nevis 1984; Government of St. Kitts & Nevis 1995; Mcmanus & Lacambra 2004 |
| St. Lucia | 1 | 1 | 1 | 0 | 1 | 0 | 1 | De Beauville-Scott 2007; George et al. 2015; Joseph 2003; Mcmanus & Lacambra 2004 |
| St. Vincent & the Grenadines | 1 | 1 | 1 | 0 | 1 | 0 | 1 | Blackman & Mattai 2007; Mcmanus & Lacambra 2004 |
| Trinidad & Tobago | 1 | 1 | 1 | 0 | 1 | 0 | 0 | Government of Trinidad & Tobago 2014; Mohammed et al. 2011 |
| Turks & Caicos | 1 | 1 | 1 | 0 | 1 | 1 | 1 | Government of Turks & Caicos Islands 2009; Lockhart 2007a; Logan & Sealey 2013 |
| US Virgin Islands | 1 | 1 | 1 | 1 | 1 | 1 | 1 | DPNR 2012; Mcmanus & Lacambra 2004 |

Governance Characteristics*:* We considered the sovereign status of each island (whether it is an independent nation or an overseas territory) and its Worldwide Governance Indicator (WGI) score. The WGI score reflects six dimensions of governance: voice and accountability, political stability and the absence of violence, government effectiveness, regulatory quality, rule of law, and control of corruption (364). All indicators range from -2.5 to 2.5, with higher values indicating better governance outcomes.

The WGI database includes scores from 2013 for 24 of the 30 islands we analyzed, with the five islands that formerly formed the Netherlands Antilles grouped as one entity. We assumed that all five component islands (Curaçao, Bonaire, Saba, St. Eustatius, and St. Maarten) had the same WGI scores in 2013, although these scores have likely diverged as each island’s governance system has evolved since the break-up of the Netherlands Antilles. The WGI database did not include estimated scores for three British territories (British Virgin Islands, Montserrat, and Turks & Caicos Islands) and three French territories (Guadeloupe, St. Barthelemy, and St. Martin). We excluded these six islands from analyses linking vulnerability to governance indicators.

We calculated the Pearson’s correlation coefficients for all pair-wise sets of WGI indicators and found that all pairs were significantly correlated, with correlation coefficients ranging from 0.59-0.93, indicating moderate to strong positive correlations. We thus used the average WGI score across all six indicators for further analyses (Table S19).

**Table S19.** Governance characteristics, including sovereignty status and the 2013 estimates of the six Worldwide Governance Indicators. For sovereignty status, “1” indicates that the island is an independent state; “0” indicates that it is a territory. Worldwide Governance Indicator data from Kaufmann & Kraay (2016).

| Island | Sovereign state | Worldwide Governance Indicators (2013) | | | | | | |
| --- | --- | --- | --- | --- | --- | --- | --- | --- |
|  |  | Voice & accountability | Political stability | Government effectiveness | Regulatory quality | Rule of law | Control of corruption | Average score |
| Anguilla | 0 | 1.05 | 1.59 | 1.54 | 1.32 | 1.41 | 1.3 | 1.37 |
| Antigua & Barbuda | 1 | 0.65 | 0.98 | 0.49 | 0.61 | 0.87 | 1.3 | 0.82 |
| Aruba | 0 | 1.26 | 1.34 | 1.22 | 1.45 | 1.31 | 1.14 | 1.29 |
| Bahamas | 1 | 0.91 | 1.12 | 0.87 | 0.17 | 0.61 | 1.37 | 0.84 |
| Barbados | 1 | 1.19 | 1.3 | 1.36 | 0.44 | 1.01 | 1.62 | 1.15 |
| St. Barthelemy | 0 | -- | -- | -- | -- | -- | -- | -- |
| Bonaire*^a^* | 0 | 0.4 | 0.72 | 0.75 | 0.85 | 0.89 | 0.83 | 0.74 |
| British Virgin Islands | 0 | -- | -- | -- | -- | -- | -- | -- |
| Cayman Islands | 0 | 0.52 | 1.06 | 1.22 | 1.12 | 0.9 | 1.37 | 1.03 |
| Cuba | 1 | -1.4 | 0.37 | -0.47 | -1.59 | -0.63 | -0.63 | -0.73 |
| Curaçao *^a^* | 0 | 0.4 | 0.72 | 0.75 | 0.85 | 0.89 | 0.83 | 0.74 |
| Dominica | 1 | 1 | 1.21 | 0.72 | 0.28 | 0.64 | 0.7 | 0.76 |
| Dominican Republic | 1 | 0.09 | 0.18 | -0.46 | -0.07 | -0.51 | -0.83 | -0.27 |
| St. Eustatius*^a^* | 0 | 0.4 | 0.72 | 0.75 | 0.85 | 0.89 | 0.83 | 0.74 |
| Grenada | 1 | 0.81 | 0.41 | 0.24 | 0.28 | 0.14 | 0.44 | 0.39 |
| Guadeloupe | 0 | -- | -- | -- | -- | -- | -- | -- |
| Haiti | 1 | -0.79 | -0.65 | -1.56 | -0.93 | -1.3 | -1.16 | -1.07 |
| Jamaica | 1 | 0.51 | 0.17 | 0.04 | 0.24 | -0.38 | -0.36 | 0.04 |
| St. Kitts & Nevis | 1 | 1.15 | 0.98 | 0.91 | 0.41 | 0.74 | 0.98 | 0.86 |
| St. Maarten*^a^* | 0 | 0.4 | 0.72 | 0.75 | 0.85 | 0.89 | 0.83 | 0.74 |
| Martinique | 0 | 0.62 | 0.68 | 0.75 | 0.85 | 0.89 | 0.83 | 0.77 |
| Montserrat | 0 | -- | -- | -- | -- | -- | -- | -- |
| Puerto Rico | 0 | 0.66 | 0.25 | 0.37 | 0.85 | 0.69 | 0.51 | 0.56 |
| Saba*^a^* | 0 | 0.4 | 0.72 | 0.75 | 0.85 | 0.89 | 0.83 | 0.74 |
| St. Lucia | 1 | 1.14 | 0.87 | 0.73 | 0.29 | 0.68 | 1.07 | 0.80 |
| St. Martin | 0 | -- | -- | -- | -- | -- | -- | -- |
| Trinidad & Tobago | 1 | 0.45 | 0.1 | 0.37 | 0.26 | -0.21 | -0.34 | 0.11 |
| Turks & Caicos | 0 | -- | -- | -- | -- | -- | -- | -- |
| US Virgin Islands | 0 | 0.83 | 0.96 | 1.28 | 0.61 | 0.89 | 0.83 | 0.9 |
| St. Vincent & the Grenadines | 1 | 1.05 | 0.87 | 0.91 | 0.32 | 0.87 | 0.98 | 0.83 |

*^a^* WGI data from the Netherlands Antilles.

# II. Analyses

## A. Spatial Autocorrelation

We assessed the spatial autocorrelation of each component of vulnerability using Moran’s *I* tests based on the centroid of each island. We detected significant spatial autocorrelation in ecological exposure, ecological recovery potential, socioeconomic adaptive capacity, and composite social-ecological vulnerability. There was also spatial autocorrelation in coral and reef fish species richness, coral taxon sensitivity, *Diadema* density, and coral cover (Table S20).

**Table S20.** Spatial autocorrelation in components and individual continuous variables of social-ecological vulnerability. Asterisks indicate significant p-values (* = p ≤ 0.05, ** = p ≤ 0.01, *** = p ≤ 0.001).

| Category | Component or variable name | Moran’s I (observed) | Standard deviation | p-value |
| --- | --- | --- | --- | --- |
| Component | Ecological exposure | 0.12 | 0.06 | 0.006** |
|  | Ecological sensitivity | -0.02 | 0.06 | 0.835 |
|  | Ecological recovery potential | 0.11 | 0.06 | 0.011* |
|  | Socioeconomic exposure | 0.04 | 0.06 | 0.186 |
|  | Socioeconomic sensitivity | 0.04 | 0.05 | 0.159 |
|  | Socioeconomic adaptive capacity | 0.15 | 0.06 | 0.001*** |
|  | Social-ecological vulnerability | 0.19 | 0.06 | <<0.001*** |
| Variable | Coral species richness | 0.35 | 0.06 | <<0.001*** |
|  | Coral sensitivity | 0.18 | 0.05 | <<0.001*** |
|  | Reef fish species richness | 0.14 | 0.05 | 0.001*** |
|  | *Diadema* density | 0.14 | 0.05 | 0.001*** |
|  | Coral cover | 0.14 | 0.06 | 0.002** |
|  | Scaridae biomass | 0.00 | 0.05 | 0.520 |
|  | Target species sensitivity | -0.01 | 0.06 | 0.605 |
|  | Algal cover | -0.02 | 0.06 | 0.752 |

## B. Correlations in Components of Vulnerability

We calculated the Pearson’s correlation coefficients between the different components of social-ecological vulnerability (Table S21).

**Table S21.** Correlations between components of ecological and socioeconomic vulnerability. Asterisks indicate significant p-values (* = p ≤ 0.05, ** = p ≤ 0.01, *** = p ≤ 0.001).

| Realm | Component 1 | Component 2 | Pearson's correlation coefficient | p-value |
| --- | --- | --- | --- | --- |
| Ecological | Ecological Exposure | Ecological Sensitivity | -0.25 | 0.174 |
|  | Ecological Exposure | Ecological Recovery Potential | 0.21 | 0.255 |
|  | Ecological Sensitivity | Ecological Recovery Potential | -0.23 | 0.228 |
| Socioeconomic | Socioeconomic Exposure | Socioeconomic Sensitivity | -0.31 | 0.098 |
|  | Socioeconomic Exposure | Socioeconomic Adaptive Capacity | 0.09 | 0.644 |
|  | Socioeconomic Sensitivity | Socioeconomic Adaptive Capacity | -0.19 | 0.302 |
| Social-Ecological | Ecological Exposure | Socioeconomic Exposure | 0.61 | 0.000*** |
|  | Ecological Exposure | Socioeconomic Sensitivity | -0.52 | 0.003** |
|  | Ecological Exposure | Socioeconomic Adaptive Capacity | 0.22 | 0.246 |
|  | Ecological Sensitivity | Socioeconomic Exposure | 0.42 | 0.020* |
|  | Ecological Sensitivity | Socioeconomic Sensitivity | -0.03 | 0.863 |
|  | Ecological Sensitivity | Socioeconomic Adaptive Capacity | -0.10 | 0.610 |
|  | Ecological Recovery Potential | Socioeconomic Exposure | -0.48 | 0.008** |
|  | Ecological Recovery Potential | Socioeconomic Sensitivity | -0.28 | 0.133 |
|  | Ecological Recovery Potential | Socioeconomic Adaptive Capacity | 0.09 | 0.634 |

## C. Coefficients of Variation

We calculated the coefficients of variation for the different components of social-ecological vulnerability (Table S22) and for the individual variables comprising each component (Table S23).

**Table S22.** Coefficients of variation for each component of vulnerability.

| Realm | Component | Coefficient of Variation |
| --- | --- | --- |
| Ecological | Exposure | 15 |
|  | Sensitivity | 32 |
|  | Recovery Potential | 18 |
| Socioeconomic | Exposure | 8 |
|  | Sensitivity | 48 |
|  | Adaptive Capacity | 26 |

**Table S23.** Coefficients of variation for each continuous variable. The average CV for ecological sensitivity variables was 39 (SD = 34) and the average CV for ecological recovery potential was 59 (SD = 52). The socioeconomic variables had higher average CVs: 115 for socioeconomic sensitivity (SD = 71) and 93 for socioeconomic adaptive capacity (SD = 144).

| Realm | Component | Variable | Coefficient of Variation |
| --- | --- | --- | --- |
| Ecological | Exposure | Stress score | 15 |
|  | Sensitivity | Coral sensitivity | 15 |
|  |  | Target species sensitivity | 63 |
|  | Recovery Potential | Coral cover | 57 |
|  |  | Algal cover | 25 |
|  |  | Coral species richness | 34 |
|  |  | Fish species richness | 16 |
|  |  | Scaridae biomass | 66 |
|  |  | *Diadema* density | 157 |
| Socioeconomic | Sensitivity | Proportion of small-scale fishers | 130 |
|  |  | Proportion of fish consumed domestically | 18 |
|  |  | Reef fisheries as percentage of GDP | 188 |
|  |  | Reef tourism as percentage of GDP | 125 |
|  | Adaptive Capacity | Literacy rate | 8 |
|  |  | NGO score | 259 |
|  |  | GINI index | 11 |

## D. Vulnerability Rankings

**Table S24.** Overall social-ecological vulnerability rankings of the 30 islands from least to most vulnerable (first column) and changes in rankings, relative to overall ranking, for the different components of vulnerability (subsequent columns). Exposure and Sensitivity scores are ranked in ascending order, from least exposed/sensitive to most, while Recovery Potential and Adaptive Capacity are ranked from highest to lowest scores so that for all columns, a ranking of 1 indicates lower vulnerability and a ranking of 30 indicates higher vulnerability. Negative numbers indicate that an island is more vulnerable in the component rankings than it is overall; positive numbers indicate that an island is less vulnerable in component rankings than it is overall. Islands that are not sovereign states (i.e., overseas territories) are indicated with an asterisk.

| *Social-Ecological Vulnerability* | *Ecological* | | | *Socioeconomic* | | |
| --- | --- | --- | --- | --- | --- | --- |
|  | Exposure (+) | Sensitivity (+) | Recovery Potential (-) | Exposure (+) | Sensitivity (+) | Adaptive Capacity  (-) |
| 1. Dominican Republic | -23 | -27 | -10 | -25 | -2 | -2 |
| 2. Cuba | -25 | -15 | -6 | -22 | -2 | -4 |
| 3. Jamaica | -11 | -22 | -11 | -16 | -7 | -1 |
| 4. Bahamas | 0 | -25 | -16 | -14 | -13 | 3 |
| 5. Grenada | -21 | 1 | -24 | -22 | 0 | -3 |
| 6. US Virgin Islands* | -5 | -9 | -16 | -5 | -15 | 4 |
| 7. St. Lucia | -16 | 2 | 3 | -2 | -9 | -2 |
| 8. Trinidad & Tobago | -22 | -5 | 5 | -17 | 6 | -2 |
| 9. Curacao* | -1 | -5 | 2 | 2 | 8 | -14 |
| 10. Dominica | -10 | 9 | 4 | 4 | -1 | -5 |
| 11. Guadeloupe* | -8 | -7 | 1 | -4 | 3 | -1 |
| 12. Puerto Rico* | -5 | -8 | 0 | -1 | 5 | -2 |
| 13. St. Vincent & the Grenadines | -9 | 6 | 0 | -3 | -2 | 0 |
| 14. Barbados | -14 | -2 | -2 | -15 | 0 | 3 |
| 15. Martinique* | -10 | 7 | 10 | 1 | 6 | -3 |
| 16. Montserrat* | 9 | 10 | -8 | 8 | 3 | -4 |
| 17. Cayman Islands* | 14 | 8 | -1 | 14 | -6 | 1 |
| 18. Turks & Caicos* | 9 | -6 | -7 | -4 | -8 | 11 |
| 19. Antigua & Barbuda | 14 | -2 | 2 | 9 | 1 | 2 |
| 20. St. Kitts & Nevis | 2 | 8 | -6 | -1 | 8 | -2 |
| 21. British Virgin Islands* | 5 | 2 | 6 | 4 | -8 | 16 |
| 22. Anguilla* | 9 | 0 | 1 | 2 | -3 | 3 |
| 23. St. Martin* | 2 | 21 | 0 | 11 | 1 | -2 |
| 24. Bonaire* | 16 | 13 | 23 | 23 | -4 | 0 |
| 25. Sint Eustatius* | 24 | -1 | 16 | 21 | 6 | -3.5 |
| 26. Aruba* | 11 | 16 | 24 | 24 | 6 | -1 |
| 27. Sint Maarten* | 21 | -3 | 8 | 4 | 3 | 1 |
| 28. Haiti | -1 | 5 | 0 | -2 | 22 | -2 |
| 29. St. Barthelemy* | 27 | 26 | 2 | 24 | -1 | 8 |
| 30. Saba* | 18 | 3 | 0 | 2 | 3 | 1.5 |

## E. Contribution of Variables to Vulnerability

We estimated each variable’s contribution to social-ecological vulnerability on each island by multiplying each variable’s value by the proportion of social-ecological vulnerability that it potentially accounts for, as described in equation (S1) (Table S25). Literacy rate has the highest mean contribution to social-ecological vulnerability (Figure S1). This is due in part to the high values of literacy rates in the region: literacy rate ranges from 0.61-1.0, while many other variables had lower maximum and minimum values. Most of the variables with the greatest contribution to social-ecological vulnerability are socioeconomic indicators. The variables with the highest standard deviation in their contribution to social-ecological vulnerability are the four variables related to monitoring and adaptive management (ecological monitoring, fisheries monitoring, climate change adaptation plan, and adaptive fisheries management) and the proportion of GDP from reef-based tourism. The monitoring and adaptive management variables have high standard deviations because they are binary variables with values of either 0 or 1; the high standard deviation in GDP from reef-based tourism reflects variability in the economic importance of this sector across islands.

**Table S25.** Proportion of social-ecological vulnerability attributable to each variable.

| Realm | Component | Variable | Contribution |
| --- | --- | --- | --- |
| Ecological | Exposure | Stress score | 0.111 |
|  | Sensitivity | Coral sensitivity | 0.056 |
|  |  | Target species sensitivity | 0.056 |
|  | Recovery Potential | Coral cover | 0.019 |
|  |  | Algal cover | 0.019 |
|  |  | Coral species richness | 0.019 |
|  |  | Fish species richness | 0.019 |
|  |  | Scaridae biomass | 0.019 |
|  |  | *Diadema* density | 0.019 |
| Socioeconomic | Sensitivity | Proportion of small-scale fishers | 0.056 |
|  |  | Proportion of fish consumed domestically | 0.056 |
|  |  | Reef fisheries as percentage of GDP | 0.056 |
|  |  | Reef tourism as percentage of GDP | 0.167 |
|  | Adaptive Capacity | Literacy rate | 0.083 |
|  |  | NGO score | 0.083 |
|  |  | Ecological monitoring | 0.042 |
|  |  | Fisheries monitoring | 0.042 |
|  |  | Climate change adaptation plan | 0.028 |
|  |  | Adaptive fisheries management | 0.028 |
|  |  | GINI index | 0.028 |

**Figure S1.** Variation in the contribution of each variable to the social-ecological vulnerability of each island. Bar length indicates the mean contribution across the 30 islands; error bars indicate the standard deviation (with the lower value of the mean minus standard deviation restricted to be no less than 0).


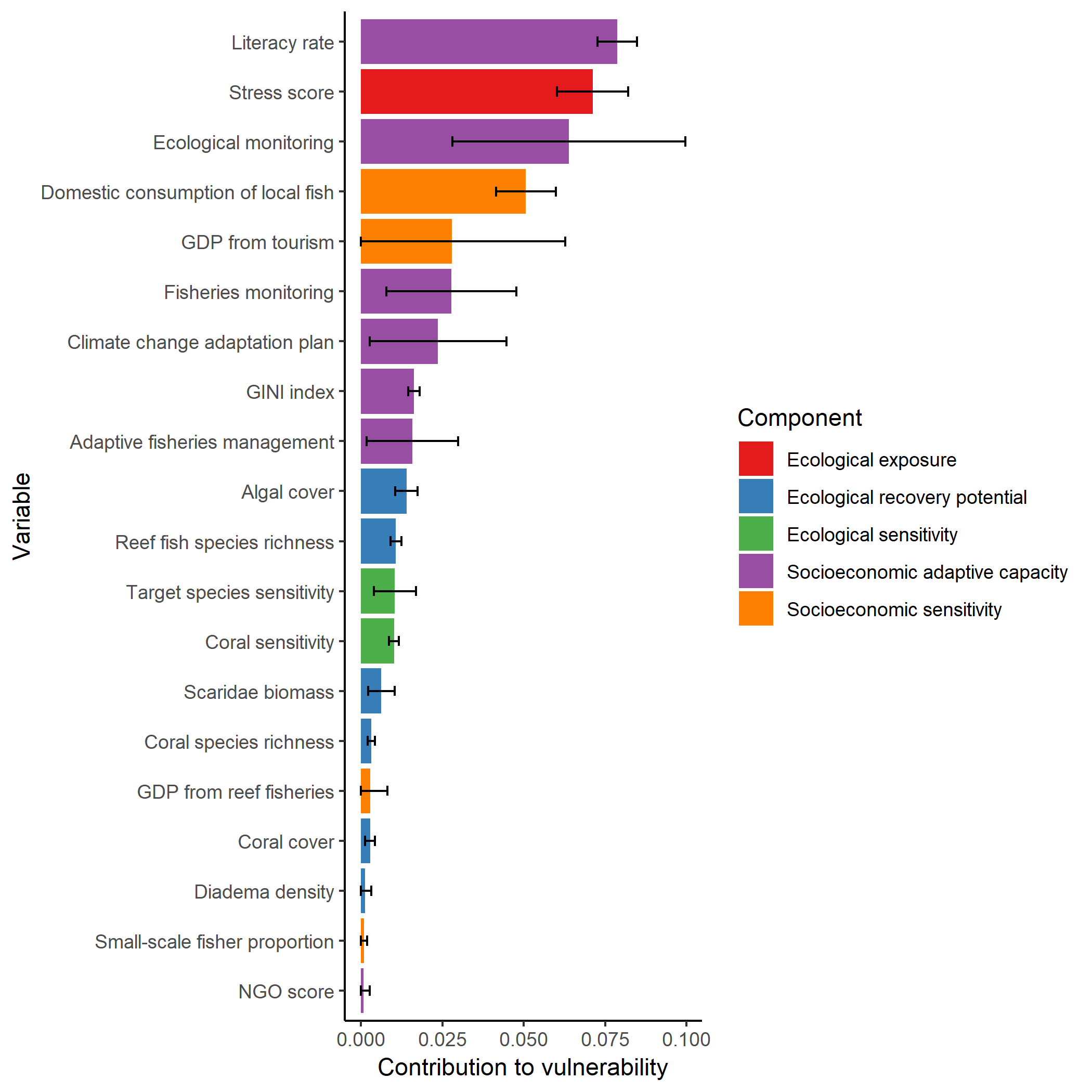


## F. Differences between Territories of Different Countries

Sample sizes are insufficient to determine whether the mainland country with which a territory is associated plays an important role in determining its social-ecological vulnerability to coral bleaching. However, here we adapt Figure 4 to show the potential for impact vs. recovery potential or vs. adaptive capacity for all of the territories, colored by their mainland country (Figure S2), providing a descriptive illustration of patterns in the territories’ vulnerability.

**Figure S2.** Potential ecological impacts (ecological exposure plus ecological sensitivity) versus recovery potential (top), and potential socioeconomic impacts (i.e., socioeconomic exposure and socioeconomic sensitivity) versus adaptive capacity (bottom) for the seventeen overseas territories in the study. As in Figure 4 in the main text, high potential impacts indicate a system with high exposure and/or sensitivity to bleaching, while high recovery potential or adaptive capacity indicates that a system is able to respond to changes. The territories are colored according to the mainland country with which they are associated.


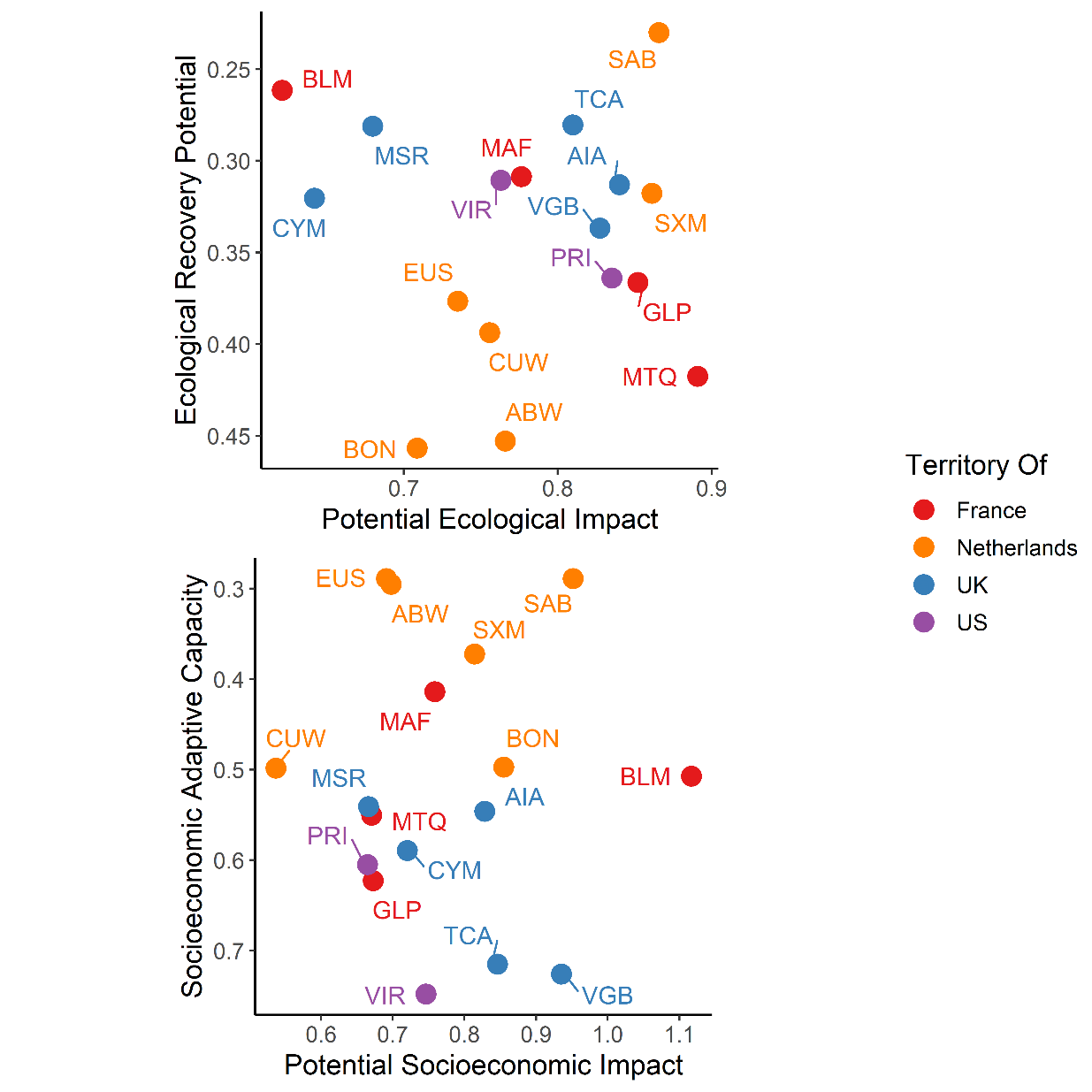


## G. Model Selection

We fit models linking our covariates (the percent of coral reefs protected, fisheries regulations, WGI score, and sovereignty) and the components of vulnerability using generalized linear models (GLMs) in R (365), using the packages *MASS*, *car*, *modEVA*, and *betareg* (366–369). We used a step-wise process for selecting significant covariates, choosing appropriate error structures, assessing model fit, and identifying the most parsimonious model for each component. For models using Gaussian and beta error structures, we assessed model fit using the Akaike information criterion (AIC); for quasibinomial error structures, we used D^2^ and model simplicity to select the best-fit model (when ANOVA tests revealed no significant difference in the amount of residual deviance and the explanatory variables were not statistically significant, we selected the simplest model). We then compared the performance of the best-fit model from each error structure using R^2^ and pseudo-R^2^ values.

For ecological exposure, we fit a model including the governance characteristics (WGI score and sovereignty) as potential covariates. We did not include the percentage of coral reefs covered by MPAs or the fisheries regulation score as potential covariates because we did not have a hypothesized mechanism linking these two variables to variation in watershed management factors, which are the aspects of ecological exposure that could potentially be influenced by island-scale governance characteristics. The ecological exposure model was limited to the 24 islands that had WGI data in the WGI database (364). For ecological sensitivity and recovery potential, we assessed the percentage of coral reefs covered by MPAs and the fisheries regulation score as potential covariates. We did not include the governance characteristics (WGI score and sovereignty) as covariates because we did not have a hypothesized mechanism linking these variables to these ecological components of vulnerability; this allowed us to include all 30 islands in the model. For the socioeconomic components of vulnerability, we modeled each component (exposure, sensitivity, and adaptive capacity) against all four covariates (percent of coral reefs protected by MPAs, fisheries regulation score, WGI score, and sovereignty), limiting our analysis to the 24 islands with WGI data. The best-fit models are presented in Table 1 of the main text.

Based on the results from the Shapiro-Wilk Normality test, we rejected the hypothesis that socioeconomic sensitivity (p < 0.01), adaptive capacity (p = 0.01), and social-ecological vulnerability (p = 0.01) come from populations with normal distributions. Based on visual inspection of qqplots and histograms, ecological exposure, ecological sensitivity, and socioeconomic exposure also appear to violate assumptions of normality. Ecological recovery potential does not appear to have a non-normal distribution.

We used Kruskal-Wallis tests to assess whether there were significant differences in the mean values of the individual variables and the components of social-ecological vulnerability for independent islands as compared to islands that are territories (Table S26).

**Table S26.** Comparison of mean variable, component (italicized), and covariates values for independent islands vs. territories, using Kruskal-Wallis tests. All variable values are unscaled. Asterisks indicate significant p-values (* = p ≤ 0.05, ** = p ≤ 0.01).

| Realm | Component | Variable | Mean (independent) | Mean (territory) | p-value |
| --- | --- | --- | --- | --- | --- |
| Ecological | Exposure | *Integrated stress score* | *0.70* | *0.59* | *<0.01*** |
|  | Sensitivity | Coral sensitivity | 0.19 | 0.17 | 0.01** |
|  |  | Target species sensitivity | 0.17 | 0.20 | 0.69 |
|  |  | *Ecological sensitivity* | *0.18* | *0.18* | *0.98* |
|  | Recovery Potential | Coral cover | 0.15 | 0.15 | 0.75 |
|  |  | Algal cover | 0.71 | 0.78 | 0.82 |
|  |  | Coral species richness | 49.69 | 47.59 | 0.72 |
|  |  | Fish species richness | 368.77 | 317.71 | 0.01** |
|  |  | Parrotfish biomass | 16.76 | 16.37 | 0.66 |
|  |  | Diadema density | 0.60 | 0.45 | 0.33 |
|  |  | *Ecological recovery potential* | *0.34* | *0.34* | *0.79* |
| Socioeconomic | Exposure | *Socioeconomic exposure* | *0.51* | *0.48* | *0.01*** |
|  | Sensitivity | Proportion of small-scale fishers | 0.01 | 0.02 | 0.85 |
|  |  | Proportion of fish consumed domestically | 0.88 | 0.93 | <0.01** |
|  |  | Reef fisheries as percentage of GDP | <0.01 | 0.01 | 0.57 |
|  |  | Reef tourism as percentage of GDP | 0.03 | 0.11 | <0.01** |
|  |  | *Socioeconomic sensitivity* | *0.18* | *0.30* | *<0.01*** |
|  | Adaptive Capacity | Literacy rate | 0.92 | 0.97 | 0.41 |
|  |  | NGO score | 0.05 | 0.01 | 0.18 |
|  |  | GINI index | 0.56 | 0.60 | 0.08 |
|  |  | *Socioeconomic adaptive capacity* | *0.63* | *0.52* | *0.03** |
| Social-Ecological | | *Social-ecological vulnerability* | *0.07* | *0.26* | *<0.01*** |
| Covariates | | Percent of coral reefs in MPAs | 0.23 | 0.12 | 0.06 |
|  |  | Fisheries regulation score | 0.7 | 0.66 | 0.62 |
|  |  | WGI score | 0.35 | 0.87 | 0.21 |

We also assessed whether island area and human population size were significant covariates of social-ecological vulnerability to test whether the differences observed between independent islands and territories could be attributed to differences in land area and population, as independent islands have larger areas and populations on average (land area: mean_Independent_ = 16,500 km^2^, mean_Territory_ = 864 km^2^, p = 0.01; population size: mean_Independent_ = 2,900,000, mean_Territory_ = 290,000, p = 0.01). We included island area and population size as potential covariates in the generalized linear models, using the island area data we compiled for estimating reef fish diversity (Table S7) and the 2014 population data we used to estimate the contribution of reef-based tourism to GDP (Table S11). Adding island population size and land area as potential covariates in the model did not change the resulting best-fit model for social-ecological vulnerability, which includes only fisheries regulations and sovereign status as covariates (Table 1). A model that includes fisheries regulations, sovereign status, island area, and population size explains more of the variation (R^2^ = 0.51), but population size and island area are not significant explanatory variables in the model (p > 0.2 for both variables) and the model increases the Aikake Information Criterion relative to the more parsimonious model (with just fisheries regulations and sovereign status as covariates) while failing to perform significantly better.

The best-fit models for ecological sensitivity, ecological recovery potential, and socioeconomic exposure changed when we added island area and population size as potential covariates (Table S27). The best-fit model for ecological sensitivity included both the percent of coral reefs in MPAs and the island’s population size and described more variation than a model without population as a covariate (R^2^ = 0.22, p = 0.04). However, the coefficient for island population was very small (3.96x10^-8^ ), indicating that an island’s population size does not relate strongly to ecological sensitivity. The best-fit model for ecological recovery potential included island area rather than fisheries regulations but again explained only 1% of the variation across islands. The best-fit model for socioeconomic exposure had population size and sovereignty status as covariates, but the model explained less of the variation than the best-fit model without population, which included sovereignty and Worldwide Governance Indicators as covariates. Because island population size and land area did not add significant explanatory value to our models, we excluded them from the models presented in Table 1.

**Table S27.** Components of vulnerability for which the best-fit generalized linear models changed when island area and human population size were included as potential covariates. *β_C_* is the model coefficient for the percent of coral reefs in MPAs, *β_P_* is for the size of the island’s human population, *β_A_* is for the island’s land area (km^2^), and *β_S_* is for island sovereignty; a positive coefficient indicates a positive relationship between independent sovereignty and the associated vulnerability component. The best-fit models for ecological exposure, socioeconomic sensitivity, socioeconomic adaptive capacity, and overall social-ecological vulnerability did not change with the addition of island area and population size as potential covariates.

| Vulnerability component | Model coefficient(s) | Model fit |
| --- | --- | --- |
| Ecological sensitivity | *β_C_ =* -0.01 *  *β_P_ =* 3.96x10^-8^ * | pseudo R^2^ = 0.22 * |
| Ecological recovery potential | *β_A_ =* 1.42x10^-6^ | R^2^ = 0.01* |
| Socioeconomic exposure | *β_P_ =* 4.69x10^-9^ *,  *β_S_ =* 0.03 | pseudo R^2^ = 0.36 ** |

* p ≤ 0.05, ** p ≤ 0.01, *** p ≤ 0.001

The best-fit model for ecological exposure included WGI scores and sovereignty status as covariates (Table 1). To further explore the explanatory value of this model, we ran a separate model selection process for each of the two variables comprising ecological exposure as the dependent variable: the Maina et al. (2011) model values of environmental stress and the watershed-based threat multipliers (Table S1). The Maina et al. (2011) model included environmental factors such as sea surface temperature, tidal ranges, solar radiation, and offshore chlorophyll concentrations, all factors that are unlikely to be explained by island-level governance factors. The best-fit model for predicting these environmental factors had only one covariate: status as an independent island (*β_S_ =* 0.24). This model was only marginally significant (p = 0.07) and explained 12% of the variation in environmental stress scores.

The best-fit model for the watershed-based threat multiplier included independent status, population size, and land area as independent variables and explained 63% of the variation in watershed-based sedimentation, pollution, and nutrient enrichment (p < 0.001). A model with just sovereignty status explained 25% of the variation (p < 0.01), implying that a large part of the explanatory value of the model for ecological exposure derives from the correlation between sovereignty and ecological exposure (independent islands have higher ecological exposure). A model with just population explained 28% of the variation (p < 0.01), implying that the link between sovereignty and ecological exposure may stem from larger populations on independent islands. This in turn could suggest that the link between sovereignty as a covariate and ecological exposure is not particularly instructive.

# References

1. McCarthy J, Canziani O, Leary N, Dokken D, White K. Climate change 2001: impacts, adaptation, and vulnerability. In: Contribution of Working Group II to the Third Assessment Report of the Intergovernmental Panel on Climate Change. Cambridge, UK: Cambridge University Press; 2001.

2. Allison EH, Perry, Allison L, Badjeck M-C, Adger WN, Brown K, Conway D, et al. Vulnerability of national economies to the impacts of climate change on fisheries. Fish Fish [Internet]. 2009;10:173–96.

3. Hughes S, Yau A, Max L, Petrovic N, Davenport F, Marshall M, et al. A framework to assess national level vulnerability from the perspective of food security: The case of coral reef fisheries. Environ Sci Policy [Internet]. 2012;23:95–108.

4. Cinner JE, Huchery C, Darling ES, Humphries AT, Graham NAJ, Hicks CC, et al. Evaluating social and ecological vulnerability of coral reef fisheries to climate change. PLoS One [Internet]. 2013;8(9):e74321–e74321.

5. Maina J, McClanahan TR, Venus V, Ateweberhan M, Madin J. Global gradients of coral exposure to environmental stresses and implications for local management. PLoS One. 2011;6(8).

6. UNEP-WCMC, WorldFish Centre, World Resources Institute, The Nature Conservancy. Global distribution of warm-water coral reefs, compiled from multiple sources including the Millennium Coral Reef Mapping Project, Version 1.3 [Internet]. Cambridge, UK: UNEP World Conservation Monitoring Centre; 2010.

7. MarineRegions.org. World EEZ v8 (2014-02-28) [Internet]. Oostende, Belgium: Vlaams Instituut voor de Zee (VLIZ); 2014. Available from: http://www.marineregions.org/downloads.php

8. ESRI. ArcGIS Desktop. Redlands, CA: Environmental Systems Research Institute; 2011.

9. Philipp E, Fabricius K. Photophysiological stress in scleractinian corals in response to short-term sedimentation. J Exp Mar Bio Ecol. 2003;287(1):57–78.

10. Wooldridge SA, Done TJ. Improved water quality can ameliorate effects of climate change on corals. Ecol Appl. 2009;19(6):1492–9.

11. Vega Thurber RL, Burkepile DE, Fuchs C, Shantz AA, Mcminds R, Zaneveld JR. Chronic nutrient enrichment increases prevalence and severity of coral disease and bleaching. Glob Chang Biol. 2014;20(2):544–54.

12. Burke L, Reytar K, Spalding M, Perry A. Reefs at Risk Revisited. Washington, D.C.; 2011.

13. World Resources Institute. Reefs at Risk Revisited GIS Data Sets: Local Threats. 2011.

14. McClanahan TR, Baird AH, Marshall PA, Toscano MA. Comparing bleaching and mortality responses of hard corals between southern Kenya and the Great Barrier Reef, Australia. Mar Pollut Bull. 2004;48(3–4):327–35.

15. Yee SH, Santavy DL, Barron MG. Comparing environmental influences on coral bleaching across and within species using clustered binomial regression. Ecol Modell. 2008;218(1–2):162–74.

16. Pandolfi JM, Connolly SR, Marshall DJ, Cohen AL. Projecting Coral Reef Futures Under Global Warming and Ocean Acidification. Science (80- ) [Internet]. 2011;333(6041):418–22.

17. Fournie JW, Vivian DN, Yee SH, Courtney LA, Barron MG. Comparative sensitivity of six scleractinian corals to temperature and solar radiation. Dis Aquat Organ. 2012;99(2):85–93.

18. Swain TD, Vega-Perkins JB, Oestreich WK, Triebold C, DuBois E, Henss J, et al. Coral bleaching response index: a new tool to standardize and compare susceptibility to thermal bleaching. Glob Chang Biol. 2016;22(7):2475–88.

19. Darling ES, Alvarez-Filip L, Oliver TA, Mcclanahan TR, Côté IM. Evaluating life-history strategies of reef corals from species traits. Ecol Lett. 2012;15(12):1378–86.

20. Rowan R. Coral bleaching: Thermal adaptation in reef coral symbionts. Nature [Internet]. 2004;430(7001):742–742.

21. Eakin CM, Morgan JA, Heron SF, Smith TB, Liu G, Alvarez-Filip L, et al. Caribbean corals in crisis: Record thermal stress, bleaching, and mortality in 2005. PLoS One. 2010;5(11).

22. Bouchon C, Portillo P, Bouchon-Navaro Y, Max L, Hoetjes P, Brathwaite A, et al. Status of coral reefs of the Lesser Antilles after the 2005 coral bleaching event. In: Wilkinson C, Souter D, editors. Status of Caribbean coral reefs after bleaching and hurricanes in 2005. Townsville: Global Coral Reef Monitoring Network and Reef and Rainforest Research Centre; 2008. p. 85–103.

23. McKenna SA, Etnoyer P. Rapid assessment of stony coral richness and condition on Saba Bank, Netherlands Antilles. PLoS One. 2010;5(5).

24. FORCE. Summary of Dominican Republic ecological surveys [Internet]. 2011. Available from: http://reefcheck.org/PDFs/FORCE_DR.pdf

25. IUCN. The IUCN Red List of Threatened Species, Version 2017-1 [Internet]. 2017. Available from: www.iucnredlist.org

26. Fukami H, Budd AF, Levitan DR, Jara J, Kersanach R, Knowlton N. Geographic differences in species boundaries among members of the Montastraea annularis complex based on molecular and morphological markers. Evolution (N Y). 2004;58(2):324–37.

27. Spalding MD, Fox HE, Allen GR, Davidson N, Ferdaña ZA, Finlayson M, et al. Marine Ecoregions of the World: A Bioregionalization of Coastal and Shelf Areas. Bioscience [Internet]. 2007;57(7):573.

28. Deleveaux VKW, Kramer P, Kramer PR, Schill S. Southeastern Bahamas Coral Reef & Island Survey: Rapid Ecological Assessment Report. 2013.

29. Dikou A, Ackerman C, Banks C, Dempsey A, Fox M, Gins M, et al. Ecological assessment to detect imminent change in coral reefs of Admiral Cockburn Land and Sea National Park, Turks and Caicos Islands. Mar Ecol. 2009;30(4):425–36.

30. Gumbs K. An examination of changes or trends in key health indicators on critical habitats around Anguilla, BWI. Univeristy of the West Indies; 2012.

31. Ruttenberg BI, Johnson AE, Caselle J, Estep A, Grenda D, Marhaver K, et al. Ecological Assessment of Barbuda’s Marine Ecosystems: Science supporting the Barbuda Blue Halo Initiative. 2013.

32. Oxenford HA, Roach R, Brathwaite A, Nurse L, Goodridge R, Hinds F, et al. Quantitative observations of a major coral bleaching event in Barbados, Southeastern Caribbean. Clim Change. 2008;87(3–4):435–49.

33. Steiner SCC, Kerr JM. Stony corals in Dominica during the 2005 bleaching episode and one year later. Rev Biol Trop. 2008;56(May):139–48.

34. Cowan C. Coral Bleaching and Disease : Recovery and Mortality on Martinique Reefs following the 2005 Caribbean Bleaching Event. University of Newcastle upon Tyne; 2006.

35. Wild R, Slade L, Pardee M, Carleton C. Towards multi-user marine management in Montserrat. 2007.

36. Debrot AO, Houtepen E, Meester EH, van Beek I, Timmer T, Boman E, et al. Habitat diversity and bio- diversity of the benthic seascapes of St. Eustatius Adolphe. The Hague, Netherlands; 2014.

37. Bruckner A, Williams A. Assessment of the Community Structure, Status, Health and Resilience of Coral Reefs off St. Kitts and Nevis. Landover, MD; 2012.

38. Rothenberger P, Blondeau J, Cox C, Curtis S, Fisher WS, Garrison V, et al. The state of coral reef ecosystems of the US Virgin Islands [Internet]. The State of Coral Reef Ecosystems of the United States and Pacific Freely Associated States: 2008. 2008.

39. Jones L, Alcolado PM, Cala Y, Cobian D, Coelho V, Hernandez A, et al. The effects of coral bleaching in the Northern Caribbean and Western Atlantic. In: Wilkinson C, Souter D, editors. Status of the Caribeean coral reefs after bleaching and hurricanes in 2005. Townsville, Australia: Global Coral Reef Monitoring Network; Reef and Rainforest Research Centre; 2008. p. 73–83.

40. Alcolado PM. Resiliencia en crestas de arrecifes coralinos del este del golfo de Batabanó, Cuba, y factores determinantes probables. Ser Ocean. 2013;13:49–75.

41. Perera-Valderrama S, Hernendez-Arana H, Ruiz-Zarate MA, Alcolado PM, Caballero-Aragon H, Gonzalez-Cano J, et al. Condition assessment of coral reefs of two marine protected areas under different regimes of use in the north-western Caribbean. Ocean Coast Manag. 2016;127:16–25.

42. Bruckner AW, Beck B, Renaud P. The status of coral reefs and associated fishes and invertebrates of commercial importance in Pedro Bank, Jamaica. Rev Biol Trop [Internet]. 2014;62(September):11.

43. NCCOS. National Coral Reef Monitoring Program: Assessment of coral reef benthic communities in Puerto Rico from 2014-05-19 to 2014-12-03 (NCEI Accession 0151729). NOAA National Centers for Environmental Information; 2016.

44. Steneck RS, Arnold SN, León R De, Rasher DB. Status and Trends of Bonaire’s Coral Reefs in 2015: Slow but steady signs of resilience. University of Maine, School of Marine Science. 2015.

45. Mallela J, Parkinson R, Day O. An Assessment of Coral Reefs in Tobago. Caribb J Sci. 2010;46(1):83–7.

46. Wilson SK, Graham NAJ, Pratchett MS, Jones GP, Polunin NVC. Multiple disturbances and the global degradation of coral reefs: Are reef fishes at risk or resilient? Glob Chang Biol. 2006;12(11):2220–34.

47. Graham NAJ, Chabanet P, Evans RD, Jennings S, Letourneur Y, Aaron Macneil M, et al. Extinction vulnerability of coral reef fishes. Ecol Lett. 2011;14:341–8.

48. Pratchett MS, Munday PL, Wilson SK, Graham NAJ, Cinner JE, Bellwood DR, et al. Effects of climate-induced coral bleaching on coral-reef fishes — ecological and economic consequences. 2008;251–96.

49. Pauly D, Zeller D. Sea Around Us Data [Internet]. 2015. Available from: seaaroundus.org

50. Carpenter KE. The Living Marine Resources of the Western Central Atlantic. Rome, Italy: FAO Species Identification Guide for Fishery Purposes; 2002.

51. Green AL, Maypa AP, Almany GR, Rhodes KL, Weeks R, Abesamis RA, et al. Larval dispersal and movement patterns of coral reef fishes, and implications for marine reserve network design. Biol Rev. 2015;90(4):1215–47.

52. Kramer DL, Chapman MR. Implications of fish home range size and relocation for marine reserve function. Environ Biol Fishes [Internet]. 1999;55(1):65–79.

53. Froese R, Pauly D. FishBase [Internet]. 2017. Available from: www.fishbase.org

54. Palomares MLD, Pauly D. SeaLifeBase [Internet]. 2017. Available from: www.sealifebase.org

55. Courtenay WR, Sahlman HF. Pomadasyidae. In: Fischer W, editor. FAO Species Indentification Sheets for Fishery Purposes: Western Central Atlantic (Fishing Area 31), vol 4. Rome; 1978.

56. Cervigón F, Cipriani R, Fischer W, Garibaldi L, Hendrickx M, Lemus AJ, et al. Guía de campo de las especies comerciales marinas y de aquas salobres de la costa septentrional de Sur América. Rome; 1992.

57. Billings VC, Munro JL. The biology, ecology, exploitation and management of Caribbean reef fishes: Pomadasydae (grunts). Res Reports Zool Dep Univ West Indies. 1974;3(5):1–128.

58. Manooch, C.S. I, Barans CA. Distribution, abundance, age and growth of the tomtate, Haemulon aurolineatum, along the south-eastern United States coast. Fish Bull. 1982;80(1):1–20.

59. Gomon MF. Labridae. In: Fischer W, editor. FAO Species Indentification Sheets for Fishery Purposes: Western Central Atlantic (Fishing Area 31), vol 4. Rome: FAO; 1978.

60. Schneider W. Field guide to the commercial marine resources of the Gulf of Guinea. In: FAO Species Identification Sheets for Fishery Purposes. Rome: FAO; 1990. p. 268.

61. Allen G. Snappers of the world: an annotated and illustrated catalogue of lutjanid species known to date. FAO Fish Synopsis. 1985;125(6):208.

62. Murray PA, Chinnery LE, Moore EA. The recruitment of the queen snapper, Etelis oculatus Val., into the St. Lucian fishery: recruitment of fish and recruitment of fishermen. In: Proceedings of the 41st Gulf and Caribbean Fisheries Institute. 1992. p. 297–303.

63. Smith CL. National Audubon Society field guide to tropical marine fishes of the Caribbean, the Gulf of Mexico, Florida, the Bahamas, and Bermuda. New York: Alfred A. Knopf, Inc.; 1997. 720 p.

64. Cervigón F. Los peces marinos de Venezuela, vol. 2. Caracas: Fundación Científica Los Roques; 1993. 497 p.

65. Murray PA, Moore EA. Some morphometric relationships in Etelis oculatus Valenciennes (Queen snapper), landed in St. Lucia, W.I. In: Proceedings of the 41st Gulf and Caribbean Fisheries Institute. 1992. p. 416–21.

66. Grimes CB. Age, growth and length-weight relationships of vermilion snapper, Rhomboplites aurorubens from North Carolina and South Carolina waters. Trans Am Fish Soc. 1978;107(3):454–6.

67. Claro R, García-Arteaga JP. Crecimiento. In: Claro R, editor. Ecología de los peces marinos de Cuba. Instituto de Oceanologia Academia de Ciencias de Cuba and Centro de Investigaciones de Quintana Roo; 1994. p. 321–402.

68. Burton ML. Age, growth and mortality of mutton snapper, Lutjanus analis, from the east coast of Florida, with a brief discussion of management implications. Fish Res. 2002;59(1–2):31–41.

69. Thompson R, Munro JL. The biology, ecology and bionomics of Caribbean reef fishes: Lutjanidae (snappers). In: Munro JL, editor. Caribbean coral reef fishery resources. ICLARM Studies Rev. 7; 1983. p. 94–109.

70. Manooch, C.S. I, Matheson RH. Age, growth and mortality of gray snapper collected from Florida waters. In: Proceedings of the Annual Conference of the Southeastern Association of Fisheries and Wildlife Agencies. 1983. p. 331–44.

71. Claro R, García-Cagide A, García-Arteaga JP, Sierra LM. Peculiaridades biológicos de Lutjanus jocu (Pisces: Lutjanidae) en las zonas nororiental y suroccidental de la plataforma cubana. Ecol Trop.

72. Claro R. Nutrición. Ecología y ciclo de vida de la biajaiba, Lutjanus synagris (Linnaeus), en la plataforma cubana. 1981.

73. Manooch, C.S. I, Mason DL. Age, growth and mortality of lane snapper from Southern Florida. Northeast Gulf Sci. 1984;7:109–15.

74. Johnson AG. Age and growth of yellowtail snapper from South Florida. Trans Am Fish Soc. 1983;112(2a):173–7.

75. Bertelsen RD, Hornbeck J. Using acoustic tagging to determine adult spiny lobster *(Panulirus argus)* movement patterns in the Western Sambo Ecological Reserve (Florida, United States). New Zeal J Mar Freshw Res [Internet]. 2009;43(1):35–46.

76. Lozano-Álvarez E, Carrasco-Zanini G, Briones-Fourzán P. Homing and orientation in the spotted spiny lobster, Panulirus guttatus (Decapoda, Palinuridae), towards a subtidal coral reef habitat. Crustaceana. 2002;75(7):859–73.

77. Heemstra PC, Randall JE. Groupers of the world (family Serranidae, subfamily Epinephelinae): an annotated and illustrated catalogue of the grouper, rockcod, hind, coral grouper and lyretail species known to date. In: FAO Fisheries Synopsis. Rome: FAO; 1993. p. 382.

78. Smith CL. Serranidae. In: Fischer W, editor. FAO Species Indentification Sheets for Fishery Purposes: Western Central Atlantic (Fishing Area 31), vol 4. Rome: FAO; 1978.

79. Courtenay WR. Grammistidae. In: Fischer W, Bianchi G, Scott WB, editors. FAO Species Indentification Sheets for Fishery Purposes: Eastern Central Atlantic (Fishing Area 34, 47 (in part)), vol 2. Rome: FAO; 1981.

80. Johnson AG, Collins LA. Age-size structure of red grouper, (Epinephelus morio), from the eastern Gulf of Mexico. Northeast Gulf Sci. 1994;13(2):101–6.

81. Vergara R. Sphyraenidae. In: Fischer W, editor. FAO Species Indentification Sheets for Fishery Purposes: Western Central Atlantic (Fishing Area 31), vol 4. Rome: FAO; 1978.

82. De Sylva DP. Sphyraenidae. In: Fischer W, Bianchi G, Scott WB, editors. FAO Species Indentification Sheets for Fishery Purposes: Eastern Central Atlantic (Fishing Area 34, 47 (in part)), vol 2. Rome: FAO; 1981.

83. Jackson JBC, Donovan MK, Cramer KL, Lam V, Lam W. Status and Trends of Caribbean Coral Reefs: 1970-2012. Global Coral Reef Monitoring Network. Gland, Switzerland; 2014.

84. McClanahan TR, Donner SD, Maynard JA, MacNeil MA, Graham NAJ, Maina J, et al. Prioritizing Key Resilience Indicators to Support Coral Reef Management in a Changing Climate. PLoS One. 2012;7(8):e42884.

85. Zhang SY, Speare KE, Long ZT, McKeever KA, Gyoerkoe M, Ramus AP, et al. Is coral richness related to community resistance to and recovery from disturbance? PeerJ [Internet]. 2014;2:e308.

86. Côté IM, Darling ES. Rethinking ecosystem resilience in the face of climate change. PLoS Biol. 2010;8(7).

87. Mumby PJ, Harborne AR, Raines PS, Ridley JM. A critical assessment of data derived from Coral Cay Conservation volunteers. Bull Mar Sci. 1995;56(3):737–51.

88. Harding S, Lowery C, Oakley S. Comparison between complex and simple reef survey techniques using volunteers: Is the effort justified? In: Proceedings of the 9th International Coral Reef Symposium. 2000. p. 883–9.

89. Holt BG, Rioja-Nieto R, Aaron Macneil M, Lupton J, Rahbek C. Comparing diversity data collected using a protocol designed for volunteers with results from a professional alternative. Methods Ecol Evol. 2013;4(4):383–92.

90. Bruno JF, Sweatman H, Precht WF, Selig ER, Schutte VGW. Assessing evidence of phase shifts from coral to macroalgal dominance on coral reefs. Ecology. 2009;90(6):1478–84.

91. Karr KA, Fujita R, Halpern BS, Kappel C V., Crowder L, Selkoe KA, et al. Thresholds in Caribbean coral reefs: Implications for ecosystem-based fishery management. J Appl Ecol. 2015;52(2):402–12.

92. Edgar GJ, Stuart-Smith RD, Willis TJ, Kininmonth S, Baker SC, Banks S, et al. Global conservation outcomes depend on marine protected areas with five key features. Nature [Internet]. 2014;506(7487):216–20.

93. Mumby PJ, Hastings A, Edwards HJ. Thresholds and the resilience of Caribbean coral reefs. Nature [Internet]. 2007;450(7166):98–101.

94. Roff G, Mumby PJ. Global disparity in the resilience of coral reefs. Trends Ecol Evol. 2012;27(7):404–13.

95. McCook LJ, Jompa J, Diaz-Pulido G. Competition between corals and algae on coral reefs: A review of evidence and mechanisms. Coral Reefs. 2001;19(4):400–17.

96. Hughes TP. Community structure and diversity of coral reefs: the role of history. Ecology. 1989;70(December 1985):275–9.

97. Aronson RB, Precht WF. Herbivory and algal dynamics on the coral reef at Discovery Bay, Jamaica. Limnol Ocean. 2000;45(1):251–5.

98. Aronson RB, Precht WF. Conservation, precaution, and Caribbean reefs. Coral Reefs. 2006;25(3):441–50.

99. Check R. Global Reef Tracker [Internet]. 2016. Available from: http://data.reefcheck.us/

100. Rohatgi A. WebPlotDigitizer [Internet]. 2017. Available from: http://arohatgi.info/WebPlotDigitizer/

101. Bozec Y-M, O’Farrell S, Bruggemann JH, Luckhurst BE, Mumby PJ. Tradeoffs between fisheries harvest and the resilience of coral reefs. Proc Natl Acad Sci [Internet]. 2016;113(16):201601529.

102. Hughes TP, Graham NAJ, Jackson JBC, Mumby PJ, Steneck RS. Rising to the challenge of sustaining coral reef resilience. Trends Ecol Evol. 2010;25(11):633–42.

103. Byce S. The relationship between live coral and macroalgae in South Caicos as influenced by herbivorous fishes. 2012;

104. Wynne S. Status of Anguilla’s Marine Resources 2010. 2010.

105. Rémi G, Malterre P, Franck M, Roncuzzi F, Quod J-P. Reseau Reef Check Caraibe- Année 2011: suivi Guadeloupe, Saint- Martin et Martinique. 2012.

106. Waitt Institute. Marine scientific assessment: the state of Montserrat’s marine resources. 2016.

107. Toller W, Debrot AO, Vermeij MJA, Hoetjes PC. Reef fishes of Saba Bank, Netherlands Antilles: Assemblage structure across a gradient of habitat types. PLoS One. 2010;5(5):e9207.

108. Noble MM, van Laake G, Berumen ML, Fulton CJ. Community Change within a Caribbean Coral Reef Marine Protected Area following Two Decades of Local Management. PLoS One. 2013;8(1):25–7.

109. Meesters EHWG. Saba Bank; health status 2010. Wageningen; 2010.

110. PARETO. Sante Des Reserves Naturelles Marines de Guadeloupe, Saint-Martin Et Saint-Barthelemy: Etat des lieux 2012 et évolution 2007-2012. 2012.

111. de Graaf M, Piontek S, Miller DCM, Brunel T, Nagelkerke LAJ. Status and trends of St. Eustatius coral reef ecosystem and fisheries: 2015 report card [Internet]. IMARES report;C167/15. The Hague, Netherlands; 2015.

112. Williams SM, Sánchez-Godínez C, Newman SP, Cortés J. Ecological assessments of the coral reef communities in the Eastern Caribbean and the effects of herbivory in influencing coral juvenile density and algal cover. Mar Ecol. 2017;38(2).

113. Perry CT, Steneck RS, Murphy GN, Kench PS, Edinger EN, Smithers SG, et al. Regional-scale dominance of non-framework building corals on Caribbean reefs affects carbonate production and future reef growth. Glob Chang Biol. 2015;21(3):1153–64.

114. Nugraha WA. A macroecological study of Caribbean parrotfishes [Internet]. Newcastle University; 2016.

115. Chatenoux B, Wolf A. Ecosystem based approaches for climate Caribbean SIDS. Geneva; 2013.

116. Steneck RS. Patterns and trends in abundance of corals and seaweeds at monitored sites in Fish Protection Areas and control sites. In: Steneck RS, Arnold SN, de León R, Rasher DB, editors. Status and Trends of Bonaire’s Coral Reefs in 2015: Slow but Steady Signs of Resilience. 2015. p. 14–22.

117. Arnold SN. Status and trends of Bonaire’s herbivorous fishes. In: Steneck RS, Arnold SN, de León R, Rasher DB, editors. Status and Trends of Bonaire’s Coral Reefs in 2015: Slow but Steady Signs of Resilience. 2015. p. 23–31.

118. Boyle K. The status and trends of sea urchins Diadema antillarum and Echinometra viridis on the leeward coral reefs of Bonaire. In: Steneck RS, Arnold SN, de Leon R, Rasher DB, editors. Status and Trends of Bonaire’s Coral Reefs in 2015: Slow but Steady Signs of Resilience. 2015. p. 32–41.

119. Cinner JE, Huchery C, Darling ES, Humphries AT, Graham NAJ, Hicks CC, et al. Evaluating social and ecological vulnerability of coral reef fisheries to climate change. PLoS One [Internet]. 2013;8(9):e74321–e74321.

120. Miloslavich P, Díaz JM, Klein E, Alvarado JJ, Díaz C, Gobin J, et al. Marine biodiversity in the Caribbean: Regional estimates and distribution patterns. PLoS One. 2010;5(8):e11916.

121. Churchyard T, Eaton M, Hall J, Millett J, Farr A, Cuthbert R, et al. The UK’s wildlife overseas: a stocktake of nature in our Overseas Territories. Sandy, UK; 2014.

122. Steiner SCC. Coral Reefs of Dominica (Lesser Antilles). Ann Naturhist Mus Wien, B. 2015;117:47–119.

123. Churchyard T, Eaton MA, Havery S, Hall J, Millett J, Farr A, et al. The biodiversity of the United Kingdom’s Overseas Territories: a stock take of species occurrence and assessment of key knowledge gaps. Biodivers Conserv. 2016;25(9):1677–94.

124. Marechal JP, Peres C. Suivi de l’etat de sante des recifs coralliens de la Martinique. Fort de France; 2006.

125. Rusk BL. Conserving Biodiversity and reducing habitat degradation in Protected Areas and their Areas of Influence. St. Kitts & Nevis; 2014.

126. Diaz N, Cuzange P-A. Plan De Gestion De La Reserve Naturelle Nationale De L’Ile De Saint-Martin Et Des Sites Du Conservatoire De L’Espace Littoral Et Des Rivages Lacustres. Guadeloupe, FWI; 2009.

127. Rogers CS, Miller J, Muller EM, Edmunds P, Nemeth RS, Beets JP, et al. Ecology of Coral Reefs in the US Virgin Islands. In: Riegl B, Dodge RE, editors. Coral Reefs of the USA [Internet]. Dordrecht: Springer Science+Business; 2008. p. 303–373.

128. Mouillot D, Villeger S, Parravicini V, Kulbicki M, Arias-Gonzalez JE, Bender M, et al. Functional over-redundancy and high functional vulnerability in global fish faunas on tropical reefs. Proc Natl Acad Sci [Internet]. 2014;111(38):13757–62.

129. Nyström M, Graham NAJ, Lokrantz J, Norström A V. Capturing the cornerstones of coral reef resilience: Linking theory to practice. Vol. 27, Coral Reefs. 2008. p. 795–809.

130. Duffy JE, Lefcheck JS, Stuart-Smith RD, Navarrete SA, Edgar GJ. Biodiversity enhances reef fish biomass and resistance to climate change. Proc Natl Acad Sci [Internet]. 2016;113(22):6230–5.

131. Burkepile DE, Hay ME. Impact of herbivore identity on algal succession and coral growth on a Caribbean reef. PLoS One. 2010;5(1):e8963.

132. Sandin SA, Vermeij MJA, Hurlbert AH. Island biogeography of Caribbean coral reef fish. Glob Ecol Biogeogr. 2008;17(6):770–7.

133. CIA. The World Factbook [Internet]. 2017 [cited 2017 Jan 1]. Available from: https://www.cia.gov/library/publications/the-world-factbook/index.html

134. de Bettencourt J, Imminga-Berends H. Overseas Countries and Territories: Environmental Profiles, Final Report, Part 2. Brussels, Belgium; 2015.

135. PAHO. French Guiana, Guadeloupe, and Martinique [Internet]. Pan American Health Organization. 2013.

136. Béné C, Tewfik A. Fishing effort allocation and fishermen’s decision making process in a multi-specific small-scale fishery: analysis of the Conch and Lobster fishery in Turks and Caicos Islands. Hum Ecol. 2001;29(2):157–86.

137. Hawkins JP, Roberts CM. Effects of artisanal fishing on Caribbean coral reefs. Conserv Biol. 2004;18(1):215–26.

138. Teh LSL, Teh LCL, Sumaila UR. A Global Estimate of the Number of Coral Reef Fishers. PLoS One. 2013;8(6).

139. Boekhoudt BG. Aruba. In: Singh-Renton S, McIvor I, editors. Review of current fisheries management performance and conservation measures in the WECAFC area. Rome: FAO Fisheries and Aquaculture Techincal Paper No. 587; 2015. p. 79–88.

140. Gumbs J, Rawlins K. Anguilla National Report. In: Report of the Third Annual CRFM Scientific Meeting. Kingstown, St. Vincent & the Grenadines: CRFM; 2007. p. 4.

141. UNDESA. World Population Prospects: The 2015 Revision [Internet]. Population Division of the UN Department of Economic and Social Affairs; 2015.

142. Horsford I. Antigua and Barbuda. In: Singh-Renton S, McIvor I, editors. Review of current fisheries management performance and conservation measures in the WECAFC area. Rome: FAO Fisheries and Aquaculture Techincal Paper No. 587; 2015. p. 65–78.

143. World Bank. Population, total [Internet]. World Bank; 2017. Available from: https://data.worldbank.org/indicator/SP.POP.TOTL

144. CRFM. The Bahamas [Internet]. 2013 [cited 2016 Jan 14]. Available from: http://www.crfm.net/index.php?option=com_k2&view=item&id=45:the-bahamas&Itemid=288

145. McConney P. Coastal fisheries of Barbados. In: Salas S, Chuenpagdee R, Charles A, Seijo JC, editors. Coastal fisheries of Latin America and the Caribbean2 [Internet]. Rome, Italy; 2011. p. 49–71. Available from: FAO Fisheries and Agriculture Technical Paper

146. Johnson AE, Jackson JBC. Fisher and diver perceptions of coral reef degradation and implications for sustainable management. Glob Ecol Conserv [Internet]. 2015;3:890–9.

147. PAHO. Netherlands Antilles. Health in the Americas, 2012. Washington, D.C.: Pan American Health Organization; 2012. (Scientific & Technical Publication). Report No.: 636.

148. Ramdeen R, Harper S, Zylich K, Zeller D. Reconstruction of Total Marine Fisheries Catches for the British Virgin Islands (1950-2010). Fish catch Reconstr Islands, Part IV. 2014;22(2):9–16.

149. CARICOM. Demographic profile: British Virgin Islands [Internet]. 2015. Available from: http://caricomstats.org/Files/Databases/Demography/VG.pdf

150. Meier RE, McCoy C, Richardson L, Turner JR. Darwin Initiative to Enhance an Established Marine Protected Area System , Cayman Islands: Quantifying the Impact of Recreational and Artisanal Fisheries in the Cayman Islands. 2011;(April):104.

151. FAO. Perfíles de pesca y acuicultura por países: la república de Cuba. Rome; 2015.

152. Johnson AE. Fish, fishing, diving, and the management of coral reefs. Scripps Institution of Oceanography; 2011.

153. FAO. Fishery and Aquaculture Country Profiles: the Commonwealth of Dominica [Internet]. 2016 [cited 2017 Jul 31]. Available from: http://www.fao.org/fishery/facp/DMA/en

154. FAOSTAT. Annual population, Dominica [Internet]. Rome, Italy; 2017. Available from: http://www.fao.org/faostat/en/#data/OA

155. Mateo J. Dominican Republic. In: Singh-Renton S, McIvor I, editors. Review of current fisheries management performance and conservation measures in the WECAFC area. Rome: FAO Fisheries and Aquaculture Techincal Paper No. 587; 2015. p. 137–60.

156. Baldeo R. Coastal fisheries of Grenada. In: Salas S, Chuenpagdee R, Charles A, Seijo JC, editors. Coastal fisheries of Latin America and the Caribbean. Rome: FAO Fisheries and Aquaculture Technical Paper 544; 2011. p. 219–30.

157. Cotis J-P. Recensement de la population: Populations légales en vigueur à compter du 1er janvier 2011. Paris; 2011.

158. Ramdeen R, Belhabib D, Harper S, Zeller D. Reconstruction of Total Marine Fisheries Catches for Haiti and Navassa Island (1950–2010). 2012;20:37–45.

159. MARNDR. Programme national pour le développement de la pêche maritime en Haïti 2010-2014. Haiti; 2010.

160. Carr LM, Heyman WD. Jamaica bound? Marine resources and management at a crossroads in Antigua and Barbuda. Geogr J. 2009;175(1):17–38.

161. Murray A. National Report on the Spiny Lobster Fishery in Jamaica. In: CRFM, editor. Report of the Third Annual Scientific Meeting - Kingstown, St Vincent & the Grenadines, 17-26 July 2007 - National Reports. Kingstown, St. Vincent & the Grenadines; 2007. p. 31–42.

162. Ponteen A. Montserrat National Fisheries Report. Montserrat; 2014.

163. Valle-Esquivel M, Shivlani M, Matos-Caraballo D, Die DJ. Coastal fisheries of Puerto Rico. In: Salas S, Chuenpagdee R, Charles A, Seijo JC, editors. Coastal fisheries of Latin America and the Caribbean. Rome: FAO Fisheries and Aquaculture Techincal Paper No. 544; 2011. p. 285–314.

164. Central Bureau of Statistics. Statistical Yearbook of the Netherlands Antilles, 2009. Willemstad, Curacao; 2009.

165. Jadot C. Protection de l’environnement à Saint-Barthélemy - Connaissances actuelles et recommandations en matière de recherche. Bronx, NY; 2016.

166. INSEE. Nationalité et immigration en 2014: Recensement de la population [Internet]. 2017. Available from: https://www.insee.fr/fr/statistiques/2867636?sommaire=2867837

167. Dilrosun F. Inventory of the fishery sector of St. Eustatius. 2004.

168. Dilrosun F. Inventory of the fishery sector of St. Maarten. 2004.

169. Masters J. CRFM Statistics and Information Report for 2010. Belize and St. Vincent and the Grenadines; 2010.

170. Shing CCA, Maharaj S. Trinidad and Tobago. In: Singh-Renton S, McIvor I, editors. Review of current fisheries management performance and conservation measures in the WECAFC area. Rome: FAO Fisheries and Aquaculture Techincal Paper No. 587; 2015. p. 259–74.

171. Kojis BL, Quinn NJ. A census of US Virgin Islands commercial fishers at the start of the 21st century. In: Proceedings of 10th International Coral Reef Symposium. 2006. p. 1326–34.

172. FAO. Fishery and Aquaculture Statistics: Global fisheries commodities production and trade 1976-2013 [Internet]. Rome: FAO Fisheries and Aqauculture Department; 2016 [cited 2017 Aug 1]. Available from: www.fao.org/fishery/statistics/software/fishstatj/en

173. FAO. Fishery and Aquaculture Statistics: Global capture production 1950-2015 (FishstatJ) [Internet]. Rome: FAO Fisheries and Aqauculture Department; 2017 [cited 2017 Aug 1]. Available from: www.fao.org/fishery/statistics/software/fishstatj/en

174. Spalding M, Burke L, Wood SA, Ashpole J, Hutchison J, zu Ermgassen P. Mapping the global value and distribution of coral reef tourism. Mar Policy [Internet]. 2017;82(January):104–13.

175. World Bank. GDP (constant 2010 US$) [Internet]. 2017 [cited 2017 May 7]. Available from: http://data.worldbank.org/indicator/NY.GDP.MKTP.KD

176. World Bank. Consumer price index (2010=100) [Internet]. 2017 [cited 2017 Jun 7]. Available from: http://data.worldbank.org/indicator/FP.CPI.TOTL?locations=US

177. IEDOM. Outlook for Saint-Barthélemy. Express Note [Internet]. 2011;134. Available from: www.iedom.fr

178. IEDOM. Saint-Martin at a Glance. Express Note. 2013;(227).

179. Central Bureau of Statistics. Tourism in the Caribbean Netherlands in 2014 [Internet]. 2015 [cited 2017 Jul 10]. Available from: https://www.cbs.nl/en-gb/background/2015/14/tourism-in-the-caribbean-netherlands-in-2014

180. CTO. Latest Statistics 2014 [Internet]. 2015. Available from: http://www.onecaribbean.org/statistics/2003-july-2015-tourism-stats/

181. IEDOM. Le tourisme à la Guadeloupe: Vers un redémarrage durable du secteur? Note Expresse. 2015;305.

182. UNdata. Anguilla [Internet]. UNdata Country Profiles. 2017 [cited 2017 Oct 7]. Available from: http://data.un.org/CountryProfile.aspx?crName=Anguilla

183. UNdata. British Virgin Islands [Internet]. UNdata Country Profiles. 2017 [cited 2017 Oct 7]. Available from: http://data.un.org/CountryProfile.aspx?crName=British Virgin Islands

184. UNdata. Guadeloupe [Internet]. UNdata Country Profiles. 2017 [cited 2017 Oct 7]. Available from: http://data.un.org/CountryProfile.aspx?crName=Guadeloupe

185. CIA. Anguilla [Internet]. CIA World Factbook. 2017 [cited 2017 Oct 7]. Available from: https://www.cia.gov/library/publications/the-world-factbook/geos/av.html

186. Anguilla Government Statistics Department. Anguilla’s Consumer Price Index, 1st Quarter: January-March 2014. The Valley, Anguilla; 2014.

187. UNdata. Aruba [Internet]. UNdata Country Profiles. 2017 [cited 2017 Oct 7]. Available from: http://data.un.org/CountryProfile.aspx?crName=Aruba%0A

188. Organization for Economic Co-operation and Development. Consumer Price Index: Total All Items for the United States (CPALTT01USM657N) - FRED - St. Louis Fed [Internet]. St. Louis: FRED, Federal Reserve Bank of St. Louis; 2018. Available from: https://fred.stlouisfed.org/series/CPALTT01USA661S

189. CBS. Caribbean Netherlands; population (1 January) sex, age [Internet]. The Hague, Netherlands: Centraal Bureau voor de Statistiek; 2017. Available from: http://statline.cbs.nl/Statweb/publication/?VW=T&DM=SLEN&PA=80534ENG&D1=0&D2=0&D3=0&D4=a&D5=8-14&HD=161103-1030&HDR=T,G3&STB=G1,G2,G4

190. Curacao Bureau of Statistics. Caribbean Netherlands: gross domestic product (GDP): Bonaire 2013 [Internet]. StatLine. 2018 [cited 2018 Jan 18]. Available from: https://opendata.cbs.nl/statline/#/CBS/en/dataset/83776ENG/table?dl=399C

191. CIA. British Virgin Islands [Internet]. CIA World Factbook. 2017 [cited 2017 Oct 7]. Available from: https://www.cia.gov/library/publications/the-world-factbook/geos/vi.html%0A

192. ESO. The Cayman Islands’ Annual Economic Report 2013. 2014.

193. Centrale Bank van Curacao en Sint Maarten. Exchange rates [Internet]. [cited 2018 Jan 18]. Available from: http://www.centralbank.cw/exchange-rates

194. Couillaud A. Recensement de la population en Guadeloupe. 2017.

195. IEDOM. Guadeloupe at a glance. Express Note [Internet]. 2014;275(July):4. Available from: http://bjo.bmj.com/cgi/doi/10.1136/bjo.2010.193169

196. INSEE. Consumer price index - Base 2015 - All households - Guadeloupe - All items (Identifier 001769691) [Internet]. 2017 [cited 2018 Jan 19]. Available from: https://www.insee.fr/en/statistiques/serie/001769691#Documentation

197. IRS. Translating foreign currency into U.S. dollars [Internet]. 2017 [cited 2018 Jan 18]. Available from: https://www.irs.gov/individuals/international-taxpayers/yearly-average-currency-exchange-rates

198. Couillaud A. Recensement de la population en Martinique. Fort-De-France, Martinique; 2017.

199. IEDOM. Martinique at a glance. Express Note [Internet]. 2014;273(July):4. Available from: http://bjo.bmj.com/cgi/doi/10.1136/bjo.2010.193169

200. INSEE. Consumer price index - Base 2015 - All households - Martinique - All items (Identifier 001769715) [Internet]. 2017 [cited 2018 Jan 19]. Available from: https://www.insee.fr/en/statistiques/serie/001769715

201. UNdata. Turks & Caicos Islands [Internet]. UNdata Country Profiles. 2017 [cited 2017 Oct 7]. Available from: http://data.un.org/CountryProfile.aspx?crName=Turks and Caicos Islands%0A

202. Cinner J, Fuentes MMPB, Randriamahazo H. Exploring Social Resilience in Madagascar’s Marine Protected Areas. Ecol Soc [Internet]. 2009;14(1):41.

203. Cinner JE, Adger WN, Allison EH, Barnes ML, Brown K, Cohen PJ, et al. Building adaptive capacity to climate change in tropical coastal communities. Nat Clim Chang [Internet]. 2018;8(February):117–23.

204. Allison EH, Ellis F. The livelihoods approach and management of small-scale fisheries. Mar Policy. 2001;25(5):377–88.

205. Brooks N, Adger WN, Kelly PM. The determinants of vulnerability and adaptive capacity at the national level and the implications for adaptation. Glob Environ Chang. 2005;15(2):151–63.

206. Thiault L, Marshall P, Gelcich S, Collin A, Chlous F, Claudet J. Mapping social–ecological vulnerability to inform local decision making. Conserv Biol. 2018;32(2):447–56.

207. Ibrahim SS, Ozdeser H, Cavusoglu B. Vulnerability to recurrent shocks and disparities in gendered livelihood diversification in remote areas of Nigeria. Environ Sci Pollut Res. 2018;1–11.

208. World Bank. Literacy rate, adult total (% of people ages 15 and above) [Internet]. World Bank; 2017. Available from: http://data.worldbank.org/indicator/SE.ADT.LITR.ZS

209. CEPALSTAT. Social Indicators and Statistics: Education, literacy rate of people ages 15 years and over, by sex (Percentage) [Internet]. CEPALSTAT; 2016. Available from: http://interwp.cepal.org/sisgen/ConsultaIntegradaFlashProc_HTML.asp

210. PAHO. Health in the Americas: Regional Outlook and Country Profiles. Washington, D.C.; 2012. (Scientific and Technical Publication). Report No.: 636.

211. UNDP. Human Development Report 2003: Millennium Development Goals: A compact among nations to end human poverty [Internet]. Fukuda-Parr S, editor. Human Development. New York and Oxford: Oxford University Press; 2003. 1-368 p.

212. Gaible E. Survey of ICT and Education in the Caribbean [Internet]. Education. Washington, D.C.; 2009. (ICT and Education Series; vol. 2).

213. SALISES. Barbados Country Assessment of Living Conditions 2010 Volume 1: Human Development Challenges in a Global Crisis: Addressing Growth and Social Inclusion [Internet]. Vol. 1. Cave Hill, Barbados; 2012.

214. Forbes. The 200 Largest U.S. Charities [Internet]. 2017 [cited 2017 May 6]. Available from: https://www.forbes.com/lists/2011/14/200-largest-us-charities-11_rank-environment-animal.html

215. Wilkinson C, Souter D. Status of the Caribeean rocal reefs after bleaching and hurricanes in 2005. Global Coral Reef Monitoring Network. Townsville; 2008. 152 p.

216. Gumbs JC. Department of Fisheries and Marine Resources: Annual Report 2011. Anguilla; 2012.

217. Reef Check. Global Reef Tracker [Internet]. 2017 [cited 2017 Aug 8]. Available from: http://data.reefcheck.us/

218. Singh-Renton S, McIvor I. Review of current fisheries management performance and conservation measures in the WECAFC area [Internet]. FAO Fisheries and Aquaculture Technical Paper No. 587. 2015. 293 p.

219. Salas S, Chuenpagdee R, Charles A, Seijo JC. Coastal fisheries of Latin America and the Caribbean. FAO Fisheries and Aquaculture Technical Paper. Rome, Italy; 2011. Report No.: 544.

220. CRFM. Report of Third Annual Scientific Meeting – Kingstown, St. Vincent & the Grenadines, 17-26 July 2007 - National Reports [Internet]. Vol. 1. Kingstown, St. Vincent & the Grenadines; 2007.

221. Wilkinson C. Status of the Coral Reefs of the World: 2008. Townsville; 2008.

222. Gumbs KS, Wynne S, Johnson R. Anguilla Fisheries Development Plan. The Valley, Anguilla; 2015.

223. FAO. Fishery Country Profile: Antigua and Barbuda. FAO Fishery Country Profiles. Rome, Italy; 2007.

224. Pauly D, Ramdeen S, Ulman A. Reconstruction of total marine catches for Aruba, southern Caribbean, 1950-2010. Fisheries Centre Working Paper. Vancouver, B.C.; 2015. Report No.: 2015–10.

225. Gittens L. Bahamas National Report. In: CRFM, editor. Report of the Third Annual Scientific Meeting - Kingstown, St Vincent & the Grenadines, 17-26 July 2007 - National Reports. Kingstown, St. Vincent & the Grenadines; 2007. p. 5–7.

226. Mohammed E, Parker C, Willoughby S. Barbados: Reconstructed fisheries catches and fishing effort, 1940-2000. Fish Cent Res Reports. 2003;11(6):45–66.

227. Schep S, Johnson AE, Van Beukering P, Wolfs E. The fishery value of coral reefs in Bonaire. Amsterdam; 2012.

228. Valle S V., Sosa M, Puga R, Font L, Duthit R. Coastal fisheries of Cuba. In: Salas S, Chuenpagdee R, Charles A, Seijo JC, editors. Coastal fisheries of Latin America and the Caribbean. Rome: FAO Fisheries and Aquaculture Technical Paper 544; 2011. p. 155–74.

229. Lindop A, Bultel E, Zylich K, Zeller D. Reconstructing the former Netherlands Antilles marine catches from 1950 to 2010. Vancouver, B.C.; 2015. (Fisheries Centre Working Paper). Report No.: 2015–69.

230. Ramdeen R, Harper S, Zeller D. Reconstruction of Total Marine Fisheries Catches for Dominica (1950-2010). Fish catch Reconstr Islands, Part IV. 2014;22(Fentem 1960):33–41.

231. van der Meer L, Ramdeen R, Zylich K, Zeller D. Reconstruction of Total Marine Fisheries Catches for the Dominican Republic (1950-2010). Fish catch Reconstr Islands, Part IV Fish Cent Res Reports. 2014;22(2):33–41.

232. Leblond E, Daures F, Berthou P, Dintheer C. The Fisheries Information System of Ifremer: a multidisciplinary monitoring network and an integrated approach for the assessment of French fisheries, including small-scale fisheries. ICES Annu Sci Conf. 2008;22–6.

233. Ramdeen R, Belhabib D, Harper S, Zeller D. Reconstruction of Total Marine Fisheries Catches for Haiti and Navassa Island (1950–2010). In: Harper S, Zylich K, Boonzaier L, Le Manach F, Pauly D, Zeller D, editors. Fisheries catch reconstructions: Islands, Part III. University of British Columbia; 2012. p. 37–45.

234. Ramdeen R, Ponteen A, Harper S, Zeller D. Reconstruction of total marine fisheries catches for Montserrat (1950–2010). Fish Catch Reconstr Islands, Part III. 2012;20(5):69–76.

235. Bultel E, Lindop A, Ramdeen R, Zylich K. Reconstruction of marine fisheries catches for St. Barthélémy and St. Martin (French Caribbean, 1950-2010). Vancouver, B.C.; 2015. (Fisheries Centre Working Paper Series). Report No.: 2015–39.

236. Ramdeen R, Zylich K, Zeller D. Reconstruction of Total Marine Fisheries Catches for St. Kitts and Nevis (1950-2010). Working Paper Series 3. Vancouver, B.C.; 2013. Report No.: 04.

237. George SN, Peter S, Nelson T. Saint Lucia. In: Singh-Renton S, McIvor I, editors. Review of current fisheries management performance and conservation measures in the WECAFC area. Rome: Springer; 2015. p. 233–48.

238. Straker L. Saint Vincent and the Grenadines. In: Report of the Regional Workshop on the Monitoring and Management of Queen Conch, Strombus gigas, Kingston, Jamaica, 1-5 May 2006. FIMF/R832. Rome, Italy: FAO Fisheries Report; 2007. p. 152–8.

239. Mohammed E, Ferreira L, Soomai S, Martin L, Shing CCA. Coastal fisheries of Trinidad and Tobago. In: Salas S, Chuenpagdee R, Charles A, Seijo JC, editors. Coastal fisheries of Latin America and the Caribbean. Rome: FAO Fisheries and Aquaculture Technical Paper. No. 544.; 2011. p. 315–56.

240. Lockhart K. Turks and Caicos Islands National Report on the Queen Conch and Lobster Fisheries 2006/2007. In: CRFM, editor. Report of the Third Annual Scientific Meeting - Kingstown, St Vincent & the Grenadines, 17-26 July 2007 - National Reports. Kingstown, St. Vincent & the Grenadines; 2007. p. 57–62.

241. Tompkins EL, Adger WN. Does Adaptive Management of Natural Resources Enhance Resilience to Climate Change? Ecol Soc. 2004;9(2):10.

242. FAO. FAOLEX Database [Internet]. Food and Agriculture Organization of the United Nations; 2017. Available from: http://www.fao.org/faolex/en/

243. LSE. Climate Change Laws of the World [Internet]. London School of Economics’ Grantham Research Institute on Climate Change and the Environment; 2017. Available from: http://www.lse.ac.uk/GranthamInstitute/climate-change-laws-of-the-world/

244. DCNA. Policy & Legislation [Internet]. Dutch Caribbean Nature Alliance. 2014 [cited 2017 Aug 8]. Available from: http://www.dcnanature.org/resources/policy-law-enforcement/

245. Phillips GS, Emanuel E. Montserrat Sustainable Development Plan 2008-2020. 2005.

246. Schill S, Brown J, Justiniano A, Hoffman AM. US Virgin Islands Climate Change Ecosystem-Based Adaptation: Promoting Resilient Coastal and Marine Communities. 2014.

247. PRCCC. Ruta hacia la Resiliencia: Guia de estrategias para la adaptacion a los cambios climaticos. 2015.

248. Hodge KVD, Connor RA, de Berdt Romilly G. Transforming to a Climate-Resilient, Energy Efficient and Low Carbon Economy- Anguilla’s Climate Change Policy (DRAFT) [Internet]. 2011. (Technical Report). Report No.: 5C/ECACC-11-01-1.

249. Holling CS, editor. Adaptive Environmental Assessment and Management. New York, NY: John Wiley and Sons; 1978.

250. Walters C. Adaptive Management of Renewable Resources. New York, NY: Macmillan; 1986.

251. Government of Antigua & Barbuda. Antigua & Barbuda Intended Nationally Determined Contribution (INDC) [Internet]. 2015. Available from: https://www.cia.gov/library/publications/the-world-factbook/geos/ac.html

252. Government of Antigua & Barbuda. The Barbuda (Fisheries) Regulations, 2014. Antigua & Barbuda; 2014 p. 20.

253. NCCC & BESTC. National Policy for the Adaptation to Climate Change. Nassau, The Bahamas; 2005.

254. Government of the Bahamas. Fisheries Resources (Jurisdiction and Conservation) (Declaration of Protected Areas) (High Cay and Surrounding Waters) Order, 2012. The Bahamas; 2012.

255. Rawlins-Bentham J. Barbados National Climate Change Policy Approved. Barbados Government Information Service [Internet]. 2012 Apr; Available from: http://gisbarbados.gov.bb/blog/barbados-national-climate-change-policy-approved/

256. Government of Barbados. Fisheries Division [Internet]. Ministry of Agriculture. 2012 [cited 2017 Aug 1]. Available from: http://agriculture.gov.bb/agri/index.php?option=com_content&id=67&Itemid=82

257. Penn AB. The Virgin Islands Climate Change Adaptation Policy: Achieving Low-Carbon, Climate-Resilient Development. 2011. (Caribbean Community Climate Change Center Technical Report). Report No.: 5C/ECACC-11-10-1.

258. Government of St. Lucia. Closure of Sea Turtle Fishery. St. Lucia; 2011.

259. Government of the British Virgin Islands. Fisheries (Protected Species) Order, 2014. 28 British Virgin Islands; 2014.

260. NCCC. Achieving a Low Carbon Climate-Resilient Economy: Cayman Islands’ Climate Change Policy. 2011.

261. Government of the Cayman Islands. The Marine Conservation (Grouper Spawning Areas) Notice [Internet]. Cayman Islands; 2011. Available from: http://www.paho.org/saludenlasamericas/index.php?option=com_docman&task=doc_view&gid=120&Itemid=

262. Martinez-Hernandez L, Meneses YP. Aprueban Plan de Estado para el enfrentamiento al cambio climático. Granma [Internet]. 2017 Apr; Available from: http://www.granma.cu/cuba/2017-04-27/aprueban-plan-de-estado-para-el-enfrentamiento-al-cambio-climatico-27-04-2017-23-04-48

263. Government of Cuba. Resolución No. 244/2015. Cuba; 2016.

264. HEN. National Report of Curacao. In: The Third International Conference on Small Island Developing States. Apia, Samoa: Ministry of Health, Environment and Nature, Government of Curacao; 2014. p. 27.

265. Vermeij M, Chamberland V. Appendix: An overview of specific rules and regulations to protect Curacao’s marine life. 2009.

266. Homer F. Developing Pilot Projects for Climate Change Adaptation in Dominica: Report on the Technical Forum on Climate Change - Special Programme for Adaptation to Climate Change Project (SPACC). Roseau, Dominica; 2009. (Caribbean Community Climate Change Centre Technical Report). Report No.: 5C/SPACC-09-10-1.

267. SOFRECO. Fisheries Policy for Dominica, 2012-2037 [Internet]. ACP Fish II: Strengthening fisheries management in ACP states. 2012. Available from: http://linkinghub.elsevier.com/retrieve/pii/S1524904206000063

268. CNCCMDL. Estrategia Nacional para Fortalecer los Recursos Humanos y las Habilidades para Avanzar hacia un Desarrollo Verde, con Bajas Emisiones y Resiliencia Climática [Internet]. Santo Domingo, Dominican Republic; 2012. Available from: http://www.uncclearn.org/sites/default/files/estrategia_nacional_para_fortalecer_los_recursos_humanos_republica_dominicana_08_2012_0.pdf

269. Government of the Dominican Republic. Vedo de Langosta. Dominican Republic; 2015.

270. Belfon M. Grenada Strategic Program for Climate Resilience (SPCR) [Internet]. Grenada; 2011. Available from: http://dms.caribbeanclimate.bz/webinfo/DocumentList_cbz_pn.php?page=1&ipp=All&search=Grenada

271. Government of Grenada. Fisheries (Amendment) Act. Grenada; 2014.

272. ADEME. Schéma Régional Air Climat Energie: Guadeloupe [Internet]. Available from: http://www.guadeloupe.developpement-durable.gouv.fr/IMG/pdf/Rapport_SRCAE_261212.pdf

273. Government of Haiti. Programme Changements Climatiques République d’Haïti: Plan d’Action National d’Adaptation. 2006.

274. MWLECC. Climate Change Policy Framework and Action Plan [Internet]. 2013. (Green Paper). Report No.: 1/2013. Available from: http://www.japarliament.gov.jm/attachments/440_Climate Change.pdf

275. Government of Jamaica. The Fishing Industry (Amendment) Act. Jamaica; 2015.

276. CLIMPACT. Etude et évaluation des impacts, de la vulnérabilité et de l’adaptation de la Martinique au changement climatique. Martinique; 2012.

277. Government of St. Barthelemy. Reglementation de l’Exercice de la Peche Cotiere dans les Eaux de Saint-Barthelemy. St. Barthlemey; 2016.

278. STENAPA. Legislation handbook for the National and Marine Parks of St. Eustatius. 2008.

279. Island Planning Services. United Nations Conference on Sustainable Development (Rio+20) National Preparatory Process: St. Kitts and Nevis.

280. Droiterre Inc. & Associates. The 2nd National Communications Report of St. Christopher and Nevis under the United Nations Framework Convention on Climate Change (UNFCCC). St. Michael, Barbados; 2015.

281. MAMRC. National plan of action to prevent, deter and eliminate Illegal, Unreported and Unregulated (IUU) Fishing. St. Kitts & Nevis; 2015.

282. Government of St. Kitts & Nevis. About the Department of Marine Resources [Internet]. [cited 2017 Aug 16]. Available from: http://dmrskn.com/about/

283. PDEH. St. Lucia National Climate Change Policy and Adaptation Plan. 2003.

284. Government of Sint Maarten. Besluit Tijdelijk Visverbod Kraakbeenvissen (Decree on the Prohibition of Cartilaginous Fish). St. Maarten; 2011.

285. Government of St. Vincent & the Grenadines. Pilot Program for Climate Resilience (PPCR) [Internet]. Available from: https://www.climateinvestmentfunds.org/sites/default/files/ST_vincents_Phase_1_proposal_report.pdf

286. Government of St. Vincent & the Grenadines. Fisheries Policy [Internet]. Ministry of Agriculture, Forestry, Fisheries, Rural Transformation, Industry and Labour. 2017 [cited 2017 Aug 16]. Available from: http://www.agriculture.gov.vc/agriculture/index.php?option=com_content&view=article&id=102&Itemid=74

287. Government of Trinidad & Tobago. National Climate Change Policy [Internet]. 2011. Available from: http://www.ema.co.tt/new/images/policies/climate_change_2011.pdf

288. Mohammed E. Current Initiatives for Fisheries Management in Trinidad & Tobago [Internet]. Chaguaramas, Trinidad & Tobago; 2017. Available from: https://u.tt/uploads/05_Elizabeth_mohammed_FD_final_-_Current_Initiatives_for_Fisheries_Management_in_TT_30Jun17.pdf

289. Gordon A. Turks And Caicos Islands: National progress report on the implementation of the Hyogo Framework for Action (2011-2013) [Internet]. Turks & Caicos Islands; 2013. Available from: http://tandt.oxfordjournals.org/cgi/doi/10.1093/tandt/2.2.51

290. Government of Turks & Caicos Islands. Fisheries Protection Ordinance and Subsidiary Legislation Revised Edition. Turks & Caicos Islands; 2009.

291. Government of the United States Virgin Islands. Preparing the Virgin Islands of the United States for Adapting to the Impacts of Climate Change. Charlotte Amalie, USVI, US Virgin Islands; 2015.

292. Adger WN, Vincent K. Uncertainty in adaptive capacity. Comptes Rendus - Geosci. 2005;337(4):399–410.

293. World Bank. GINI index (World Bank estimate) [Internet]. 2017 [cited 2017 Aug 7]. Available from: http://data.worldbank.org/indicator/SI.POV.GINI

294. Kairi Consultants Ltd. The Cayman Islands National Assessment of Living Conditions (2006/2007). Vol. I. 2008.

295. Kairi Consultants Ltd. Country Poverty Assessment: Grenada, Carriacou and Petit Martinique Main Report. Vol. I. 2008.

296. Kairi Consultants Ltd. St. Vincent and the Grenadines Country Poverty Assessment: 2007. Vol. 1. 2008.

297. Kairi Consultants Ltd. Anguilla: Country Poverty Assessment 2007/2009: Main Report. Vol. 1. 2009.

298. Kairi Consultants Ltd. Country poverty assessment: St. Kitts and Nevis 2007/08: Living conditions in a Caribbean small island developing state. Vol. 1. 2009.

299. Halcrow Group Ltd. Final Report: Country Poverty Assessment, 2012. Vol. 1. 2014.

300. Halcrow Group Ltd. Country Poverty Assessment: The British Virgin Islands. Vol. 1. 2003.

301. Halcrow Group Ltd. Montserrat Survey of Living Conditions, 2009. 2012.

302. Copeland HE, Pocewicz A, Naugle DE, Griffiths T, Keinath D, Evans J, et al. Measuring the Effectiveness of Conservation: A Novel Framework to Quantify the Benefits of Sage-Grouse Conservation Policy and Easements in Wyoming. PLoS One. 2013;8(6):e67261.

303. Benhaddouche A. Guadeloupe: des disparités de patrimoine et de revenus marquées. 2014.

304. Noss A. Household Income for States: 2008 and 2009 [Internet]. 2010. Available from: https://www.census.gov/prod/2010pubs/acsbr09-2.pdf

305. La Chispa. Kwart bevolking Curaçao onder armoedegrens. La Chispa [Internet]. 2014 Dec 14; Available from: http://www.lachispa.eu/nieuws/kwart-bevolking-curacao-onder-armoedegrens/

306. Bulmer-Thomas V. The Economic History of the Caribbean since the Napoleonic Wars. Cambridge, UK: Cambridge University Press; 2012.

307. Kairi Consultants Ltd. Living Conditions in Antigua and Barbuda: Poverty in a Services Economy in Transition Volume II Voices of the Poor. Vol. I. 2007.

308. PAHO. Bahamas. Washington, D.C.; 2012. (Scientific & Technical Publication). Report No.: 636.

309. Kairi Consultants Ltd. Country Poverty Assessment: Dominica [Internet]. Vol. 1. 2010. Available from: http://www.caribank.org/uploads/publications-reports/economics-statistics/country-poverty-assessment-reports/Dominica+CPA+-+Main+Report+Final+%28Submitted%29.pdf

310. PAHO. Saint Lucia. Washington, D.C.; 2012. (Scientific & Technical Publication). Report No.: 636.

311. IUCN, UNEP-WCMC. The World Database on Protected Areas [Internet]. Cambridge, UK: UNEP World Conservation Monitoring Centre; 2016 [cited 2016 Jul 5]. Available from: www.protectedplanet.net

312. CaMPAM. CaribbeanMPA [Internet]. Caribbean Marine Protected Area Network and Forum. 2010 [cited 2016 Jul 5]. Available from: http://campam.gcfi.org/CaribbeanMPA/CaribbeanMPA.php

313. Marine Conservation Institute. MPAtlas [Internet]. 2015 [cited 2016 Jun 1]. Available from: www.mpatlas.org

314. WRI. Reefs at Risk Revisited [Internet]. Washington, D.C.; 2011 [cited 2016 Jul 5]. Available from: http://www.wri.org/publication/reefs-risk-revisited

315. NOAA Marine Protected Areas Center. U.S. Marine Protected Areas Boundaries: MPA Inventory [Internet]. 2014 [cited 2016 Jul 5]. Available from: http://marineprotectedareas.noaa.gov/dataanalysis/mpainventory/

316. ESRI. ArcGIS Desktop. Redlands, CA: Environmental Systems Research Institute; 2016.

317. Government of Anguilla. Revised Regulations of Anguilla under Fisheries Protection Act. Anguilla; 2000 p. 40.

318. Hoggarth D. Management Plan for the Marine Parks of Anguilla. St. Michael, Barbados; 2001.

319. Government of Antigua & Barbuda. The Fisheries Regulations, 2013. Antigua & Barbuda; 2013 p. 84.

320. Government of Aruba. Landsbesluit verbod jacht op koraalvissen. Aruba; 1992 p. 3.

321. Government of Aruba. Landsbesluit verboden onderwaterjachtmiddelen. Aruba; 2001 p. 1.

322. Theile S. Queen Conch Fisheries and Their Management in the Caribbean. 2001.

323. Government of the Bahamas. Fisheries Resources (Jurisdiction and Conservation). The Bahamas; 2010 p. 43.

324. Waugh GT, Braynen MT, Bethel WG, Gittens L. Five Year Sector: Strategic Plan, 2010-2014. Nassau, The Bahamas; 2010.

325. Government of the Bahamas. Fisheries Resources (Jurisdiction and Conservation) (Amendment) Regulations, 2015. The Bahamas; 2015.

326. Government of Barbados. Fisheries Management and Development. Barbados; 1995.

327. Barbados Fisheries Division. Barbados Fisheries Management Plan: Schemes for the Management of Fisheries in the Waters of Barbados, 2004-2006. 2003.

328. Mcmanus E, Lacambra C. Fishery Regulations in the Wider Caribbean Region: Project Summary [Internet]. 2004.

329. Government of Bonaire. Marine Environment Ordinance. Bonaire; 1991.

330. Meyer KD, MacRae D. Bonaire National Marine Park Management Plan 2006. Bonaire; 2006.

331. Government of Bonaire. Island Resolution Nature Management Bonaire. Bonaire; 2010.

332. Government of the British Virgin Islands. Virgin Islands Fisheries Regulations, 2003. British Virgin Islands; 2003.

333. Conservation & Fisheries Department. Fishing laws in the British Virgin Islands. Road Town, Tortola; 2015.

334. Cayman Islands Department of the Environment. Marine Park Regulations & Marine Conservation Laws. Government of the Cayman Islands; 2008. p. 2.

335. Turner JR, McCoy C, Cottam M, Olynik J, Timothy A, Blumenthal J, et al. Biology and ecology of the coral reefs of the Cayman Islands. In: Sheppard CRC, editor. Coral Reefs of the United Kingdom Overseas Territories22. Dordrecht: Springer; 2013. p. 69–88.

336. Government of the Cayman Islands. The National Conservation Law, 2013 [Internet]. Cayman Islands; 2014. Available from: http://www.paho.org/saludenlasamericas/index.php?option=com_docman&task=doc_view&gid=120&Itemid=

337. Government of Grenada. Fisheries Regulations. Grenada; 1987.

338. Baisre-Hernandez J. Cuban fisheries management regime: current state and future prospects. United Nations University; 2006.

339. Government of Dominica. Fisheries Act Chapter 61:60. Dominica; 1987.

340. Herrera A, Betancourt L, Silva M, Lamelas P, Melo A. Coastal fisheries of the Dominican Republic. In: Salas S, Chuenpagdee R, Charles A, Seijo JC, editors. Coastal fisheries of Latin America and the Caribbean. Rome: FAO Fisheries and Aquaculture Technical Paper. No. 544; 2011. p. 175–217.

341. Chakalall B. Fisheries management in the Lesser Antilles. In: Proceedings of the 42nd Gulf and Caribbean Fisheries Institute. 1995. p. 294–330.

342. Government of Haiti. Décret 27 Octobre 1978 sur la pêche. Haiti; 1978.

343. Creary M, Alcolado PM, Coelho V, Crabbe J, Green S, Geraldes F, et al. Status of coral reefs in the Northern Caribbean and Western Atlantic GCRMN Node in 2008. In: Wilkinson C, editor. Status of Coral Reefs of the World: 2008. Townsville, Australia: Global Coral Reef Monitoring Network and Reef and Rainforest Research Centre; 2008. p. 239–52.

344. UNEP-WCMC. Review of corals from Fiji, Haiti, Solomon Islands and Tonga. Cambridge; 2015.

345. Government of Jamaica. The Fishing Industry Act. Jamaica; 1976 p. 27.

346. Aiken K, Kong A, Smikle S, Appeldoorn R, Warner G. Managing Jamaica’s queen conch resources. Ocean Coast Manag Coast Manag. 2006;49:332–41.

347. Iborra Martin J. Fisheries in Martinique. Brussels; 2007.

348. Government of Montserrat. Montserrat Fisheries Act Revised. Montserrat; 2002.

349. Government of Puerto Rico. Puerto Rico Fishing Regulations. Puerto Rico; 2004 p. 1–45.

350. Dilrosun F. Monitoring the Saba Bank Fishery. Curacao; 2000.

351. Lundvall S. Saba Bank Special Marine Area Management Plan 2008. Saba; 2008.

352. Toller W, Lundvall S. Assessment of the Commercial Fishery of Saba Bank. 2008.

353. DCNA. St. Eustatius Marine Park Management Plan 2007. 2007.

354. DCNA. St. Maarten Marine Park Management Plan 2007. 2006.

355. Government of St. Kitts & Nevis. The Fisheries Act. St. Kitts & Nevis; 1984 p. 28.

356. Government of St. Kitts & Nevis. Fisheries Regulations 1995. St. Kitts & Nevis; 1995.

357. Joseph W. Report on the lobster fisheries of Saint Lucia. In: Second Workshop on the Management of Caribbean Spiny Lobster Fisheries in the WECAFC Area. Havana, Cuba; 2003. p. 145–61.

358. De Beauville-Scott S. St. Lucia National Report. In: CRFM Fishery Report, Volume 1, Supplement 1 Report of the Third Annual Scientific Meeting. Kingstown, St. Vincent & the Grenadines; 2007.

359. Blackman K, Mattai I. Environmental and Sustainable Development Legislation for the Grenadine Islands. Cave Hill, Barbados; 2007.

360. Government of Trinidad & Tobago. Fisheries act. Trinidad & Tobago; 2014.

361. Lockhart K. Turks and Caicos Islands National Report on the Queen Conch and Lobster Fisheries 2006-2007. Kingstown, St. Vincent & the Grenadines; 2007. (CRFM Fishery Report 2007).

362. Logan A, Sealey KS. The reefs of the Turks and Caicos Islands. In: Sheppard CRC, editor. Coral Reefs of the United Kingdom Overseas Territories. Dordrecht: Springer; 2013. p. 97–114.

363. DPNR. United States Virgin Islands Commercial & Recreational Fisher’s Information Handbook. Department of Planning and Natural Resources, Division of Fish and Wildlife, Government of the US Virgin Islands. US Virgin Islands Department of Planning & Natural Resources; 2012. p. 45.

364. Kaufmann D, Kraay A. The Worldwide Governance Indicators, 2016 Update [Internet]. World Bank; 2016. Available from: http://info.worldbank.org/governance/wgi/#home

365. R Core Team. R: A language and environment for statistical computing [Internet]. Vienna, Austria: R Foundation for Statistical Computing; 2017. Available from: https://www.r-project.org/

366. Venables WN, Ripley BD. Modern Applied Statistics With S [Internet]. New York: Springer; 2002.

367. Cribari-Neto F, Zeileis A. Beta regression in R. J Stat Softw. 2010;34(2):1–24.

368. Fox J, Weisberg S. An R Companion to Applied Regression. 2nd ed. Thousand Oaks, CA: Sage; 2011.

369. Barbosa AM, Brown JA, Jimenez-Valverde A, Real R. modEvA: Model Evaluation and Analysis. 2016.

1. For this purpose, we assumed that the Kramer and Chapman equation would not apply to taxa in the Carangidae, Clupeidae, Coryphaenidae, Hemiramphidae, Istiophoridae, and Scombridae families due to their pelagic habitats (50). [↑](#footnote-ref-1)
2. The data for Antigua & Barbuda, the Dominican Republic, St. Maarten, Trinidad & Tobago, St. Barthelemy, and St. Martin include only air arrivals (they do not include arrivals by yacht, ferry, or other small vessels). Additionally, the data for Puerto Rico comes from non-resident hotel registrations and thus does not include tourists who did not stay in hotels. [↑](#footnote-ref-2)
3. The CBS data for Bonaire, Saba, and St. Eustatius reported tourist arrivals by air and by boat. For Bonaire, we included tourists arriving by air or yacht but excluded the 156,000 cruise ship arrivals in 2014. For St. Eustatius, we included both air and sea arrivals because large cruise ships do not stop in St. Eustatius (although one small cruise ship did moor at the island in 2014). For Saba, we included both air and sea arrivals because Saba does not receive cruise ships. However, some of the sea arrivals may have been residents returning home via ferry. [↑](#footnote-ref-3)
